# Rhizobia use a pathogenic-like effector to hijack leguminous nodulation signalling

**DOI:** 10.1038/s41598-021-81598-6

**Published:** 2021-01-21

**Authors:** Safirah Tasa Nerves Ratu, Albin Teulet, Hiroki Miwa, Sachiko Masuda, Hien P. Nguyen, Michiko Yasuda, Shusei Sato, Takakazu Kaneko, Makoto Hayashi, Eric Giraud, Shin Okazaki

**Affiliations:** 1 United Graduate School of Agricultural Science, Tokyo University of Agriculture and Technology, Saiwaicho 3-5-8, Fuchu, Tokyo 183-8509 Japan; 2 Laboratoire Des Symbioses Tropicales Et Méditerranéennes, Institut de Recherche Pour Le Développement, UMR Institut de Recherche Pour Le Développement/SupAgro/Institut National de Recherche Pour L’Agriculture, L’Alimentation Et L’Environnement, Université de Montpellier/Centre de Coopération Internationale en Recherche Agronomique Pour Le Développement, 34398 Montpellier Cedex 5, France; 3 Department of International Environmental and Agricultural Science, Graduate School of Agriculture, Tokyo University of Agriculture and Technology, Saiwaicho 3-5-8, Fuchu, Tokyo 183-8509 Japan; 4 Graduate School of Life Sciences, Tohoku University, Katahira, Aoba-ku, Sendai, Miyagi 980-8577 Japan; 5 Faculty of Life Sciences, Kyoto Sangyo University, Motoyama, Kamigamo, Kita-Ku, 603-8555 Japan; 6 Center for Sustainable Resource Science, RIKEN, 1-7-22 Suehiro-cho, Tsurumi-ku, Yokohama City, Kanagawa 230-0045 Japan

**Keywords:** Rhizobial symbiosis, Symbiosis

## Abstract

Legume plants form a root-nodule symbiosis with rhizobia. This symbiosis establishment generally relies on rhizobium-produced Nod factors (NFs) and their perception by leguminous receptors (NFRs) that trigger nodulation. However, certain rhizobia hijack leguminous nodulation signalling via their type III secretion system, which functions in pathogenic bacteria to deliver effector proteins into host cells. Here, we report that rhizobia use pathogenic-like effectors to hijack legume nodulation signalling. The rhizobial effector Bel2-5 resembles the XopD effector of the plant pathogen *Xanthomonas campestris* and could induce nitrogen-fixing nodules on soybean *nfr* mutant. The soybean root transcriptome revealed that Bel2-5 induces expression of cytokinin-related genes, which are important for nodule organogenesis and represses ethylene- and defense-related genes that are deleterious to nodulation. Remarkably, Bel2-5 introduction into a strain unable to nodulate soybean mutant affected in NF perception conferred nodulation ability. Our findings show that rhizobia employ and have customized pathogenic effectors to promote leguminous nodulation signalling.

## Introduction

Symbiotic interactions between legume plants and soil bacteria, collectively called rhizobia, result in the formation of nitrogen-fixing root nodules on host plant roots. In nodules, rhizobia fix atmospheric dinitrogen as ammonia to provide host plants with nitrogen nutrition. In exchange, the host plants provide steady carbon sources and essential nutrients as well as an appropriate environment required for bacterial metabolism and development^1^. Due to this symbiosis, legumes play a critical role in agriculture and global nitrogen cycles^2,3^.

Root-nodule symbiosis involves highly host-specific interactions—a rhizobium species can nodulate only limited legume-plant species—and this specificity is determined by several layers of signal exchange between rhizobia and host plants^4^. First, plants secrete flavonoids from roots that are specific to plant species; their recognition by the transcriptional regulator NodD activates the expression of nodulation (*nod*) genes, leading to the synthesis of rhizobial signal molecules, named Nod factors (NFs), which are lipochitooligosaccharides harbouring species-specific modifications. NF recognition by plant LysM receptors at the plasma membrane activates symbiotic signalling in the host, leading to nodule formation and bacterial infection^5,6^. Second, bacterial extracellular polysaccharides, such as exopolysaccharides (EPSs), lipopolysaccharides (LPSs), and capsular polysaccharides (KPSs), function as another layer of host-specificity determinants^7–9^.

A third specific layer was shown to be constituted by rhizobial effector proteins secreted by type III/IV secretion systems (T3SS/T4SS), providing cultivar-specific and strain-specific incompatibility between two partners^10^. T3SS/T4SS were originally identified in pathogenic bacteria, and the secreted proteins (T3SS effectors; T3Es) were reported to function in bacterial infection and pathogenicity. Many rhizobia also possess functional T3SS/T4SS that secrete various effectors upon induction by host flavonoids^11^. Depending on the host genotype, rhizobial effectors can favour or impede root nodule formation. For example, in *Ensifer fredii* (*S. fredii*) NGR234, NopM acts as an E3 ubiquitin ligase and promotes nodulation by potentially reducing reactive oxygen species (ROS) generation in host plants during infection, whereas NopL can serve as a mitogen-activated protein kinase (MAPK) substrate and interfere with MAPK signalling to prevent premature nodule senescence^12,13^. In contrast, two effectors of *Bradyrhizobium elkanii* USDA61, InnB and Bel2-5, negatively affect nodule formation in some legume plants^14,15^. The negative effects of effectors frequently result from the induction of the host defence response called effector-triggered immunity (ETI), which occurs after the perception of effectors by plant resistance [R] proteins. ETI is often associated with a hypersensitivity response that halts rhizobial infection. These few examples illustrate that a root-nodule symbiosis requires evasion or suppression of the host immune responses, and thus, the strategy of legume infection by rhizobia displays some similarity with those used by plant pathogens^16^.

In addition to modulating plant immunity, the T3SS in some *Bradyrhizobium* strains was shown to activate nodulation processes in some legume species in the absence of NF signalling^17–19^. This effect was observed for *Bradyrhizobium* sp. ORS3257, which induces nodule formation on *Aeschynomene indica* not by NF signalling but via a cocktail of effectors that play synergistic and complementary roles. Remarkably, it was shown that ectopic expression of one of these effectors (ErnA) in transgenic *A. indica* lines activates organogenesis of root and nodule-like structures^19^. A similar scenario was also described for nodulation of a soybean *nfr1* mutant that can be induced by a USDA61 mutant unable to synthesize NFs, but that has a functional T3SS, suggesting that T3Es can bypass NF perception. One possibility would be that an as-yet-unidentified T3 effector activates NF signalling and induces the expression of symbiotic genes, such as *ENOD40* and *NIN*^17^. In these two cases, root hair curling and infection threads, two typical NF-induced responses, were not observed, which suggests that some T3Es can promote intercellular infection^17,18^.

To advance our understanding of this T3SS hijacks NF signalling, we identified the effectors governing nodule organogenesis during the symbiosis between USDA61 and soybean plants. We found that the effector Bel2-5, which resembles XopD, a T3E of plant pathogenic *Xanthomonas* bacteria, is a key factor of this alternative symbiotic process. The effector targets the plant nucleus and encodes an ubiquitin-like protease 1 (ULP1) domain which is required for triggering nodulation. More interestingly, Bel2-5 not only dampens several plant defence-related responses, as a phytopathogenic effector, but also plays a distinct role in the induction of a complete set of cytokinin biosynthesis-related genes essential for nodule organogenesis.

## Results

### Screening of T3E candidates trigger nodulation on *nfr1* mutant soybean

**Figure 1:**
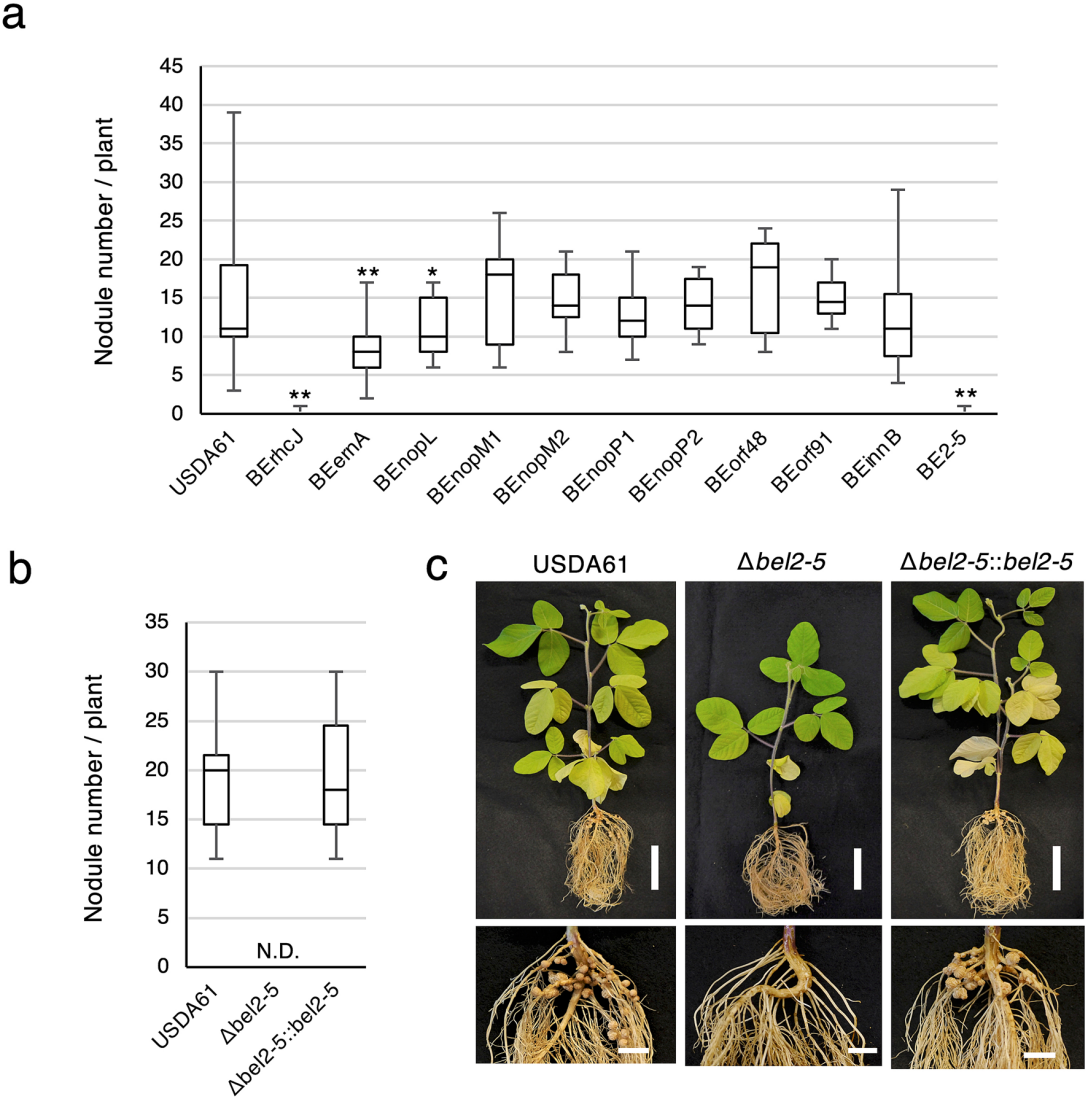
Nodulation of *G. max* cv. En1282 (*nfr1* mutant soybean) inoculated with wild-type and *nops* mutant strains of *B. elkanii* USDA61. (**a**) The number of nodules formed on En1282 inoculated with USDA61 wild-type and *nops* mutant candidates at 30 days after inoculation (dai). Values represent mean ± SD (*n* = 36 for USDA61, from four independent inoculation tests; *n* = 27 for BErhCJ; *n* = 29 for BEernA; *n* = 25 for BEnopL and BEnopM1; *n* = 23 for BEnopM2; *n* = 21 for BEnopP1; *n* = 19 for BEnopP2 and BEinnB; *n* = 20 for BEorf48, *n* = 22 for BEorf91, and *n* = 24 for BE2-5; from three independent inoculation tests for all mutants). (**b**) The number of nodules formed on En1282 inoculated with *bel2-5* mutant of *B. elkanii* strains at 30 dai. Values represent mean ± SD (*n* = 15, from three independent inoculation tests). N.D. means not detected. (**c**) En1282 plants inoculated with *B. elkanii* and *bel2-5* mutant strains. Plants were photographed at 30 dai. Scale bars: 5 cm for whole plants and 1 cm for roots. *p < 0.05 and **p < 0.001 by two-tailed Student *t*-test compared to USDA61 wild-type (**a**,**b**).

Previously, we reported that *B. elkanii* USDA61 harbours a unique set of T3Es that can hijack soybean NF signalling to promote infection^17^. We hypothesized that specific T3E(s) activate a series of symbiosis signalling steps, leading to efficient nodulation on *nfr1* mutant soybean. It was previously shown that the ErnA effector of the USDA61 strain plays a key role during symbiosis with *A. indica* and that the *ernA* mutant displays an apparent nod minus phenotype in this legume species^19^. We first analysed the effect of the *ernA* mutant, designated as BEernA, on En1282 (*nfr1*-mutant soybean). As shown in Fig. 1a, the nodulation properties of the *ernA* mutant on En1282 plants were significantly affected, but nodules were still formed at ~ 60% of the rate for the wild-type (WT) strain, indicating that other effector(s) could play a more prominent role in nodulation.

To identify the responsible effector, we generated mutants of USDA61 in putative effectors previously recognized based on two in silico searches, (1) genome TBLASTN searches for effector homologues in reference to identified rhizobial T3Es and (2) *tts* box searches for unidentified effectors putatively regulated by *ttsI*, a specific modulator of rhizobial T3SSs^20,21^, using the USDA61 genome (accession nos. AP013103 and AP013104). By combining these approaches, 54 putative effectors were predicted (Supplementary Table 1). Considering the large number of effector candidates, we decided to focus our mutagenesis approach on (1) the homologues of effector genes that were previously reported to have symbiotic roles in other rhizobia (*nopL*, *nopM*, and *nopP*) and (ii) USDA61-specific putative effector genes (*orf48* and *orf91*)^22^. Thus, we mutated 7 putative effectors and designated the mutants BEnopL, BEnopM1, BEnopM2, BEnopP1, BEnopP2, BEorf48, and BEorf91. As certain rhizobial effectors can affect symbiosis positively or negatively with different host plants^23–25^, we also included in this analysis two previously obtained mutants in T3Es shown to negatively impact the symbiotic interaction with specific legumes: (1) a deletion mutant of InnB, which was shown to be responsible of the incompatibility of USDA61 with *Vigna radiata* KPS1 plants^14^, and (2) a transposon mutant in the putative effector Bel2-5, named BE2-5; the Bel2-5 effector was shown to be responsible for the incompatibility of USDA61 with *Rj4* soybean plants^15^.

On these additional 9 mutants tested, BEnopL and BE2-5 displayed symbiotic phenotypes (Fig. 1a). The *nopL* mutant induced ~ 20% less nodule formation than the WT strain, while the Tn5 *bel2-5* mutant displayed a strict nod minus phenotype, as observed for a mutant in the T3SS machinery (BErhcJ) (Fig. 1a). To confirm that the phenotype of BE2-5 was not due to a polar effect of the Tn5 insertion, we constructed a deletion mutant of this gene and complemented it with the *bel2-5* gene, designated Δ*bel2-5* and Δ*bel2-5::bel2-5,* respectively. The *bel2-5* deletion abolished En1282 nodulation, and furthermore, the nodulation phenotype was restored in plants inoculated with the complemented *bel2-5* mutant strain, resembling that observed with WT USDA61 (Fig. 1b,c). Together, these results demonstrated that Bel2-5 plays a prominent role to promotes nodulation on *nfr1* soybean, while the ErnA and NopL effectors appear to be less essential during symbiosis with this host plant.

### Bel2-5 is conserved among T3SS-harbouring rhizobia and plant pathogenic bacteria

**Figure 2:**
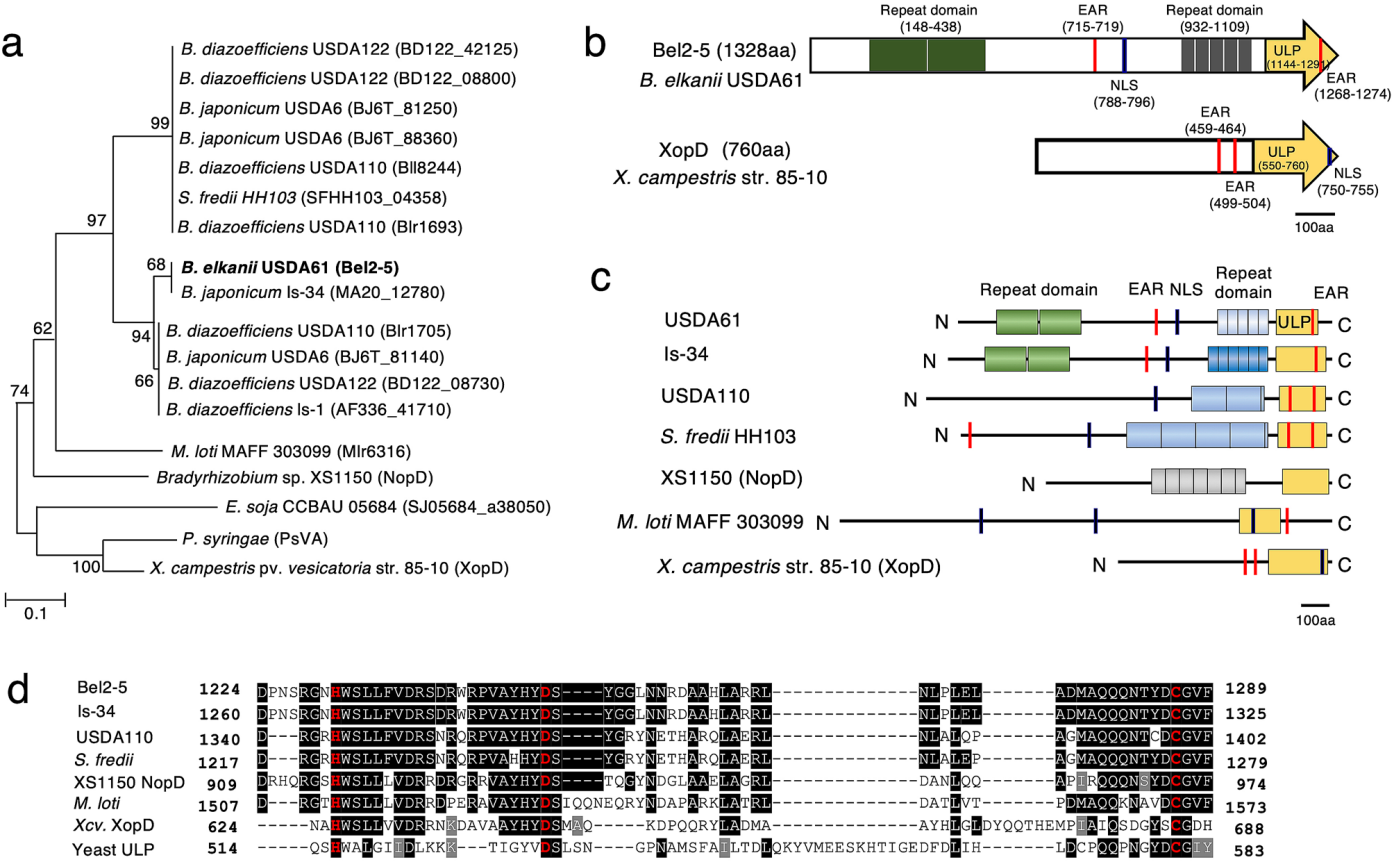
Phylogenetic and In silico analyses of *B. elkanii* Bel2-5 and its homologs. (**a**) Neighbor-joining tree of Bel2-5 and its homologs in T3SS-harbouring rhizobia and phytopathogenic bacteria. Boostrap values are given at the nodes with boostrap resampling (1000 replicates of each). (**b**) Schematic representation of Bel2-5 and XopD from *X. campestris* str. 85–10. (**c**) Schematic representation of Bel2-5 functional domains in *B. elkanii* USDA61, in comparison to its homologs in selected rhizobia and pathogenic bacteria. The repeat domain I and II, EAR motifs, NLS motif and ULP1 domain are shown. (**d**) Sequence alignment of ULP1 catalytic domain of *B. elkanii* BEL2-5 and its selected homologs, including: *B. japonicum* Is-34 (MA20_12780), *B. diazoefficiens* USDA110 (Bll8244), *S. fredii* HH103 (SFHH103_04358), *Bradyrhizobium* sp. XS1150 (NopD), *M. loti* MAFF 303,099 (Mlr6316), *X. campestris* pv. *vesicatoria* str. 85–10 (XopD), and yeast ULP1 (DAA11408.1). H, D and C catalytic core residues are indicated by red. Identical amino acids are shaded in black, and similar amino acids are shaded in gray, respectively.

Bel2-5 was predicted to possess a T3SS-related function, considering the presence of a *tts* box in its promoter region (Supplementary Data 1a). Furthermore, the first 50 aa residues of Bel2-5 feature a high proportion of Ser and a low proportion of Leu and Lys, and the fourth residue in Bel2-5 is also preceded by a Pro (Supplementary Data 1b), thus satisfying the criteria for a T3E^26,27^. BLASTP searches revealed that Bel2-5 is distributed among rhizobia, including *Bradyrhizobium*, *Sinorhizobium* (*Ensifer*), and *Mesorhizobium* species. However, Bel2-5 seems not to be a rhizobium-specific effector since it shares some similarities with XopD and XopD-like PsvA effectors from the phytopathogenic bacteria *Xanthomonas campestris* pv. *vesicatoria* (*Xcv.*) str. 85–10 (DAA34040.1; 26.11% identity) and *Pseudomonas syringae* pv*. eriobotryae* (BAA87062.1; 25.60% identity) in the C-terminal region of the two proteins, respectively (Fig. 2).

A putative nuclear localization signal (NLS; RPAKRPRTL) was detected at 788–796 aa in Bel2-5 using NLS mapper^28^ (Fig. 2b,c; Supplementary Data 1b), which indicates potential nuclear translocation. Interestingly, ErnA and XopD also contain putative NLSs, and their localization to the plant nucleus has been demonstrated^19,29^. Two sets of tandem repeat sequences with unknown function were found at 148 to 438 aa (containing two repeats of 146 residues) and at 932 to 1109 aa (containing five repeats, with four first repeats consist of 36 residues, and last repeat consists of 33 residues) of Bel2-5 (Fig. 2b,c; Supplementary Data 1b). Sequence analysis study with selected Bel2-5 homologs showed that repeat domains are present only among rhizobia with diverse in length and number of repetitions (Fig. 2c; Supplementary Data 1b).

The C-terminus of Bel2-5 is related with *Xcv.* XopD contained the small ubiquitin-like modifier (SUMO) protease domain of the C48 peptidase [ubiquitin-like protease 1 (ULP1)] (Fig. 2b,c; Supplementary Data 1b). ULP1, a cysteine protease first isolated from yeast (*Saccharomyces cerevisiae*)^30^, dynamically and reversibly regulates interactions between SUMO and its protein targets. In-silico analysis specifically on the C-terminal of Bel2-5 (from 1144 to 1328aa) showed that ULP1 domain is widely distributed among prokaryotic and eukaryotic species, yeast (Fig. 2b–d; Supplementary Data 1b,2a). Recently, NopD of *Bradyrhizobium* sp. XS1150 appears as characterized rhizobial SUMO protease facilitates a symbiotic efficiency^31^. ULP1 active-site function depends on the sequentially ordered catalytic-core triad His–Asp–Cys^32^. Alignment of yeast ULP1 (DAA11408.1) with Bel2-5 putative ULP1 domain and its selected homologs, including XopD and NopD showed that this triad is conserved within Bel2-5 residues (H1231/D1251/C1286) (Fig. 2d; Supplementary Data 2a).

Further analysis of the Bel2-5 coding sequence indicated the presence of two putative ethylene-responsive element-binding factor-associated amphiphilic repression (EAR) motifs (LxLxL) at 715–719 and 1268–1274 aa (Fig. 2b,c; Supplementary Data 1b). In plants, EAR motif-containing proteins are the principal transcriptional regulators responsible for repressing gene transcription, and this process is frequently necessary for modulating plant defence and stress responses^33,34^. Notably, *Xcv.* XopD possesses two EAR motifs [(L/F)DLN(L/F)(x)P] that are both essential for repressing defence-related genes and maximizing *Xcv*. growth in tomato plants^35,36^. The Bel2-5 effector therefore appears to be a modular protein consisting of multiple conserved domains found in T3Es originating from pathogenic and symbiotic bacteria^37^.

### Bel2-5 is a T3SS-secreted effector

**Figure 3:**
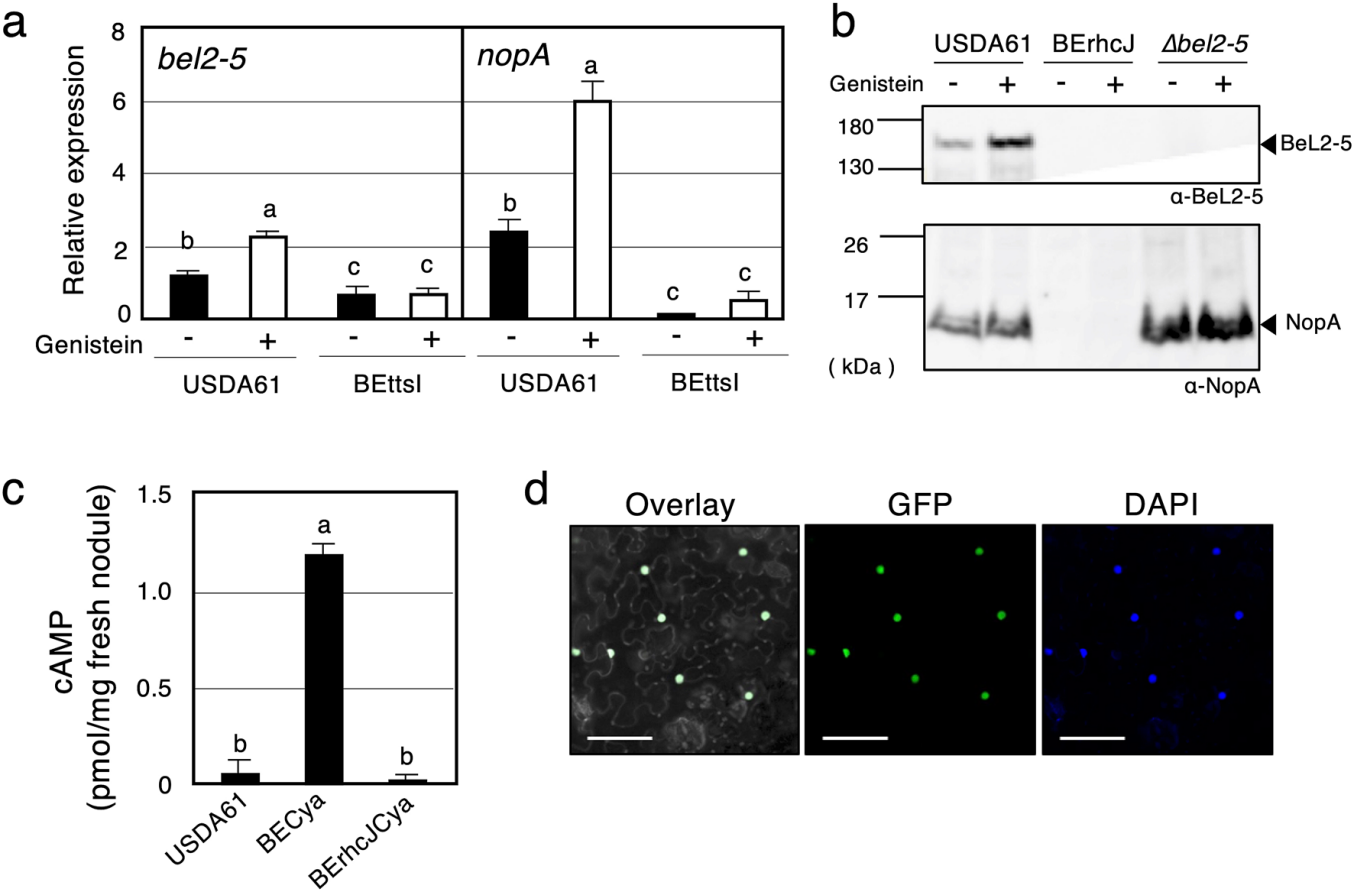
Expression, secretion, translocation and localization of the *B. elkanii* type III effector Bel2-5. (**a)** RT-qPCR analysis of *bel2-5* and *nopA* expressions in wild-type and *ttsI* mutant (BEttsI) of *B. elkanii* USDA61. Total RNAs were isolated from cultures in the absence (−) or presence (+) of the genistein (10 µM) after 48 h induction. The expression level of each gene was normalized by the ATP synthase (*atpD*) gene. Values represent mean ± SD (*n* = 3). (**b**) Secretion of Bel2-5 and NopA in the culture supernatant of *B. elkanii* strains. Secreted proteins from culture supernatants were subjected to immunoblot analysis with the anti-Bel2-5 (α-Bel2-5) or anti-NopA (α-NopA) antibodies. Molecular masses (kDa) of the marker are shown on the left. (**c**) Translocation of Bel2-5 into soybean nodules. cAMP levels measured from *G. max* cv. Enrei nodules harvested at 18 dpi. Plants were inoculated with *B. elkanii* USDA61 wild-type, BECya and BErhcJCya. The USDA61 wild-type contained no *cya* fusion. Values represent mean ± SD from duplicates measurements. (**d**) Bel2-5 is targeted to the plant nuclei. Bel2-5 protein fused with eGFP was transiently expressed in *N. benthamina* leaves by *Agrobacterium* infiltration and visualized 48 h after by confocal microscopy to determine the subcellular localization. From left to right: an overlay of GFP and DAPI fluorescence spectrums, and the GFP and DAPI fluorescence spectrum, respectively. Staining with DAPI was used to visualize nuclei. On the overlay panel, the DAPI fluorescence was displayed in gray and the contrast and brightness were enhanced in order to be able to visualize the contour of the cells. Scale bars: 100 µm. Means followed by different letters are significantly different at the 0.05 level by Tukey’s method (**a**,**c**).

In rhizobia, the expression of genes encoding T3Es and T3SS components is regulated by the transcriptional activator TtsI and host-derived flavonoids, such as genistein^10,11^. TtsI directly binds to a *cis*-element *tts* box in the promoter region of *tts* clusters upon induction by host flavonoids^20^. To determine whether *bel2-5* expression is regulated similarly, we examined its expression in the presence/absence of genistein and TtsI. The expression of *nopA*, one of the major T3SS machinery components^38,39^, was also quantified as a control. Transcriptional levels of *bel2-5* and *nopA* were significantly increased upon induction with genistein in the WT background. In the *ttsI* mutant background, the expression of both *nopA* and *bel2-5* genes tested was very weak even in the presence of genistein (Fig. 3a). Collectively, our results confirmed that the expression of *bel2-5*, similar to that of other T3SS-related genes, was regulated by genistein and TtsI.

To examine the T3SS-mediated secretion of Bel2-5 along with other secreted effectors, an extracellular protein analysis was conducted. For this purpose, the WT USDA61 strain and the *bel2-5* deletion and BErhcJ mutant strains were grown in the presence/absence of genistein, and the secreted proteins were harvested at 48 h post-induction. The generated secreted proteins were used for western blotting analysis of Bel2-5 using an anti-Bel2-5 antibody and led to the detection of a predicted Bel2-5 band (~ 146 kDa), which was observed only in USDA61 cultures with the enhancement of band intensities by the addition of genistein, and none were detected in the T3SS mutant (BErhcJ) and *bel2-5* mutants (Fig. 3b). To exclude the possibility that the deletion of *bel2-5* impacts the secretion of other effectors, in other words, that Bel2-5 participates in the functioning of the T3SS machinery, we also analysed the presence of the NopA in the culture supernatants of USDA61, the Bel2-5 mutant and T3SS machinery deficiency (BErhcJ) mutants with an anti-NopA antibody. As shown in Fig. 3b, NopA was detected in both the WT strain and the *bel2-5* mutant but not in the BErhcJ mutant, demonstrating that the secretion of the other T3Es was not blocked in the *bel2-5* mutant. Collectively, these data confirm that Bel2-5 is a T3SS-secreted protein. Although the signal intensity of the band corresponding to the Bel2-5 or NopA protein was altered by the addition of genistein, their presence could also be detected in cultures without genistein. These results are consistent with previous results^14,22^, suggesting that T3SS is activated in a genistein-independent manner under the test conditions. Therefore, unidentified regulators or inducers may be involved in the regulation of the USDA61 *tts* genes.

To confirm that Bel2-5 is translocated into host cells, we used an adenylate cyclase (Cya) reporter system. Here, the Bel2-5 C-terminus was fused to the calmodulin-dependent adenylate cyclase domain of Cya, and thus, if *bel2-5cya* was translocated into cells, cAMP synthesized by Cya due to calmodulin, which is present only in eukaryotic cells, could be detected^40^. The *bel2-5cya* hybrid was introduced into the WT USDA61 and BErhcJ strains to generate BECya and BErhcJCya, respectively, and cAMP levels were measured from fresh *Glycine max* cv. Enrei nodules inoculated with the mutants at 18 days post-inoculation (dpi) (Fig. 3c); cAMP accumulation was substantially increased in nodules infected with BECya but not BErhcJCya or USDA61 lacking *cya* (Fig. 3c), which confirmed that Bel2-5 was translocated into plant cells and that the T3SS machinery was essential for its translocation.

The presence of an NLS motif at 788–796 aa in Bel2-5 indicated potential nuclear translocation of the protein (Fig. 2b,c; Supplementary Data 1b). To check whether Bel2-5 targets the nuclei of plant host cells, we constructed a Bel2-5 C-terminally fused with enhanced GFP (Bel2-5-eGFP) and transiently expressed it in *Nicotiana benthamiana* leaves using *Agrobacterium tumefaciens*-mediated transformation. Confocal analysis performed 48 h after infiltration confirmed the nuclear localization of Bel2-5, as the GFP fluorescence signal colocalized specifically with the DAPI signal (a nuclear staining) in the nucleus of the cells (Fig. 3d). This result indicate that Bel2-5 is translocated into the host cell and targeted to the plant nucleus.

### The mutation in ULP1-like domain of Bel2-5 impairs nodule formation on *nfr1* mutant soybean

**Figure 4:**
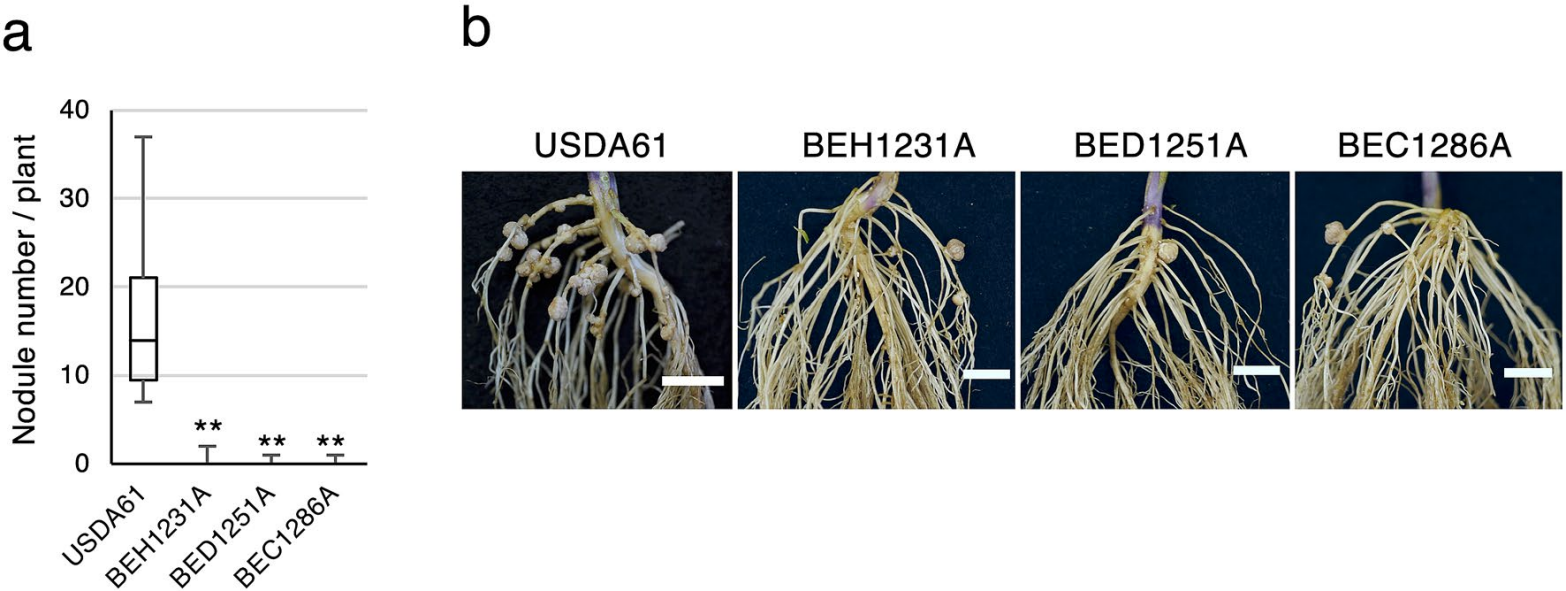
Nodulation of *G. max* cv. En1282 (*nfr1* mutant soybean) inoculated with Bel2-5 ULP1 domain mutant strains. (**a**) The number of nodules formed on En1282 inoculated with wild-type and the mutants of predicted catalytic core of Bel2-5 ULP1 at 30 dai. Values represent mean ± SD (*n* = 15, from three independent inoculation tests). **p < 0.001 by two-tailed Student *t*-test compared to USDA61 wild-type. (**b**) Roots of En1282 inoculated with wild-type and the mutants of predicted catalytic core of Bel2-5 ULP1 were photographed at 30 dai. Scale bars: 1 cm.

Because Bel2-5 was found to carry a C-terminal cysteine protease (ULP1-like) domain (Fig. 2b–d; Supplementary Data 1b and 2a), we suspected that the domain might play a role during symbiosis. The effector protease functions reveal that proteolysis of a host substrate is an important strategy employed by pathogens to alter host physiology^41^, and thus, proteolysis may have an essential role in symbiont-plant interactions. To examine whether the ULP1-like domain is required for Bel2-5-promoted symbiosis, we performed substitution analysis of predicted triad catalytic residues, His (H1231), Asp (D1251), and Cys (C1286), with Ala and named these: BEH1231A, BED1251A, and BEC1286A, respectively. A nodulation test on En1282 plants revealed that in contrast to the WT USDA61, which formed nodules, the mutants of ULP1 catalytic residues had drastically reduced nodulation capacity (Fig. 4). These results suggest that ULP1 domain of Bel2-5 effector is required for nodulation on *nfr1* mutant soybean.

### Heterologous Bel2-5 expression confers hijacking activity to other rhizobia

**Figure 5:**
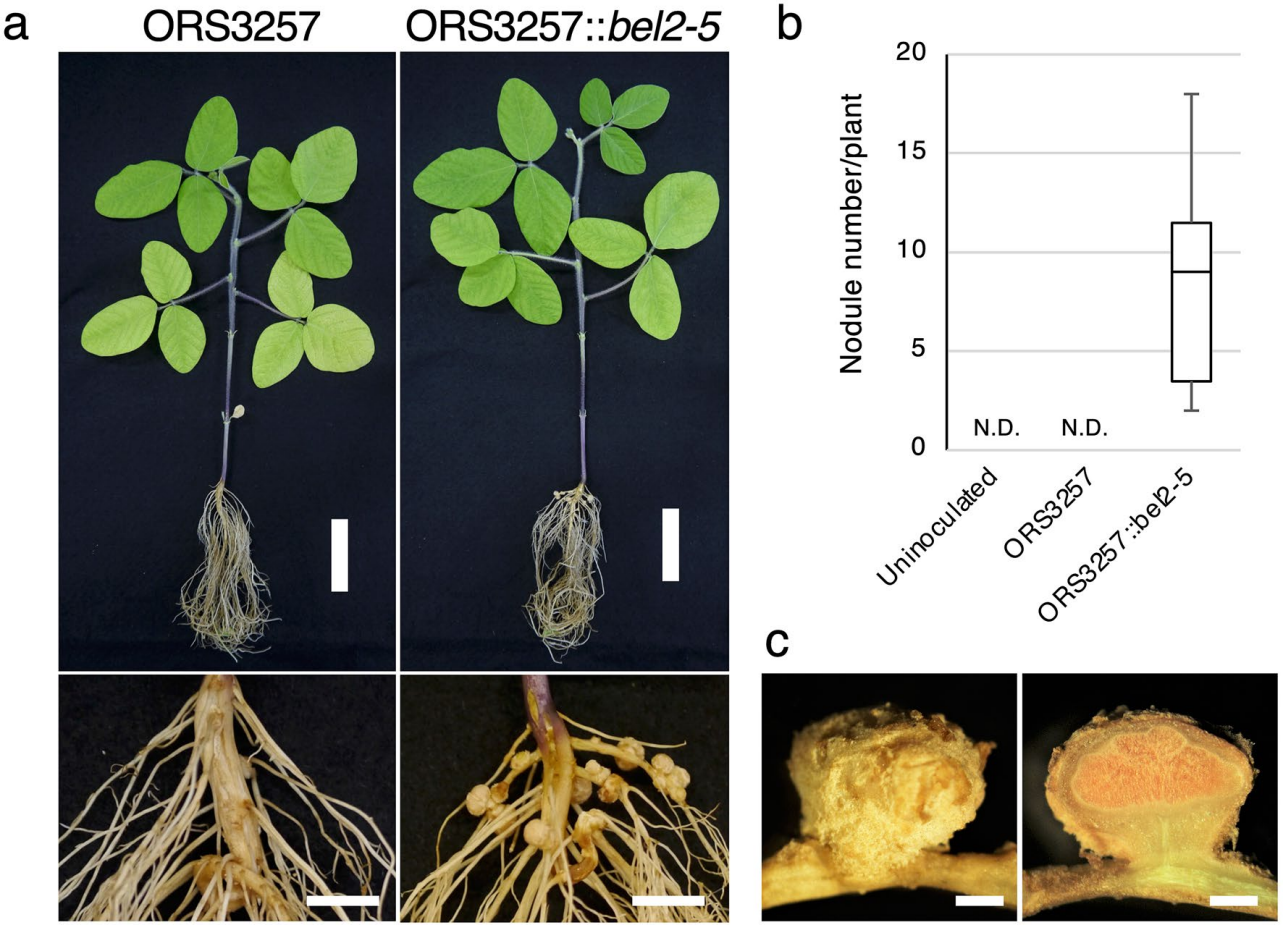
Bel2-5 confers hijacking activity to another rhizobia. (**a**) *G. max* cv. En1282 (*nfr1* mutant soybean) inoculated with *Bradyrhizobium* sp*.* ORS3257 wild-type and with insertion of *bel2-5* gene, designed as ORS3257::*bel2-5*. Whole plants (scale bars: 5 cm) and root nodules (scale bars: 1 cm) were photographed at 30 dpi. (**b**) The number of nodules formed on En1282 inoculated with uninoculated (water), ORS3257 and ORS3257::*bel2-5*. Nodules were counted at 30 dai. Values represent mean ± SD (*n* = 18, from three independent inoculation tests). N.D.: Not detected. (**c**) nodules section of En1282 inoculated with ORS3257::*bel2-5.* Scale bars: 1 mm.

*Bradyrhizobium* sp. ORS3257 possesses a functional T3SS and forms nodules on *A. indica* in a T3SS-dependent manner due to secretion of the ErnA effector^19^. We investigated in this study whether this strain was also able to nodulate En1282 soybean roots. As shown in Fig. 5a,b, En1282 plants inoculated with the ORS3257 strain did not form nodules, suggesting that USDA61 and ORS3257 differ in their mode of action to induce NF-independent symbiosis in these two host plants. ErnA and Bel2-5 share some similarities with a conserved 85 aa domain of unknown function displaying 35% identity (located at positions 706 and 790 aa in Bel2-5) (Supplementary Data 2b), but the similarities stop at this level and ErnA lacks of ULP1-like domain that we confirmed important for Bel2-5 triggering nodules formation in *nfr1* soybean roots. To verify Bel2-5 as the genuine effector that activates nodulation signalling in *nfr1* soybean plants, *bel2-5* was introduced into ORS3257 to generate ORS3257::*bel2-5*, which was then used (together with WT ORS3257) for En1282 nodulation tests. The introduction of *bel2-5* conferred the ability of ORS3257 to form nodules on En1282 soybean roots (Fig. 5). Nodule sections displayed the characteristic pink colour of leghemoglobin, suggesting that the nodules elicited by ORS3257::*bel2-5* were functional and able to fix nitrogen, as are those induced by USDA61 (Fig. 5c). These results indicate that a gain of function in the ability to nodulate soybean plants in the absence of NF signalling is possible by transferring *bel2-5* to another *Bradyrhizobium* strain, confirming that Bel2-5 is the key effector governing this symbiotic interaction.

### Bel2-5 regulates soybean symbiosis- and defence-related genes

**Figure 6:**
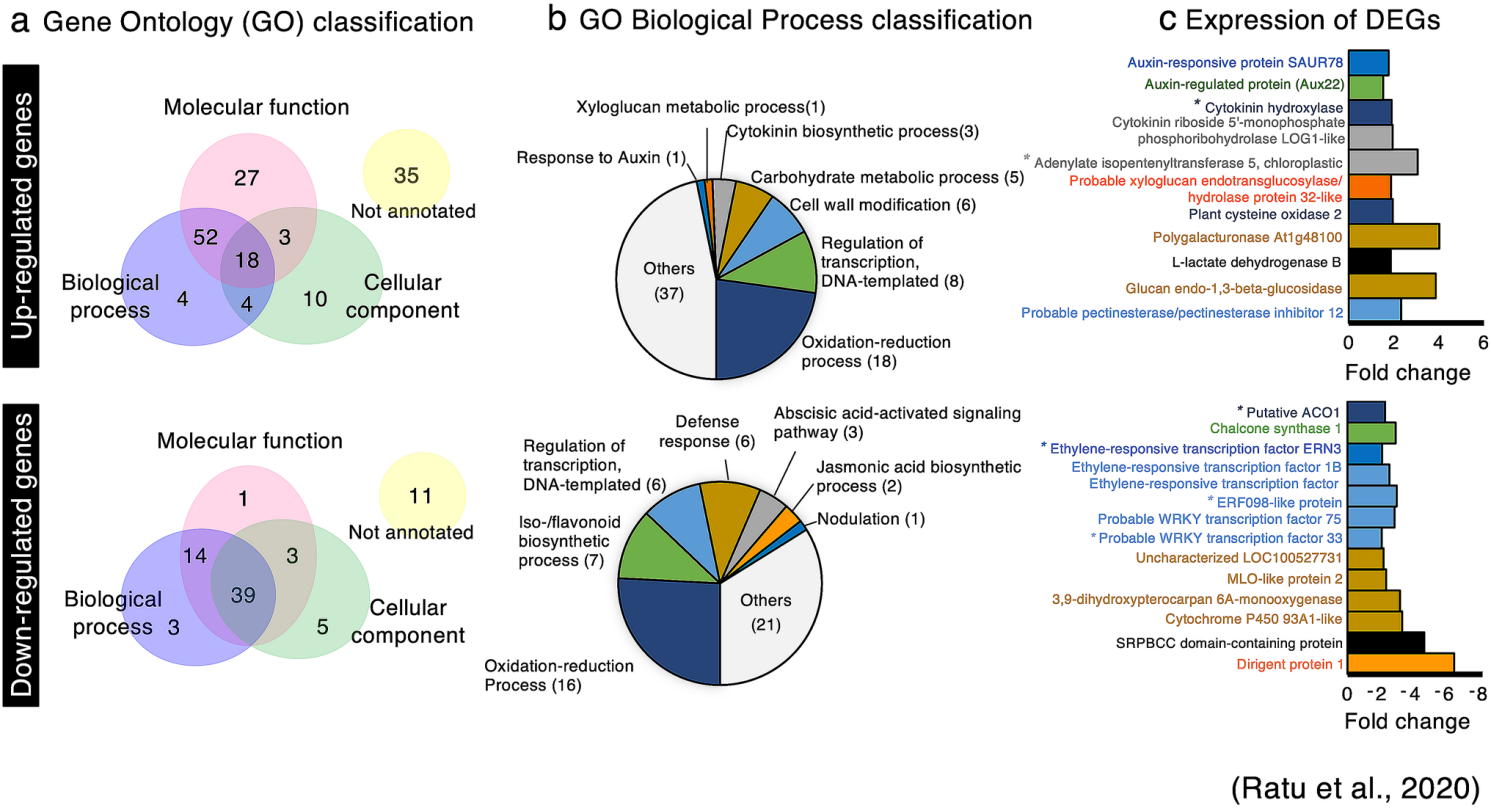
Soybean genes differentially expressed by the *B. elkanii* T3 effector Bel2-5. (**a**) Gene ontology classification of the soybean genes up- or down-regulated during interaction with *B. elkanii* wild-type as compared with *bel2*-5 deletion mutant. To detect the soybean genes that were up-regulated and down-regulated, RNAseq fold in the roots of En1282 inoculated with USDA61 wild-type were compared to the roots of En1282 inoculated with *bel2-5* deletion mutant. The soybean genes with signal intensities ≥ 1.5-fold and ≤ 0.5-fold were considered to be up-regulated and down-regulated, respectively. (**b**) GO classification on biological process (GO_BP) of soybean DEGs. Several DEGs were annotated into multiple classes of GO_BP. (**c**) Characteristics of several down-regulated and up-regulated of soybean genes. The bar’s color represents in where gene was annotated in GO_BP. The bar with black color represents gene was annotated into multiple classes of GO_BP. See Supplementary Table 3 and 4 for the details (**b**,**c**). Asterisk (*) means representative DEGs were validated with qRT-PCR (Supplementary Fig. 2).

We previously reported that the soybean nodulin genes were upregulated by the USDA61 T3SS in the absence of NF signalling^17^. To enhance our understanding of the soybean genes whose expression is modulated by Bel2-5, we performed RNA sequencing (RNA-seq) and compared the transcriptome of En1282 roots inoculated with WT and *bel2-5* deletion mutant strains (Fig. 6). At least 153 and 76 genes were found to be upregulated (fold change ≥ 1.5) and downregulated (fold change ≤ 0.5), respectively, in soybean roots during interaction with WT *B. elkanii* compared with those of genes in soybean roots during interaction with the *bel2-5* mutant. These differentially expressed genes (DEGs) were sorted using the gene-function classification system Gene Ontology (GO) for biological process, molecular function, and cellular components (Fig. 6a). Some representative DEGs were also validated by qRT-PCR analysis (Supplementary Fig. 2).

The biological processes (BPs) associated with upregulated DEGs were carbohydrate metabolic process, cell wall modification, and xyloglucan metabolic process (Fig. 6b). Genes belonging to these groups encode pectinesterase-family enzymes, β-1,3-endoglucanase, exoglucanase/xylanase-like protein, polygalacturonase, and L-lactate dehydrogenase-like protein (Fig. 6c; Supplementary Table 2). The upregulation of these genes is probably related to *B. elkanii* infection, which requires root cell wall loosening necessary for bacterial internalization. Of interest in the identified upregulated genes is the small number of soybean root DEGs annotated in the cytokinin biosynthetic process, including 2 homologues of adenylate isopentenyltransferase 5 (*IPT5*) and cytokinin riboside 5ʹ-monophosphate phosphoribohydrolase (*LOG1-like*) (Fig. 6b,c; Supplementary Table 2). One gene homologous to cytokinin hydroxylase (*CYP735A*), annotated in the BP oxidation–reduction process (Fig. 6c; Supplementary Table 2), was also upregulated during USDA61 infection. The *CYP735A* gene converts isopentenyl adenine (iP) into trans-zeatin (tZ) nucleotides in the cytokinin synthesis pathway^42^. The positive role of sufficient cytokinin levels in nodule organogenesis of soybean has been well documented^43,44^. It is therefore tempting to assume that Bel2-5 enhances nodulation in *nfr1*-mutant soybean roots by upregulating genes related to cytokinin biosynthesis.

Conversely, several downregulated DEGs were linked to iso-/flavonoid biosynthetic processes (6 genes homologous to chalcone synthase (*CHS*)), and one gene is homologous to 2-hydroxyisoflavanone synthase (Fig. 6b,c; Supplementary Table 3). In plants, the expression of these genes is involved in the salicylic acid defence response against bacterial or fungal infection^45^. Six soybean DEGs were annotated as defence response genes, including two genes homologous to SRPBCC domain-containing protein, two genes homologous to cytochrome P450 93A1-like (*CYP93A1*), one gene homologous to MLO-like protein, and one uncharacterized gene (LOC100527731) (Fig. 6b,c; Supplementary Table 3). The SRPBCC domain is a lipid/sterol-binding domain that binds various ligands via a hydrophobic pocket and may interact with salicylic acid (SA) pathway to regulate defense response^46,47^. Likewise, *MLO*-like gene belonging to plant-specific calmodulin-binding protein regulates cell death and defense response against powdery mildew. At least 20 genes encoding MLOs have been identified in soybean plant^48–50^.

We also found the downregulation of some transcription factors known to be involved in plant immunity responses against pathogen attack and stress conditions, including two homologues of putative WRKY transcription factors (WRKY33/75) and ethylene-responsive factors, *ERF1b* and *ERF98* (Fig. 6c; Supplementary Table 3). Three homologues of the aminocyclopropane-1-carboxylate oxidase (*ACO*) gene (annotated in the oxidation–reduction process) were also downregulated (Fig. 6c; Supplementary Table 3). The *ACO* gene is involved in the biosynthesis of ethylene, a plant hormone that regulates many developmental and physiological processes, including defence responses^51^. Collectively, these results suggest that USDA61 also enhances symbiosis with *nfr1* mutant soybean through dampening of host immunity to promote rhizobial infection in the early stage of the interaction. Moreover, one gene homologous to ERF required for nodulation (*ERN3*) was downregulated during USDA61 infection (Fig. 6c; Supplementary Table 3). ERN3 negatively regulates nodulation by acting as a putative repressor of the activator of NF-box-containing targets ERN1/ERN2 essential for *Medicago truncatula* (*Mt*) *ENOD11* expression^52^. This result suggests that *ERN3* downregulation might also promote the activation of nodulation-related genes on *nfr1* mutant soybean.

## Discussion

The *Bradyrhizobium* sp. ORS3257, due to its T3SS, can nodulate *Aeschynomene* plants in an NF-independent manner^18,19^. Recently, it was reported that this symbiosis relies on a set of effectors, including NopM1, NopP1, NopAB, and NopT, that play complementary and synergetic roles together with ErnA, which acts as the major effector triggering nodulation^19^. Here, we showed that during the T3SS-dependent symbiosis between *Bradyrhizobium* USDA61 and *nfr1* soybean plants, another set of effectors is required, including ErnA, NopL, Bel2-5 and possibly other effectors not yet identified. Besides, we can not exclude the possibility that *B. elkanii* NFs also play roles in the nodulation of *nfr1* soybean. We previously showed that *B. elkanii nodC* mutant induced fewer nodules than WT *B. elkanii*^17^. In addition, it has been shown that *nfr1* mutant soybeans retained the ability to suppress microbe-associated molecular pattern (MAMP)-triggered immunity upon addition of NF^53^. These results suggest that cocktail of effectors and NFs in *B. elkanii* harmoniously promote nodulation on *nfr1* mutant soybean*.*

NopL has been reported to promote nodulation in some rhizobium-legume symbioses. NopL has several phosphorylated sites, and its phosphorylation in plant cells interferes with the MAPK signalling pathway, suppressing the defence response^13,54,55^. The ErnA protein was shown to be a remarkable effector that has a direct function to induce cell division to initiate nodule organogenesis. Transgenic *Aeschynomene* lines overexpressing *ernA* form root or nodule-like structures all along the root^19^. However, during the symbiotic interaction between the USDA61 strain and soybean plants, the dominant effector is not ErnA, which is at the centre of this symbiosis, but the effector Bel2-5, which was identified as the key factor inducing nodulation on *nfr1* mutant soybean (Fig. 1).

Homology searches identified sequence similarities between Bel2-5, the XopD of *Xanthomonas* spp. and NopD of *Bradyrhizobium* sp. XS1150 (Fig. 2). The XopD effector was originally identified from *Xcv.*, which causes spot disease on tomato and pepper plants, while the NopD restricts nodulation of *Tephrosia vogelii* by *Bradyrhizobium* sp. XS1150^31,56^. In the present study, we showed that the Bel2-5 positively regulates the activation of soybean nodulation signalling. Bel2-5 and XopD share a conserved ULP1-like domain in their C-terminal region but show no homology within the N-terminal and central regions. The XopD N-terminus contains a DNA-binding domain, while the Bel2-5 N-terminus comprises a repeat sequence that is conserved only among rhizobia and an additional central region of 85 aa that is conserved in the ErnA effector (Fig. 2b; Supplementary Data 1b and 2b). These differences between Bel2-5 and XopD could underlie their distinct host specificities and target substrates, which lead to different phenotypes (symbiosis or pathogenesis) during interaction with their host plants.

A striking similarity between Bel2-5, NopD, and XopD is the fact that defective on ULP1 domain impairs their interaction with host plants. The respective mutations of this domain abrogated the capacity of USDA61 to form nodules (Fig. 4), while it abolished the ability of *X. campestris* and *Bradyrhizobium* sp. XS1150 to elicit leaf necrosis in *N. benthamiana*^31,35^. The members of the ULP1 protease family were reported to modulate diverse functions of various molecular processes, including gene expression, chromatin remodelling, subcellular localization, and genome maintenance^57^. Soybean harbours at least 13 SUMO proteases, among which *GmB2d* and *GmESD4a/b/c* are highly expressed inside root nodules^58^, however, the involvement of SUMO proteases in nodulation remains unclear. Meanwhile, *Xcv.* XopD has been reported to perturb SUMO pathway regulation for pathogenic purposes^29,59^*.* In particular, the deSUMOylation of the tomato transcription factor SIERF4 by XopD permits the suppression of ethylene production and promotes *Xcv.* infection in tomato plants^59^. Interestingly, our RNA-seq analysis showed that the expression of the soybean *ACO* gene, which catalyses the final step in ethylene biosynthesis, was repressed by USDA61, but not by the *bel2-5* mutant (Fig. 6c; Supplementary Table 3). Two ethylene transcriptional regulators, ERF1b and ERF98, together with probable TF *WRKY33*/*75*, that govern plant defence responses were also suppressed (Fig. 6c; Supplementary Table 3). Hence, it is tempting to speculate that similar to XopD, Bel2-5 may promote USDA61 infection by interfering host signalling, via ethylene-related genes or host-dependent immune system. Further study of identification Bel2-5 host targets and biochemical analysis would not only reveal the molecular basis of how Bel2-5 promotes nodulation by modulating the host SUMO system but also clarify the missing link between the SUMO system and nodulation.

Finally, we observed an overexpression of cytokinin biosynthetic genes in plants inoculated with the WT strain, in contrast to the situation in those inoculated with the *bel2-5* mutant (Fig. 6b,c; Supplementary Table 2). It is tempting to speculate that transcriptional changes in cytokinin biosynthesis genes influence the downstream targets of NF perception, *ENOD40* and *NIN*, to stimulate nodule development. *ENOD40* has been shown to be induced in the modest mode, whereas *NIN* expression was increased at the steady-state level in *Lotus japonicus* roots by external application of cytokinin^60^. Likewise, spontaneous nodule-like structure formation was triggered by overexpression of the cytokinin complete gene set, *LjIPT3*, *Cyp735a*, and *LjLog4*^61^. Although the molecular mechanism underlying the induction of cytokinin biosynthetic related genes remains unclear, this effect might be related to the N-terminus of Bel2-5, which has little similarity with XopD (Fig. 2b), considering that XopD does not have this activity. Further analysis of N-terminal repeat domains that are well conserved among only rhizobia could provide insight into the unique symbiotic function of Bel2-5 and how they differentiate Bel2-5 from other pathogenic homologues.

**Figure 7:**
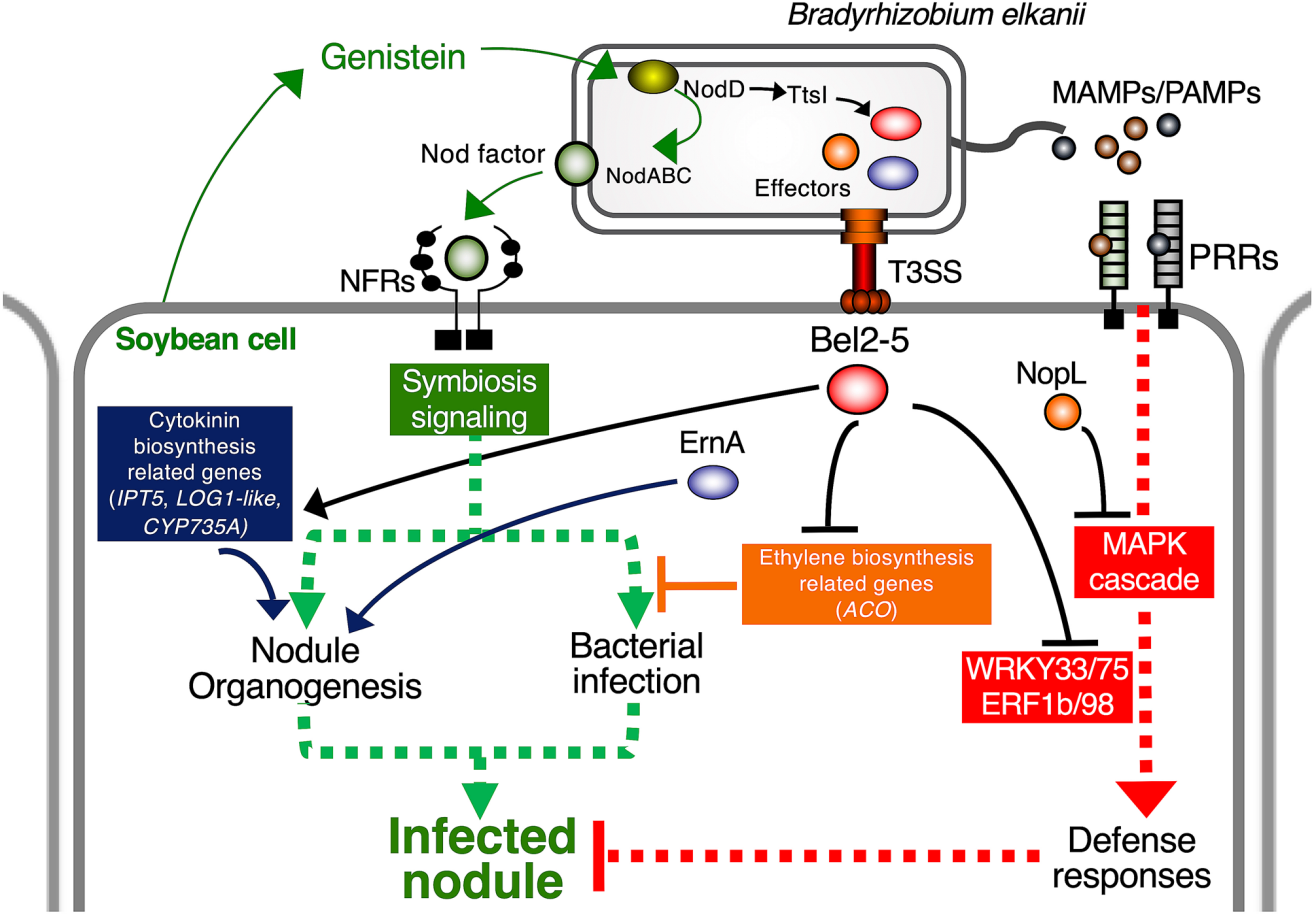
Proposed model for nodulation signaling and modulation by *Bradryhizobium elkanii* type III effector Bel2-5. A soybean root-derived genistein induces the production of Nod factors (NFs) in *B. elkanii*. Recognition of NFs by NF receptors triggers a signaling cascade leading to nodulation. The genistein also induces *B. elkanii* to inject effector proteins into soybean cells. NopL suppress plant defense responses by modulating the MAPK cascade. The ErnA effector triggers nodule organogenesis by a yet unknown mechanism. Bel2-5 effector likely interferes with the nodulation signaling pathway at least at two levels: (1) by modulating cytokinin biosynthesis-related genes that promoting nodule organogenesis and (2) by repressing ethylene biosynthesis-related genes and host-dependent defence responses that are deleterious to the bacterial infection. It should be noted that these DEGs may contain secondary and tertiary indirect effects as well as Bel2-5 direct effects.

In conclusion, our study identified that the USDA61 Bel2-5 effector enhances soybean nodule formation by activating soybean nodulation signalling. We propose a model of modulated by USDA61 T3Es in Fig. 7. The Bel2-5 effector likely interferes with the NF-dependent signalling pathway at least at two levels: (1) by modulating cytokinin biosynthesis-related genes that favouring nodule organogenesis, and (2) by repressing ethylene biosynthesis-related genes and host-dependent defence responses that are deleterious to the rhizobial infection. It should be noted that these DEGs may contain secondary and tertiary indirect effects as well as Bel2-5 direct effects. Two other effectors, ErnA and NopL, might be required for efficient and robust nodule development. Identification of the host targeted protein(s) and characterization of the Bel2-5 unique domains would provide insights into how rhizobia have employed and customized T3Es for symbiotic purposes during co-evolution between plants and bacteria.

## Materials and methods

### Bacterial strains and growth conditions

The bacterial strains and plasmids we used are listed in Supplementary Table 4. *B. elkanii* strains and *A. tumefaciens* GV3101 were grown at 28 °C in Arabinose-Gluconate (AG) medium^62^ supplemented with appropriate antibiotics (50 µg/mL polymyxin, 200 µg/mL kanamycin, 200 µg/mL streptomycin, and 100 or 200 µg/mL spectinomycin). *Escherichia coli* strains were grown at 37 °C in Luria–Bertani medium^63^ supplemented with appropriate antibiotics (50 µg/mL kanamycin, 50 µg/mL streptomycin, and 10 µg/mL tetracycline). All conjugation processes were performed on Peptone Salt Yeast Extract (PSY) medium^64^.

### Antibodies

To obtain anti-NopA and anti-Bel2-5 antisera, a rabbit was immunized with a synthesized peptide corresponding to a partial sequence of NopA (N-RSMLLRTVTTELQTTKKAADERVQ-C) or Bel2-5 (N-EALRSGNAAERTAK-C and N-DAAELRPAKRPRTL-C), which were coupled to carrier proteins prior to immunization, according to the manufacturer’s protocols (Eurofins Japan, Tokyo, Japan).

### Plasmid construction, mutagenesis, and complementation

All constructions made in this study are listed in Supplementary Table 4, which includes the primers and cloning strategies. For the construction of insertional mutants (obtained by single crossing-over), a 350- to 600-bp internal fragment of the target gene was amplified by PCR and cloned into the nonreplicative plasmid pSUPSCAKm. For the construction of in-frame deletion mutant of *bel2-5* (∆*bel2-5*), 800- to 900-bp PCR fragments corresponding to the upstream and downstream flanking regions of the *bel2-5* were merged by overlap extension PCR and cloned into pK18mobsacB. The deletion mutant was selected on AG medium supplemented with 10% sucrose. Sucrose-resistant clones were checked for loss of kanamycin resistance from the pK18mobsacB plasmid, and kanamycin-sensitive clones were screened by PCR for the deletion of the *bel2-5*.

For *bel2-5* complementation, ~ 4.5-kb DNA fragments containing *bel2-5* with its promoter sequence were PCR amplified and cloned into pBjGroEL4::DsRed2. The constructed plasmid (pBjGroEL4::*bel2-5*) was mobilized into the ∆*bel2-5* by conjugation. Integration of the *bel2-5* gene into the chromosome of ∆*bel2-5* was confirmed by antibiotic resistance, PCR and sequencing.

The substitution of Bel2-5 ULP1 putative catalytic residues (H/D/C) were performed using a QuickChange Lightning Site-Directed Mutagenesis Kit (Agilent Technologies). First, a partial fragment of *bel2-5* (1474 bp) from the 3ʹ-end (C-terminus) was amplified by PCR and cloned into the plasmid pS18mob to generate pS18mob-*bel2-5*ULP1, which was then used as the template for generating plasmid carrying a substitution of H/D/C into alanine using specific primer sets. The constructed plasmids were confirmed using sequencing analysis and then integrated into WT USDA61 through single homologous recombination. The constructed mutants were screened for antibiotic resistance and confirmed using sequencing analysis.

### Nodulation tests

Soybean seeds (En1282^65^ and Enrei) were surface-sterilized and germinated as described^20^; 2-days-old germinated seedlings were transferred into a plant box (CUL-JAR300; Iwaki, Japan) containing sterilized vermiculate and inoculated with 1 × 10^7^ cells/mL *B. elkanii* strains or sterilized water (mock treatment). Plants were cultivated in a plant growth chamber at 25 °C and 70% humidity under a 16/8-h day/night regimen; appropriate amounts of B&D nitrogen-free solutions^66^ were added for watering the plants. Nodule number, nodule weight, and plant fresh weight were measured at 30 dai.

### Bacterial RNA extraction and expression analysis

RNA was extracted from bacterial cells as described^14^. Briefly, rhizobial strains were grown at 28 °C in AG medium supplemented with appropriate antibiotics (50 µg/mL polymyxin and 200 µg/mL streptomycin), and after 3 days, bacterial cells (OD_600_ = 0.4) were transferred to new medium supplemented with or without 10 µM genistein. After 48 h of growth, bacterial cells were harvested for total RNA extraction using TRI Reagent (Cosmo Bio Co., Ltd.). Total bacterial RNA was treated with Recombinant DNase I (RNase-free) (Takara Bio Co., Ltd.) and purified using phenol–chloroform extraction.

The extracted RNA samples were used as the template for cDNA synthesis in a final volume of 20 µL (containing 400 ng of total RNA) by using the SuperScript III First-Strand Synthesis System for RT-PCR (Invitrogen). The RT-qPCR mixture comprised of 1 µl of tenfold diluted cDNA template, 1 µl of primer solution (containing 2 µM of each forward and reverse primers), 6.6 µl of Milli-Q water and 10 µl of 2 × KAPA SYBR Fast qPCR Master Mix (KAPA Biosystems). Thermal cycling condition consisted of 2 m in at 50 °C, 30 s at 95 °C, and 40 cycles of 5 s at 95 °C and 30 s at 60 °C^16^. RT-qPCR was analyzed using a StepOne Real-Time PCR System (Applied Biosystems) with the primer set listed in Supplementary Table 4. Each sample consisted of three biological replicates and two technical replicates. Transcript levels of *bel2-5* were normalized to those of the housekeeping gene *atpD*^67^, which were measured in the same samples.

### Purification and analysis of extracellular proteins

For isolation of extracellular proteins, AG medium was inoculated with 1:100 dilution from a *B. elkanii* preculture. Extracellular proteins of *B. elkanii* strains were recovered from 500 mL of cultures (+ /− 10 µM genistein) grown on an incubator shaker (200 rpm) for 48 h. Cultures were centrifuged in two steps (1 h, 4000×*g*, 4 °C, and then 30 min, 8000×*g*, 4 °C), the obtained supernatants were lyophilized to complete dryness, and extracellular proteins were isolated as described^68^. For Bel2-5 and NopA immunodetection, proteins were separated on 4–15% SDS-PAGE gradient gels, electroblotted onto Polyvinylidene Difluoride Membranes (Bio-Rad, USA), blocked with Western Blot Blocking Buffer (Fish Gelatine; Takara, Japan), and incubated with a raised antiserum as the primary antibody (Bel2-5 = 1:1000 or NopA = 1:4000 dilution) and then with a horseradish peroxidase-conjugated goat anti-rabbit immunoglobulin G antiserum (secondary antibody, 1:40,000). An ECL Prime Kit (GE Healthcare, UK) was used to develop the membranes, and the Bel2-5 band was detected using a LAS-3000 Luminescent Image Analyzer (Fujifilm, Japan).

### Adenylate cyclase (Cya) assay

A *bel2-5cya* fusion was constructed by cloning the C-terminal-coding region of *bel2-5* without the stop codon (~ 575 bp); the fragment was PCR amplified and cloned into the plasmid pSLC5Sm^14^ to generate the plasmid pSLC5Sm-*bel2-5cya*, which was confirmed through sequencing analysis. Integration of the *bel2-5cya* fusion into WT USDA61 and its derivative BErhcJ mutant was screened based on antibiotic resistance and confirmed using PCR.

The cAMP levels were quantified from *G. max* cv. Enrei root nodules collected randomly from three separate plants at 18 dpi. The collected root nodules were frozen in liquid nitrogen and ground into a fine powder, which was resuspended in a 10 × volume of 0.1 M hydrochloric acid solution (per nodule weight). The suspension was centrifuged, and the supernatant was used for cAMP measurement by using a Cyclic AMP (Direct) Enzyme Immunoassay (EIA) Kit (Cayman Chemical Co., Ann Arbor, MI, USA) according to the manufacturer’s instructions. Each sample was diluted for measurement of the cAMP concentration in the detection range of the standard assay.

### Subnuclear localization analysis

The ORF of *bel2-5* without the stop codon was PCR amplified and cloned into the Gateway Entry Vector pCR8/GW/TOPO (Thermo Fisher). The PCR product was recombined downstream of a 35S promoter into the destination vector pB7FWG2.0 (https://gateway.psb.ugent.be) with a C-terminal GFP tag. The recombinant vector was transformed into competent cells of *A. tumefaciens* strain GV3101. Leaves from four-week-old *N. benthamiana* plants were infiltrated using a needleless syringe containing bacteria resuspended in infiltration buffer (10 mM MgCl2; 10 mM MES-KOH pH 5.6; 150 µM acetosyringone) and adjusted to OD600 = 0.5.

At 48 h following *A. tumefaciens* infiltration, *N. benthamiana* leaf samples were incubated in 5 μg/mL DAPI solution (4′,6-diamidino-2-phenylindole; Sigma) for 30 min. Localization of fluorescently labelled *bel2-5* was observed with a confocal microscope (CarlZeiss LSM700). GFP was excited at 488 nm, with emission signal collection at 490–530 nm, while DAPI was excited at 405 nm, with emission signal collection at 410–470 nm. Images were obtained using ZEN2008 software (Zeiss).

### Soybean RNA-Seq and qRT-PCR analysis

#### Sample preparation and cDNA library construction

For RNA extraction from soybean roots, seeds were surface-sterilized, germinated at 25 °C for 2 days, and transferred to a DIK-710A Seed Pack (Daiki Rika Co., Ltd.) with B&D nitrogen-free solution. On the following day (the third day of germination), seeds were inoculated with 2 × 10^7^ cells/mL bacterial cultures (3 replicates of seeds for each sample). Then, three days after inoculation, the soybean *nfr1* mutant roots were immediately frozen in liquid nitrogen and then ground into a fine powder; 100 mg of the powder was measured and used for total RNA extraction using RNeasy Plant Mini Kit (Qiagen) and treated with DNase I (Qiagen) according to the manufacturer’s instructions. RNA quality and concentration were evaluated using a NanoDrop 2000/200c (Thermo Fisher). The measured A260/280 and A260/230 ratios were ~ 2.1 and ~ 2.1 to 2.4, respectively, in the case of all samples. The cDNA library was constructed according to the True Stranded mRNA Sample Preparation Guide, Part #15031047 Rev. E Protocol by using an LT Sample Prep Kit (Illumina).

#### Read trimming and mapping to the reference genome

The ‘raw reads’ obtained were filtered to remove low-quality and contaminant reads, including adaptor sequences, DNA, or PCR duplicates, which generated trimmed reads. The trimmed reads were mapped to the reference genome Glycine_max_v2.1 by using HISAT2 version 2.1.0 and Bowtie2 2.3.4.1 software (https://ccb.jhu.edu/software/hisat2/index.shtml). The aligned reads were transcribed using StringTie version 1.3.4d (https://ccb.jhu.edu/software/stringtie/), and expression profiles were normalized to FPKM (fragments per kilobase of transcript per million mapped reads) or RPKM (reads per kilobase of transcript per million mapped reads) values.

#### Screening of DEGs and enrichment analysis

Bel2-5-dependent DEGs were defined using the following criteria: *p* < 0.05; fold change ≥ 1.5 as upregulated, and fold change ≤ 0.05 as downregulated. The classified genes were examined for enrichment and annotation using Blast2GO software^69^ and QuickGO (https://www.ebi.ac.uk/QuickGO/).

#### qRT-PCR analysis for selected soybean DEGs

For qRT-PCR of selected soybean DEGs, including three defense- and symbiosis-related genes were measured using the same protocol with Bel2-5 expression quantification described previously^16^. The primer set used in qRT-PCR are listed in Supplementary Table 4. Transcript levels of selected soybean DEGs was normalized to the expression of its housekeeping gene *SUBI-2*^70^ measured in the same samples.

### Microscopy

For microscopy analysis, nodules were sectioned using a microtome (VT1000s; Leica Biosystem, Germany) and examined using an SZX9 Microscope (Olympus, Japan).

### Bioinformatics and statistical analysis

NLSs were predicted using NLS Mapper (http://nls-mapper.iab.keio.ac.jp/cgi-bin/NLS_Mapper_form.cgi). ULPs domain were inspected using NCBI Conserved Domain Search (https://www.ncbi.nlm.nih.gov/Structure/cdd/wrpsb.cgi). The Bel2-5 repeat domain was analysed using Tandem Repeats Finder^71^ (https://tandem.bu.edu/trf/trf.html). Homology searches were performed using BLASTP from the National Center for Biotechnology Information (NCBI) (https://blast.ncbi.nlm.nih.gov/) and MEROPS from the Peptidase Database (https://www.ebi.ac.uk/merops/submit_searches.shtml). Bel2-5 and its homologues were aligned using the MUSCLE or CLUSTALW algorithm. The phylogenetic tree was constructed by using MEGA 7 software with the neighbour-joining method and 1000 bootstrap replicates^72^. Data were analysed using Student’s *t*-test and Tukey’s Honestly Significant Difference (HSD) test, performed using IBM SPSS Statistics 22.0 software.

## Supplementary Information

Supplementary Information.

## Data Availability

The RNA-seq data discussed in this publication have been deposited in DDBJ’s Sequence Read Archive (DRA) (https://www.ddbj.nig.ac.jp/dra/index-e.html) repository and are accessible through GEO Series Accession Number DRA010122.
