# Supplementary material for: Rhizobia use a pathogenic-like effector to hijack leguminous nodulation signalling
Source: Sci Rep. 2021 Jan 21;11:2034. doi: 10.1038/s41598-021-81598-6 (PMC7820406; doi:10.1038/s41598-021-81598-6)
Supplement: Supplementary file 1 — Supplementary Information. [file 41598_2021_81598_MOESM1_ESM.docx]

**Supplementary Data**

**Rhizobia use a pathogenic-like effector to hijack leguminous nodulation signalling**

Safirah Tasa Nerves Ratu^1^, Albin Teulet^2^, Hiroki Miwa^3^, Sachiko Masuda^3^, Hien P. Nguyen^1^, Michiko Yasuda^3^, Shusei Sato^4^, Takakazu Kaneko^5^, Tsuneo Hakoyama^6^, Makoto Hayashi^6^, Eric Giraud^2^, and Shin Okazaki^1,3^*

***Corresponding author**

Graduate School of Agriculture, Tokyo University of Agriculture and Technology, Saiwai-cho 3-5-8, Fuchu, Tokyo 183-8509, Japan

Phone/Fax: +81-42-367-5847

E-mail: sokazaki@cc.tuat.ac.jp

**Supplementary Material and Method**

1. **Quality control processes for RNA samples used for RNAseq analysis**
2. **RNA Concentration and Purity**

Table 1. Concentration of total RNA extracted from soybean roots and the RNA purity

| **Inoculated with** | **Concentration (ng/µL)** | **A260/280** | **A260/230** |
| --- | --- | --- | --- |
| Mock – 1 (C1) | 821.1 | 2.12 | 2.06 |
| Mock – 2 (C2) | 1158.8 | 2.10 | 2.28 |
| Mock – 3 (C3) | 977.1 | 2.07 | 2.27 |
| USDA61 – 1 (W1) | 695.5 | 2.10 | 2.32 |
| USDA61 – 1 (W2) | 1625.7 | 2.13 | 2.02 |
| USDA61 – 1 (W3) | 1282.2 | 2.12 | 2.36 |
| Δ*bel2-5* (M1) | 901.4 | 2.12 | 2.19 |
| Δ*bel2-5* (M2) | 842.7 | 2.10 | 2.22 |
| Δ*bel2-5* (M3) | 672.7 | 2.11 | 2.38 |

1. **Raw Data Statistics**
   - Analyze the quality control of the sequence raw reads, including overall reads quality, total bases, total reads, GC (%) and basis statistics are calculated. In order to reduce biases in analysis, artifacts such as low quality reads, adaptor sequence, contaminant DNA, or PCR duplicates are removed.
   - The total number of bases, reads, GC (%), Q20 (%), Q30 (%) are calculated for 9 samples.

Table 2. Raw data statistic

| **Sample ID** | **Total read bases** * | **Total reads** | **GC (%)** | **Q20 (%)** | **Q30 (%)** |
| --- | --- | --- | --- | --- | --- |
| Mock – 1 (C1) | 4,788,776,024 | 47,413,624 | 44.76 | 98.17 | 94.80 |
| Mock – 2 (C2) | 4,008,897,454 | 39,692,054 | 44.93 | 98.27 | 95.01 |
| Mock – 3 (C3) | 4,346,266,340 | 43,032,340 | 44.80 | 98.03 | 94.44 |
| USDA61 – 1 (W1) | 4,830,677,288 | 47,828,488 | 44.59 | 98.62 | 95.78 |
| USDA61 – 1 (W2) | 5,757,880,114 | 57,008,714 | 44.71 | 98.16 | 94.81 |
| USDA61 – 1 (W3) | 4,174,163,148 | 41,328,348 | 44.92 | 98.06 | 94.54 |
| Δ*bel2-5* (M1) | 5,074,582,390 | 50,243,390 | 44.84 | 98.68 | 95.88 |
| Δ*bel2-5* (M2) | 4,430,655,678 | 43,867,878 | 45.01 | 98.22 | 94.90 |
| Δ*bel2-5* (M3) | 5,131,658,702 | 50,808,502 | 44.86 | 98.23 | 94.93 |

(* Total read bases = Total reads x Read length)

Total read bases: Total number of bases sequenced

Total reads: Total number of reads

GC (%): GC content

Q20 (%): Ratio of bases that have phred quality score greater than or equal to 20

Q30 (%): Ratio of bases that have phred quality score greater than or equal to 30

1. **Average Base Quality at Each Cycle**

- The quality of produced data is determined by the phred quality score at each cycle. Box plot containing the average quality at each cycle is created with FastQC (<http://www.bioinformatics.babraham.ac.uk/projects/fastqc>).
- The x-axis shows number of cycles and y-axis shows phred quality score. Phred quality score 20 means 99% accuracy and reads over score of 20 are accepted as good quality.

1. **Trimming Data Statistics**

- To remove adapter sequences and bases with base quality lower than three from the ends the Trimmomatic programs (<http://www.usadellab.org/cms/?page=trimmomatic>) is used.
- The bases of reads that does not qualify for window size 4, and mean quality 15 are trimmed using sliding window method.
- Reads with length shorter than 36bp are dropped to produce trimmed data
- The quality of trimmed data is determined by the phred quality score at each cycle as mentioned previously.

Table 3. Trimming data statistic

| **Sample ID** | **Total read bases** * | **Total reads** | **GC (%)** | **Q20 (%)** | **Q30 (%)** |
| --- | --- | --- | --- | --- | --- |
| Mock – 1 (C1) | 4,667,245,928 | 46,529,922 | 44.75 | 98.74 | 95.63 |
| Mock – 2 (C2) | 3,915,485,412 | 39,038,700 | 44.91 | 98.79 | 95.76 |
| Mock – 3 (C3) | 4,232,822,199 | 42,201,142 | 44.79 | 98.62 | 95.31 |
| USDA61 – 1 (W1) | 4,755,119,997 | 47,304,850 | 44.58 | 99.01 | 96.35 |
| USDA61 – 1 (W2) | 5,614,626,947 | 55,961,054 | 44.69 | 98.76 | 95.67 |
| USDA61 – 1 (W3) | 4,059,240,504 | 40,512,650 | 44.90 | 98.66 | 95.42 |
| Δ*bel2-5* (M1) | 4,982,450,239 | 49,724,602 | 44.81 | 99.03 | 95.38 |
| Δ*bel2-5* (M2) | 4,328,548,524 | 43,128,552 | 45.00 | 98.75 | 95.66 |
| Δ*bel2-5* (M3) | 5,008,778,618 | 49,909,104 | 44.85 | 98.78 | 95.73 |

(* Total read bases = Total reads x Read length)

Total read bases: Total number of bases sequenced

Total reads: Total number of reads

GC (%): GC content

Q20 (%): Ratio of bases that have phred quality score greater than or equal to 20

Q30 (%): Ratio of bases that have phred quality score greater than or equal to 30

1. **Mapping Data Statistics**

The trimmed reads are mapped to references genome with HISAT2, splice-aware aligner (https://ccb.jhu.edu/software/hisat2/index.shtml).

Table 4. Mapped data statistics

| **Sample ID** | **# of processed reads** | **# of mapped reads (%)** | **# of unmapped reads (%)** | |
| --- | --- | --- | --- | --- |
| Mock – 1 (C1) | 46,529,922 | 44,740,502 (96.15%) | | 1,789,420 (3.85%) |
| Mock – 2 (C2) | 39,038,700 | 37,524,228 (96.12%) | | 1,514,472 (3.88%) |
| Mock – 3 (C3) | 42,201,142 | 40,376,864 (95.68%) | | 1,824,278 (4.32%) |
| USDA61 – 1 (W1) | 47,304,850 | 45,180,573 (95.51%) | | 2,124,277 (4.49%) |
| USDA61 – 1 (W2) | 55,961,054 | 53,859,070 (96.24%) | | 2,101,984 (3.76%) |
| USDA61 – 1 (W3) | 40,512,650 | 38,837,262 (95.86%) | | 1,675,388 (4.14%) |
| Δ*bel2-5* (M1) | 49,724,602 | 47,576,849 (95.68%) | | 2,147,753 (4.32%) |
| Δ*bel2-5* (M2) | 43,128,552 | 41,346,814 (95.87%) | | 1,781,738 (4.13%) |
| Δ*bel2-5* (M3) | 49,909,104 | 47,874,813 (95.92%) | | 2,034,291 (4.08%) |

Processed reads: Number of cleaned reads after trimming

Mapped reads: Number of reads mapped to reference

Unmapped reads: Number of reads that failed to align

**Supplementary Data 1**

1. Nucleotide sequences of *bel2-5* in *Bradyrhizobium elkanii* USDA61 (Accession number: KT267563.1). The gene and *tts* box are shown in bold and bordered, respectively.

TTGCGTTTCGATTTTTGCCTCGCGCGTTCAGCGCATCCTGTCAAGATGTTGGCTGGAGCCGCGAAAGCTGGCCGTACGCCTCACCGATGAGAAGCAGTATTTTGTCTCTGAATCTTCGGTATCTTCGGTCTCTCGGCTGTTGGCGGCGAAGCGCGCAGGCACATGCACCATGCTGTCTTGGCGCGGCAATTTTCGTCATCAGGTTCTCGACAGCTTCCCCATTTAACAAAACATACAGGGCTGGTCGCCTTGCAAAAACATCGGCTGCCGATGCTCACGGTGCGCGCCTGTCGAGGGCCGTAAAGGGTGGTTTGTTGCTGATTCCCAGCATGTCAGATCGAAGCCCATGTTCATATTTTGAAGGTGAGAACGG**ATGGATTTCCCCTCGACCAAGTCGGTGCATGAACAATCGGACAGCACGGGCCCGCAAGAGAGCTCACCCGCGGCTCCGTCCGCAGCCGCGGCGACCTTTGAGCGGCAATTGAGCGAGATCGCCAACTCAGATGGAGGTGGTGGAGCAATGCCGGCCGCCTCGGCGCTGCAGTCGGCTCAGTCAACTTGGGTTTTGATAGGACGGCAGGGCAAGCATCTCCTTTATTCCGAGGACGCTCGCCTTATTTCGGGGCTTGAGAAGGCCCTTATCGGCAACCCCACCCACCGCAACGCTGGGGACTATCTAACTTCACTTCGCAGCTTTGGCGGCTGGCTCTTCGCAAATAACAAAACGAGCATTGTTGCTCGGCTCAACGACCCGTCGCTGACCCATGATGCACGCGAGTTCGAAAAGAGGCGTCCCTCGAATATCCTTGCGGCATTGAACCATCTCCGGACCTTCCAGTCGACGGGCGGAGTGACGGTCACAGCCCGCACTGAGCTAAATCCTCACCCTCAGGACGCGGATCTCATCAACGAGTACAAAAAAGAAACAGCGACAAGTACCGGCAGGATGTATGCAACTGCTCTTCGCAGTTTCGGTCACTACCTGCGTGAAAACAACAAGAAGGGCATTGCTACTCGGCTTTCCGGCGGGGCGTTGGATGAAGATGTCAATAGCTATAAGAAGGGCGCCGGTGCTGATTCGAGGATCGGTGCCGCTCTGGCTCAACTCCGAAAATCGCAGGCCGGCGCTAAGGCGATGGAGCCCGAGCGCCATTTTGATCCCAAAGACGCGGCCCTGATGGAGTCGATGCAGGTCGGCGACGCCGCTGCGCAGCACAGTGCGTCGCAGCAAGCTGGCAGTTGGTCAGAGGAATTGAACCATCTCCGGACCTTCCAGTCGACGGGCGGAGTGACGGTCACAGCCCGCACTGAGCTAAATCCTCACCCTCAGGACGCGGATCTCATCAACGAGTACAAAAAAGAAACAGCGACAAGTACCGGCCGGATGTATGCAACTGCTCTTCGCAGTTTCGGTCACTACCTGCGTGAAAATAACAAGAAGGGCATTGCTACTCGGCTTTCCGGCGGGGCGTTGGATGAAGATATCAATAGCTATAAGAAGGGCGCCGGTGCTGATTCGAGGATCGGTGCCGCTCTGGCTCAACTCCGAAAATCGCAGGCCGGCGCTAACGCGATGGAGCTCGAGCGGCATATTGATCCCAAAGACGCGGCCCTGATGGAGTCGATGCAGGTCGGCGACGCCGCTGCGCAGCACAGTGCGTCGCAGCAAGCTGGCAGTTGGCCAGAGGAATTTCTTCCTGCGGAAGGCCACGATCAGGATTTGGGCCGGATGGACGAACCCGGCCCGTCGTCGTCGGCGCCGCAGCCGGCTCAGTCAACTGGGATTTTGAGAGGGCGGAGGAAGCCTCTTTATTCCGAGGATGCTCCCCTTATTTCGGGGCTTGAGGAGGCCCTCCGCAGTGGCAACGCCGCCGAACGCACCGCCAAAGACCTTGTAGGCCCGCTTCGCGCCTTTGGCCGCTGGCTCTTGGCAAATAACAAAACGAGCATTGTTGATCGGCTCGAAAAAGAGTCGCTGACCGATGATGCGCGTGAGTTCATCGAAAAGGGTAAGGGCAGTCGCCTCCTTATTAGGTCAATAGGTCTTCTCCGGACCTTTCAGTCGACGGGCGGAGTGACGGTCACAGCCACCACTGAGCTAAATCCTTACCTCCAGGACGCGGCTCTCATCATAGAGTACCAAAACGAAGCAGCGACAAGTACCGGCGGTGTGTATGCGACTGCTCTTCGCAGTTTCGGTCACCACCTGCGTGAAAATAACAAGAAGGGCATTGCTACTCGGCTTTCCGGCGGGGCGTTGGATGAAGATGTCGAGGCCTATAAAAAGGACTTCGGTGGCATCCGGACGATCGATGCCGCATTAGGTCAACTCCGAAAATCACAGGCGGGCGCTAAAGCGATGGAGCGCGAGCGCTATATTTCCCCCGGTCCTGATCCCGAAGGCGCGGCACTGATGGAGCCGAGGCGGGCCGGCGACGCCGCAGCGCAGCACAGCTCGCAGGAAGTTGGCAGTTGGCCAGAGAAACTGCTTCCTGCGGAACGCCACGAGCAGGATTTGGTTTTGGGGCTGATGGACGAACCCGGCCCGTCGTCATCTCTCGAGCCAGTCGCGCGGCACGACCAGGCATCGGATCCCGGAGATTCCATTCGTCCCCTGAACTGGCGCCGGGACGGCCAGCAGTTCTCGGAAGAGCCGATGGCTGCACTTGCCAGGAGCAACCTCCCGCCAAGCGAGGAGATCCTCATCAACGATGAACAGGATGCAGCTGAGTTAAGGCCAGCGAAGAGGCCGAGGACCCTAGACAATCCGCAAGGCCTTGCTATTGAGCGGCTGCTGAGCGAGATCGCCGCGACCCCGGCCCCCACCCATCAACAGGGTGCATCGCCATGGCATGCGCAGCCGATGATGCAGGCGAGCGGGCACGAAGATGCAACGGCGCCGCATGCGGCCGCGACGTACGTCGCGGGCGCCGCCGCGCAGCACAGCGCGCCGCAGGGAGCTGTCAGTCGGCCATTGGTCCTCCCGGAAGGTTACGACCGGGATCTGCGTTTGATGGGGAAAGACGGCCCATCGTGGCCCGAGGTTCCCCCTGGGCAGGCGCAGGACATAGTCCAAGCTGGACGGCAGCAACCTGCGTGGTCCGCCTCAACCTGGTCGCCGCAGATGCCGCTCGACTTTGATTGGAGTATGTGGCCGACGCTGGAAGCAGCGCCGGCGCCGGCTGCCAGGGCTCGCTCAGGCACCTACGGCGGTCTTGAGTCATTGGTGCATCTGGATGCGCCCACGCCGTCCGAATTGCGCGACGATGCTCACTTTGCGCCGGCGCCCGCTGCCAGGGCTCGCTCAGGCACCTACGGCGGTCTTGAGTCATTGGTGCATCTGGATGCGCCCACGCCGTCCGAATTGCGCGACGATGCTCACTTTGCGCCGGCGCCCGCTGCCAGGGCTCGCTCAGACACCTACGGCGGTCTTGAGTCATTGGTGCATCTGGATGCGCCCACGCCGTCCGAATTACGCGACGATGCTCACTTTGCGCCGGCGCCCGCTGCCAGGGCTCGCTCAGACACCTACGGCGGTCTTGAGTCATTGGTGCATCTGGATGCGCCCACGCCGTCCGAGTTGCGCGACGATGCTCACTTTGCGGCGGCGCCTTTTGCCAGGGCTCGCTCAGACGCCTACCGCGGTTTTCCATTGGTCGATCTGACTGCGCCCACGCCGTCCGAATCACGTGACGATGCTAATTCTGTACGCCCGTTTCCGAGCACCTCCGCTAATGCTCAGATCGGGGCTTTAGATCCGACAGTCTCGTCTCACGGCCACGGGCTGGTGCTCGATGACACAGAATGGCTGGGCGACCAGCATATCGACAGGGATTACGGGCTCCAGGAGCAGGATTTGCAGAGGAACGATCCGGATCTCGCCGCCCGGACGCGGTTCGTGAATCCCCTCATCGCCCTAAATTATCTGCGCTCTAACGACGATGGCGTCGTGCTAACCGAGTTCCAGCGCATCGTCTATGACGATAATGGTAATGATACAGCCGACTTCCTGTTCCTGCCCGTGATTAATGGCAATCCTGAAGATCCTAATAGCCGCGGCAACCATTGGTCGCTGCTGTTCGTAGATCGCAGCGACCGGTGGCGGCCGGTCGCCTATCACTACGATTCCTACGGCGGACTCAACAACAGAGATGCAGCACATCTCGCAAGAAGGCTGAACCTCCCCCTGGAGCTAGCCGACATGGCCCAGCAGCAGAACACTTATGATTGCGGCGTCTTTGTCGTGGACGGCACGCGGGAGCTGGTTAGGCAATTGGCGCAAGGATGGGAGCCAGACCAGCTGAACCTTAGCAACGTCGTTGCCAATCGGCAGGCGCTGCAGAACCGACTCAGGGGTTGA**

1. Amino acid sequences of Bel2-5 in *B. elkanii* USDA61 and its homologs in several selected rhizobial and pathogenic bacteria, including *B. japonicum* Is-34 (KGT79298.1), *B. diazoefficiens* USDA 110 (BAC53509.1), *Bradyrhizobium* sp. XS1150 (AWS20424.1), *Sinorhizobium fredii* HH103 (CCE98838.1), *Mesorhizobium loti* MAFF303099 (BAB52630.1), and *Xanthomonas campestris* pv. *vesicatoria* (DAA34040.1). The putative repeat domains (I and II), EAR motifs (I and II), NLS, and ULP1-like domain are shown in bordered, green, blue, and grey shading, respectively. The predicted triad catalytic core of ULP-like domain is written in red font. The Bel2-5 and its homologs repeat domains, NLS, EAR motifs (LxLxL), and ULP domain were annotated using Tandem Repeats Finder, NLS Mapper, visual inspection, and NCBI Conserved Domain Search, respectively. Except for NopD and XopD effectors were annotated based on Xiang et al.^1^ and Hotson et al; Kim et al.^2,3^, respectively.

**Bel2-5**

MDFPSTKSVHEQSDSTGPQESSPAAPSAAAATFERQLSEIANSDGGGGAMPAASALQSAQSTWVLIGRQGKHLLYSEDARLISGLEKALIGNPTHRNAGDYLTSLRSFGGWLFANNKTSIVARLNDPSLTHDAREFEKRRPSNILAALNHLRTFQSTGGVTVTARTELNPHPQDADLINEYKKETATSTGRMYATALRSFGHYLRENNKKGIATRLSGGALDEDVNSYKKGAGADSRIGAALAQLRKSQAGAKAMEPERHFDPKDAALMESMQVGDAAAQHSASQQAGSWSEELNHLRTFQSTGGVTVTARTELNPHPQDADLINEYKKETATSTGRMYATALRSFGHYLRENNKKGIATRLSGGALDEDINSYKKGAGADSRIGAALAQLRKSQAGANAMELERHIDPKDAALMESMQVGDAAAQHSASQQAGSWPEEFLPAEGHDQDLGRMDEPGPSSSAPQPAQSTGILRGRRKPLYSEDAPLISGLEEALRSGNAAERTAKDLVGPLRAFGRWLLANNKTSIVDRLEKESLTDDAREFIEKGKGSRLLIRSIGLLRTFQSTGGVTVTATTELNPYLQDAALIIEYQNEAATSTGGVYATALRSFGHHLRENNKKGIATRLSGGALDEDVEAYKKDFGGIRTIDAALGQLRKSQAGAKAMERERYISPGPDPEGAALMEPRRAGDAAAQHSSQEVGSWPEKLLPAERHEQD**LVLGL**MDEPGPSSSLEPVARHDQASDPGDSIRPLNWRRDGQQFSEEPMAALARSNLPPSEEILINDEQDAAEL**RPAKRPRTL**DNPQGLAIERLLSEIAATPAPTHQQGASPWHAQPMMQASGHEDATAPHAAATYVAGAAAQHSAPQGAVSRPLVLPEGYDRDLRLMGKDGPSWPEVPPGQAQDIVQAGRQQPAWSASTWSPQMPLDFDWSMWPTLEAAPAPAARARSGTYGGLESLVHLDAPTPSELRDDAHFAPAPAARARSGTYGGLESLVHLDAPTPSELRDDAHFAPAPAARARSDTYGGLESLVHLDAPTPSELRDDAHFAPAPAARARSDTYGGLESLVHLDAPTPSELRDDAHFAAAPFARARSDAYRGFPLVDLTAPTPSESRDDANSVRPFPSTSANAQIGALDPTVSSHGHGLVLDDTEWLGDQHIDRDYGLQEQDLQRNDPDLAARTRFVNPLIALNYLRSNDDGVVLTEFQRIVYDDNGNDTADFLFLPVINGNPEDPNSRGN**H**WSLLFVDRSDRWRPVAYHY**D**SYGGLNNRDAAHLARR**LNLPLEL**ADMAQQQNTYD**C**GVFVVDGTRELVRQLAQGWEPDQLNLSNVVANRQALQNRLRG

Ç√

Ç√

Ç√

Ç√

Ç√

Ç√

Ç√

Ç√

Ç√

**Is-34**

MDFPSTKSVHEQSDSTGPQESSPAAPSAAAATFERQLSEIANSDGGGGAMPAASALQSAQSTWVLIGRQGKHLLYSEDARLISGLEKALIGNPTHRNAGDYLTSLRSFGGWLFANNKTSIVARLNDPSLTHDAREFEKRRPSNILAALNHLRTFQSTGGVTVTARTELNPHPQDADLINEYKKETATSTGRMYATALRSFGHYLRENNKKGIATRLSGGALDEDVKSYKKGAGADSRIGAALAQLRKSQAGAKAMEPERHIDPKDAALMESMQVGDAAAQHSASQQAGSWSEELNHLRTFQSTGGVTVTARTELNPHPQDADLINEYKKETATSTGRMYATALRSFGHYLRENNKKGIATRLSGGALDEDINSYKKGAGADSRIGAALAQLRKSQAGANAMELERHIDPKDAALMESMQVGDAAAQHSASQQAGSWPEEFLPAEGHDQDLGRMDEPGPSSSAPQPAQSTGILRGRRKPLYSEDAPLISGLEEALRSGNAAERTAKDLVGPLRAFGRWLLANNKTSIVDRLEKESLTDDAREFIEKGKGSRLLIRSIGLLRTFQSTGGVTVTATTELNPYLQDAALIIEYQNEAATSTGGVYATALRSFGHHLRENNKKGIATRLSGGALDEDVEAYKKDFGGIRTIDAALGQLRKSQAGAKAMERERYISPGPDPEGAALMEPRRAGDAAAQHSSQEVGSWPEKLLPAERHEQD**LVLGL**MDEPGPSSSLEPVARHDQASDPGDSIRPLNWRRDGQQFSEEPMAALARSNLPPSEEILINDEQDAAEL**RPAKRPRTL**DNPQGLAIERLLSEIAATPAPTHQQGASPWHAQPMMQASGHEDATAPHAAATYVAGAAAQHSAPQVAVSRPLVLPEGHDRDLRLMGKDGPSWPEVPPGQAQDIVQAGRQQPAWSASTWSPQMPLDFDWSMWPTLEAAPAPAARARSGTYGGLESLVHLDAPTPSELRDDAHFAPAPAARARSDTYGGLESLVHLDAPTPSELRDDAHFAPAPAVRARSGTYGGLESLVHLDAPTPSELRDDAHFAPAPAARARSDTYGGLESLVHLDAPTPSELRDDAHFAPAPAARARSDTYGGLESLVHLDAPTPSELRDDAHFAAAPFARARSDAYRGFPLVDLTAPTPSESRDDANSVRPFPSTSANAQIGALDPTVSSHGHGLVLDDTEWLGDQHIDRDYGLQEQDLQRNDPDLAARTRFVNPLIALNYLRSNDDGVVLTEFQRIVYDDNGNDTADFLFLPVINGNPEDPNSRGN**H**WSLLFVDRSDRWRPVAYHY**D**SYGGLNNRDAAHLARR**LNLPLEL**ADMAQQQNTYD**C**GVFVVDGTRELVRQLAQGWEPDQLNLSNVVANRQALQNRLRG

Ç√

Ç√

Ç√

Ç√

Ç√

Ç√

Ç√

Ç√

Ç√

Ç√

Ç√

**USDA110**

MDFPLTKSVHEQSGSTGPQESSPAAPSAAAATFERQLREISSSGVGGGAMPAAAALQPAQSTEVLIGRQCNQILYSEDARLISGLEKALTKGGAAKGTTNNYLRVLRGFGHWLSANNKKGISARLDDKKSLSQDAREFSGEGEPWKLLTAIGHLRNSQSTGEVVPITRHVEVNPYQQDAELIKEYQNEVASEMGKRDATALRNFSDYLRDNNKKGIVGRLRGELLNEDVKSYKKVSAFRSKIGVALDRLRKSSAGAKAMDLERNIDPEDAALKESRQVGDAAAQHSSSQKAGSWPEELLPAEGHDQNLLLGPMDEPGASSSAPQPTQSTGILRGRRRRPLYPEDAALISGLEKALIKGGAAEGTAKDHVRTLLSFGQRLYANNKDPIAARLDDEKSLTADARELFEKRPATLHRAIDHLRTSRSTGGIVPIAGRTELHPYPQDAALIKEYKNEVATDAGKRDATALRNFSDYLRDNNRPGIAAGLGTSFDGDVESYRKVAGADSRIGAALARLRKSQAGAEAMEHERHIDPEEAALRESRRVGDAAAQHIASQKGGSWPEELLPAEGHDQDLLLGLMDEPGPSSSAPQSAQSTWIVIGKRKQPLYSVDAPLISGLERALIKGGFSKSAAEQHGGSLRSFSRWLFAKEKPSIRDRLDNKSLTDGGEVLEFTGEGNPKRLVQAIDYLRTLRSTGEVPISRRAKLNPHPQNVAFINPEDTVLMEPRRVDAAAAQHSASQETGRRPEELPAEGRDQDLLLGLMDEPRSSSSLEPAARHDQAPDPGEPDRQQSPDEPMAALARSNRLPSEEVLINDEHDTAEL**RPAKRQRTL**NNPQGVAGERQLSEIANSGGQPTPAPTHQQGTSSWETQPMLLRSGYEDVTAPHAVAMYVGGAAAQHSARQRAVSRPLVLPEGYDQDLRLMVEDGPSWPEVPPEQAQDIVQAGQEPARPTVEAAPTHSARARSNTYGGIEVSFNPNSPASFELRDNAWSPAPGFPPPFAGPVPGHHQGAQQLGSPQGLSPVSAHSDDDALAWLSEELARQMQEPASPSTARAQDLYRGFEALLDPDVAELDDSAHFAPAPSARACSDTYGGLEVSFNPNSPTSFELRDNAWSRAPGFPPPFAGPVPGHHQGARQLGSPQGLSPVSAHSDDDALAWLSEELARQMQEPASPSTARAQDLYRGFGALLDPDAAELDDSAHFAPAPSARARSDTYDGLPLVDLTAPAPSPLRDDIVRRFPITSSDAQIGALNPIALSHNRGLVLEDTEWLGDEHILRDYQLQELDLQRSDSDLAARTRFVDPLEA**LRLRL**GAESDVLRVFHRIVHDRRDNDTADFLLLPVNDASATDRGR**H**WSLLFVDRSNRQRPVAYHY**D**SYGRYNETHARQLAER**LNLAL**QPAGMAQQQNTCD**C**GVFVVDGTRELVRQLAQGREPDQLNLSNVVANRQALQARLRG

Ç√

Ç√

Ç√

Ç√

Ç√

Ç√

**HH103**

MPAAAAPQPTQSKGILRRLSKSPLYSQDAPLILGLEKALIKGGAAERTARGYVRTLRSFGQWLFANNKDPIAARLEDEDPLTADAREFIGKDNPLRLLAAIVHLRTSQSTGGVVRIAGRTEYHPYPQDAALIEEYKNEAATDPGKKDASRKDATALRSFSDYLRDNNRPGIAAGLGTSFDGDVESYRKVAGADSRIGAALARLRKSQAGAEAMESSAPQPAQSPGIAIGRDNRPLYSEDAPLISGLEGALIKGGFSKSAAEQHGGSLRSFSRWLFAKEKPSIRARLDNQSLTDGGEVLEFTGQGNPKRLVQAIDYLRTLRSTGEVPISRRAKLNPHPQNVALINPEDTVLMEPRRVDAAAAQHSASQKTGSRPEELPAEGRDQDLLLGLMDEPGPSSSLEPAARHDQAPDPGEPDRQQSPDEPMAALARSNRLPSKEVLINDEHDTAEL**RPAKRQRTL**NNPQGVAGERQLGEIANSGGQPTPAPTHQQGTSSWETQPMLLRSGYEDVTAPHAVATYVGDAAAQHSARQRAVSRPLVLLEGYDQDLRLMVEDGPSWPDVPPEQAQDIVQAGQEPAWPTVEAAPTHSARARSNTYGGLEVSFNPNSPASFELRDNAWSPAPGFPLPFAGPVPGHHQGARQLGSPQGLSPVSAHSDDDALAWLSEELARQMQEPASPSTARAQDLYRGFEALLDPDAAELDDSAHFAPAPSARARSDTYGGLAVSFNPNSPASFELRDNAWSPAPGFPLPFAGPVPGHHQGARQLGSPQGLSPVSAHSDDDALAWLSEELARQMQEPASPSTARAQDLYRGFEALLDPDAAELDDSAHFAPAPSARARSDTYGGLAVSFNPNSPASFELRDNAWSPAPGFPPPFAGPVPGHHQGARQLGSPQGLSPVSAHSDDDALAWLSEELARHMQEPASPSTARAQDLYRGFEALLDPDAAELDDSAHFAPAPSARARSDTYGGLAVSFNPNSPASFELRDNAWSPAPGFPPPFAGPVPGHHQGARQLGSPQGLSPVSAHSDDDALAWLSEELARQMQEPASPSTARAQDLYRGFGALLDPDAAELDDSAHFAPAPSARARSDTYGGLPLVDLTAPAPSPLRDDIVRRFPITSSDAQIGALNPIALSHNRGLVLEDTEWLGDEHILRDYQLQELDLQRSDSDLAARTRFVDPLEA**LRLRL**GAESDVLRVFHRIVHDRRDNDTADFLFLPVNDASATDRGR**H**WSLLFVDRSNRQRPVAHHY**D**SYGRYNETHARQLAER**LNLAL**EPAGMAQQQNTYD**C**GVFVVDGTRELVRQLAQGREPDLLNLSNVVANRQALQARLRG

Ç√

Ç√

Ç√

Ç√

Ç√

Ç√

Ç√

Ç√

Ç√

Ç√

**XS1150 NopD**^1^

MDPYNFDPPNPTAWSPVQHAVLEEDQGGHAGQEGFEQHLAEARSPDPGPVSRGGRNYHPHLSAEHRDTIDKAIAEYAAQKNPQRNTVKRYTQALRRLGNDLGAHRITIDLRDHQSLVRHVKTYFPNDEDMKKGLGVLRAYHDRSYVASGGRPRTIPSAEDAPLSERLNASGMTSGSAARHDRSLRRFSNALNLAGYSISGLDHAARIEFAQKLFPNDELLLFALGKVRDAENVPGARASRKPSGRAVPSPALHLYPDDARIIDGLEKAELSMLKPEEKSRKKVVQNLARNQRRLGAWLQREGRGSIVSRLTGTSEQQKSLNDDHTDFKKSNRNADMGFDRLRSYLLLVEANAALGVCPEQAGGEPRRGESNSTWSPHLPYDFEWPTPEAVPDGSSAIYRGLDSFVDLPYTSQEVRDDAQSTPVGRAAARPPLFNGPSDAPAQSPDIFRGLQSFVDLPYTPQQMRDDAQSAPVSGAAAKPPLFTGPPDAPAQSSDIYRGLNSFVDLPYTPQQMRDDAQPAPVGGAAARPPLFTGPSDAPAQSSDIYRGLNSFVDLPYTPQQMRDDAQSAPVGWAAAKPPVFTEPSDVATQSSQIYHGLNSFVDLPYTPQQMRDDAQSAPVGGAAARLVLFTEPSDAPTQSSHIYRDLNPFVDLPYTPQQMRDDAQSAPVGGAAARPPLFTEPSDTPAQSSDIYRGLNSFVDLPYTPQQMRDDAQSAPVLSPAGKPAFFVGRSGVLQELEDIGYRVDEDWQDGSQPVPDFLIDVLDNIRVLPTQFSGPTQLSMNGETYSITLGPRGRRVAQFIHHPRPSPVPGAQIGPSATVASSGHRSGPVLGPTQWLGDEHIQRDYELLAQELQQNNPDLAARTRFVDPLIAQMLRSPSKEVAERALGWVRPGTADFLFLPVSDASDTDRHQRGS**H**WSLLLVDRRDRGRRVAYHY**D**STQGYNDGLAAELAGRLDANLQQAPIRQQQNSYD**C**GVFVLDGTRELVRRLAARRPDLNLNNLVISRQELRDRLGAGVGFN

﻿PAQSPDIFRGLQSFVDLPYTPQQMRDDAQSAPVSGAAAKPPLFTGPPDA

﻿PAQSPDIFRGLQSFVDLPYTPQQMRDDAQSAPVSGAAAKPPLFTGPPDA

﻿PAQSPDIFRGLQSFVDLPYTPQQMRDDAQSAPVSGAAAKPPLFTGPPDA

﻿PAQSPDIFRGLQSFVDLPYTPQQMRDDAQSAPVSGAAAKPPLFTGPPDA

﻿PAQSPDIFRGLQSFVDLPYTPQQMRDDAQSAPVSGAAAKPPLFTGPPDA

﻿PAQSPDIFRGLQSFVDLPYTPQQMRDDAQSAPVSGAAAKPPLFTGPPDA

﻿PAQSPDIFRGLQSFVDLPYTPQQMRDDAQSAPVSGAAAKPPLFTGPPDA

﻿PAQSPDIFRGLQSFVDLPYTPQQMRDDAQSAPVSGAAAKPPLFTGPPDA

﻿PAQSPDIFRGLQSFVDLPYTPQQMRDDAQSAPVSGAAAKPPLFTGPPDA

Ç√

﻿PAQSPDIFRGLQSFVDLPYTPQQMRDDAQSAPVSGAAAKPPLFTGPPDA

**MAFF303099**

MDQRKITSRVTALPQVQDAGLEEGQAVQARQVGFEQHLAEARRLFDQADESPTNPEELLRLEQGFREVLQRRQDDQVAEALRPLFDDRADEPPANPEELLRLEQGFREVLQRRQDDQAVSSFFSDPGMPAGPGDHNSIVTDAFAAAGSGHAGVEAAAPPVLAASQQQIRPSPDALDQGNHLPPQGGIINNEHSTAPL**RPAKRQRAV**DRPQAVAIQQQLSEIGNSGGRVPIQPPTQQLGELPLQGVPVQGTGSEHIGRLHAGAAPSARSEAPPAAIEDSINVSFAVPKDFSHGTQRVPDAMLPFLDRPGPLPDAGQARQAGFEQHVAEPRRADPVASGARASRYHHLSDEHRDLIDRAIAHSQEKYSETTARKYTFALSRLANDLSARGQAIDLRNHKSLVDHVGAFFPKDVDMKSALKALRAYHEPGYSATAGGPAASYPHLSAEHRDVIDKAIDRAAAQQNQSADTLRIYSNALRRLANDLGARGQATDLKNHQSLVDHLDTFFPNDQNIKTALNVLRAYHDPGNAATGWWPAAVPSKADARILEKLSSDSGLALSTRVVYGRLLRRFSEELESRGQTISGLDHNSRTELAEA**LFPGNKKLRFA**LQRVHNAEVPEALRPLFDNRADKPPTNPEELLRLEQGFREVLQQRQGDQAASSLFGNPGMPAGPEDPNRSVSDAFASSGHAGVEAAAPPVLAASQQQIRPWPDAFDQGNHLPPERVIINNEHDTALLRPAERQRALNTPQAAAIQQPLSEIGNSGGRVPMQPPTQQLGELPLEGVPVQRTGSEHIGRLHAEAAPSARAEAPPAAIENSINVSFAVPKGFSHGTQRVPDAMLSFLDRPGPLPDAGQARQAGFEQHVAEPRRAEPVASGARATGYRHLSDEHRDLIDKAIAHAAAQQKYSESTVLKYRYALRRLANDLGARGQATDLKNHQSLVDHLDAFFPKNDDMKRALNVLRAYHEPGYSATVGAPANRYPHLSDEHRDVIDKAIAHAEAQQHHSAPTLRIYSNALRRLANDLGARGQATDLKNHQSLVDHLNTFFPKDTDIRDIRPALNVLRAYHEPGYSATGRWPVTVPSKADAHVLEQVTSDSSLAPSTRVVYGHSLRRFSEALDRRGRTISGLDHDSRIEFAEVLFPGNDYLRWALERVRDAKPASDRIVADALAAAGSGHAGVEAAAPPVLAASQQQIRPWPDALDQGNLLPPERFIINNEHSTAPLRPAERQRALNTPQAAAIQQQPSEIGNSGGRMPMQPPMWQLGELPLQGVPVQGTGSEHIGRLHAGAAPSARSEAPPAAIEDSINVSFAVPKGFSHVTQRVPEAMLSSLYHYGLLPDADKPEWNYEIKGHGYTARRPEEGNDVWLLHRGAIREAGAAAVPARAPGPALPATARLSDTHLGVPLVDLTTSSDAHIEALPSGSSNLPRGAVLGATQLLGDEHIQRDYEFLEQQLQQADPALAARTRLVDPSVSHLLRHMEQQDARGTLQSIYNRNAGPSDFLFVPVNDGVGIDRGT**H**WSLLLVDRRDPERAVAYHY**D**SIQQNEQRYNDAPARKLATRLDATLVTPDMAQQKNAVDCGVFVVDGTRELVRRLANEERPDQQ**LPLHL**NYLVADRQALQNRLREGRLPHELAASPAEALAAPGSQVQHAALQEQQARQVAPAPLERHLGKTREAEDKLTSTLDRSNRVNSGGVVINTERYTAPL**RPAKRQRTD**NSQSLAIGRQPSEANTTSIGQASDQARADLMASSRSRERSDAGR

***Xcv.* XopD**^2,3^

MDRIFNFDYKKYREMTEAADDYRNSPPHEEQRENHGAGYNMHPLLESLPRRNPTQVHADGSVHQMRAAAPTSRTHRDYLKILELISAYGDGKGIPELQRSFPSFAAFLMDSGLSHVNGRQMLQELNEDQRDQVIHQIIRRIEYCADPEYREVALSRLESDCSGKITLSQRTLDRIDKAKAKAEAEAEAKAKAKAEAKAEAKARVEAGAQCKINEIMEYIPRYEALEKVPVRVRFHAYLRGDGSFGPGLPGILRYMTPDQKKRLYLASERRKLALAAPKSKPTPKSKPLKGVFRTLHQKPNLLLEISSKFSNRAYSINDSSSGYLSQADLEEMVDEETGELTRLGEAVISGASQGIQTAIRANFRMRYQQPDLPPYSPPQAFHRPEETWNPHTPAGSSYSSLFPPTPSGGWPQNASGEWHPDTPAGYSHRAWPAQPEASSSTFDDLESLDYRQNYGYRE**FDLNTP**QEIEQPGWWQQATPAQSTDSTFDGLSSMSHYGSE**FDLNIP**QQEEYPNNHGTQTPMGYSAMTPERIDVDNLPSPQDVADPELPPVRATSWLLDGHLRAYTDDLARRLRGEPNAHLLHFADSQVVTMLSSADPDQQARAQRLLAGDDIPPIVFLPINQPNA**H**WSLLVVDRRNKDAVAAYHY**D**SMAQKDPQQRYLADMAAYHLGLDYQQTHEMPIAIQSDGYS**C**GDHVLTGIEVLAHRVLDGTFDYAGGRDLTDIEPDRGLIRDRLAQAEQAPAESSIRQVPARSNEQ**KKKKSK**WWKKF

**Supplementary Data 2**

1. Alignment analysis of the C-terminus of Bel2-5 (from 1,144-1,328aa) and its homologs in several selected rhizobial and pathogenic bacteria, including *Bradyrhizobium japonicum* Is-34 (KGT79298.1, from 1,180-1,364aa), *Bradyrhizobium diazoefficiens* USDA 110 (BAC53509.1, from 1,261-1,441aa), *Sinorhizobium fredii* HH103 (CCE98838.1, from 1,138-1318), *Bradyrhizobium* sp. XS1150 (AWS20424.1, from 835-1,011aa), *Mesorhizobium loti* MAFF303099 (BAB52630.1, from 1,430-1,614aa), dan *Xanthomonas campestris* pv. *vesicatoria* (DAA34040.1, from 552-729aa). The yeast ULP (DAA11408.1, 447-621aa) is included into the alignment. The predicted triad catalytic residues (H, D, and C) are indicated by red.

Bel2-5 EWLGDQHIDRDYGLQEQDLQRNDPDLAARTRFVNPLIALNYLRSNDDGVVLTEFQRIVYD
Is-34 EWLGDQHIDRDYGLQEQDLQRNDPDLAARTRFVNPLIALNYLRSNDDGVVLTEFQRIVYD
USDA110 EWLGDEHILRDYQLQELDLQRSDSDLAARTRFVDPLEALR-LRLGAESDVLRVFHRIVHD
HH103 EWLGDEHILRDYQLQELDLQRSDSDLAARTRFVDPLEALR-LRLGAESDVLRVFHRIVHD
XS1150 NopD QWLGDEHIQRDYELLAQELQQNNPDLAARTRFVDPLIAQM-LRSPSKEVAERALGWVRPG
MAFF303099 QLLGDEHIQRDYEFLEQQLQQADPALAARTRLVDPSVSHL-LRHMEQQDARGTLQSIYNR
*Xcv*. XopD SWLLDGHLRAYTDDLARRLRGEPN--AHLLHFADSQVVTM--LSSADPDQQARAQRLLAG
Yeast Ulp RWLNDTIIE----FFMKYIEKSTPNTVAFNSFFYTNLSER--------GYQGVRRWMKRK
consensus ..*.* ...... .................... ..... ... .. ... ..... .


Bel2-5 DNGNDTADFLFLPVINGNPEDPNSRGN**H**WSLLFVDRSDRWRPVAYHY**D**S----YGGLNNR
Is-34 DNGNDTADFLFLPVINGNPEDPNSRGN**H**WSLLFVDRSDRWRPVAYHY**D**S----YGGLNNR
USDA110 RRDNDTADFLLLPVNDASATD---RGR**H**WSLLFVDRSNRQRPVAYHY**D**S----YGRYNET
HH103 RRDNDTADFLFLPVNDASATD---RGR**H**WSLLFVDRSNRQRPVAHHY**D**S----YGRYNET
XS1150 NopD -----TADFLFLPVSDASDTDRHQRGS**H**WSLLLVDRRDRGRRVAYHY**D**S----TQGYNDG
MAFF303099 NAG--PSDFLFVPVNDGVGID---RGT**H**WSLLLVDRRDPERAVAYHY**D**SIQQNEQRYNDA
*Xcv*. XopD ---DDIPPIVFLPINQP--------NA**H**WSLLVVDRRNKDAVAAYHY**D**SMAQ-----KDP
Yeast Ulp KTQIDKLDKIFTPINLN--------QS**H**WALGIIDLKKK---TIGYV**D**SLSN----GPNA
consensus ..........*. .. . .. **.*...*.... .......**....... ..


Bel2-5 DAAHLARRL--------------NLPLEL-------ADMAQQQNTYD**C**GVFVVDGTRELV
Is-34 DAAHLARRL--------------NLPLEL-------ADMAQQQNTYD**C**GVFVVDGTRELV
USDA110 HARQLAERL--------------NLALQP-------AGMAQQQNTCD**C**GVFVVDGTRELV
HH103 HARQLAERL--------------NLALEP-------AGMAQQQNTYD**C**GVFVVDGTRELV
XS1150 NopD LAAELAGRL--------------DANLQQ-------APIRQQQNSYD**C**GVFVLDGTRELV
MAFF303099 PARKLATRL--------------DATLVT-------PDMAQQKNAVD**C**GVFVVDGTRELV
*Xcv*. XopD QQRYLADMA--------------AYHLGLDYQQTHEMPIAIQSDGYS**C**GDHVLTGIEVLA
Yeast Ulp MSFAILTDLQKYVMEESKHTIGEDFDLIH-------LDCPQQPNGYD**C**GIYVCMNT--LY
consensus .. .. .................. *..............*.....**..*......*.


Bel2-5 RQLAQGWEPDQ---LNLSNVVANRQALQNRLRG
Is-34 RQLAQGWEPDQ---LNLSNVVANRQALQNRLRG
USDA110 RQLAQGREPDQ---LNLSNVVANRQALQARLRG
HH103 RQLAQGREPDL---LNLSNVVANRQALQARLRG
XS1150 NopD RRLAARR-PD----LNLNNLVISRQELRDRLGA
MAFF303099 RRLANEERPDQQLPLHLNYLVADRQALQNRLR-
*Xcv*. XopD HRVLDG-TFDYAGGRDLTDIEPDRGLIRDRLAQ
Yeast Ulp GSADAPLDFDYKDAIRMRRFIAH-LILTDALK-
consensus ...... ..*....................*..

1. Alignment analysis of BEL2-5 and ErnA of *Bradyrhizobium* ORS3257 (SPP98361.1, from 153-238). BEL2-5 and ErnA share similarity at 85 amino acids from 706 to 790 aa of Bel2-5 sequences. The alignment was done by MEGA 7 using CLUSTALW algorithm^4^. Results highlighted by black box indicate identical- and by grey box indicate similar- amino acids among the aligned homologs.

Bel2-5 LPAERHEQDLVLGLMDEPGPSSSLEPVARHDQASDPGDSIRPLNWRRDGQQFS-EEPMAA
ORS3257 ErnA LPAEDYAQELLWAMLENAGSSSSLEPTERHDHAVASGAAVRSFNSRQDDQRTSGAHEGSS
consensus **** *.*. .... * ****** *** * * .* * * * * *.


Bel2-5 LARSNLPPSEEILINDEQDAAELRPA
ORS3257 ErnA VPPSQDSPPAPFIVHNDRFTALFVPA
consensus . *. * .. . * **

**Supplementary Figure 1**

1. Original whole western blot gel of Bel2-5 detection using anti-Bel2-5 antibody. For the final figure shown in Fig. 3b, the gel picture was straightened 7^o^ and cropped on the horizontal line of the predicted Bel2-5 bands. M represent protein marker in kDa. The -/+ represents absence or presence of genistein, respectively.


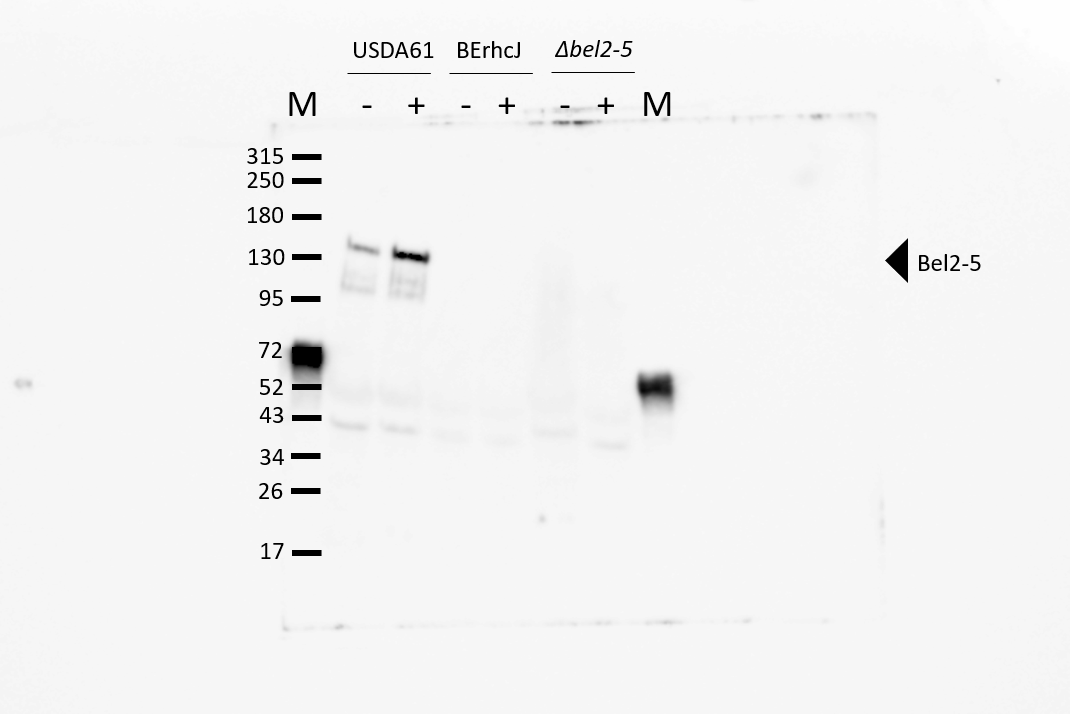


**(b)** Original whole western blot gel of NopA detection using anti-NopA antibody. For the final figure shown in Fig. 3b, the gel picture was flipped horizontally and cropped on the horizontal line of the predicted NopA bands. M represent protein marker in kDa. The -/+ represents absence or presence of genistein, respectively.


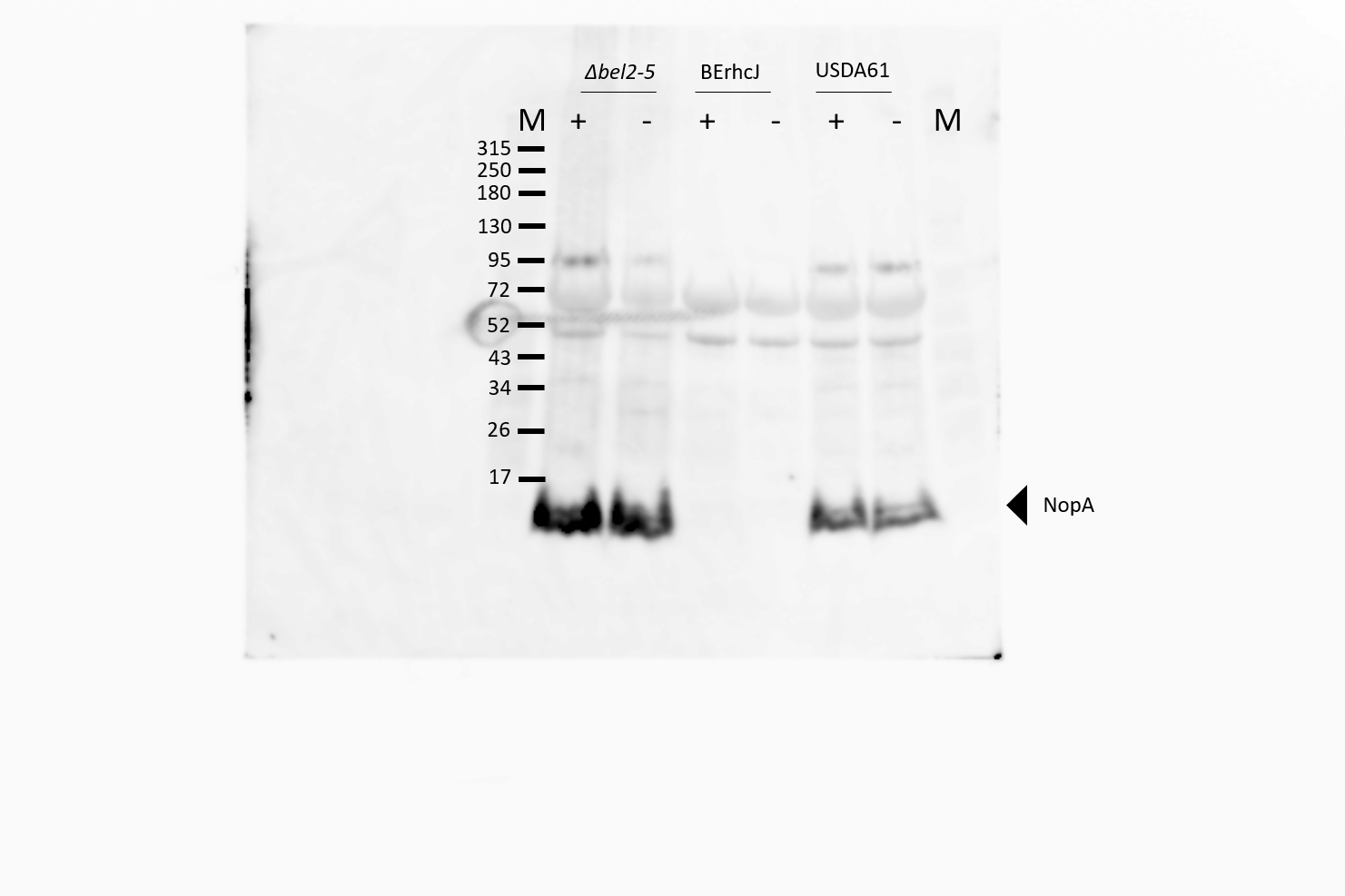


**Supplementary Figure 2**

qRT-PCR of selected soybean DEGs (defense- and symbiosis-related genes) inoculated with mock (water), *B. elkanii* USDA61 wild-type, and *bel2-5* deletion mutant (*∆bel2-5*). Values represent mean ± SD (*n*=3 independent soybean roots measured two times). Statistical analyses (Student’s *t-test*) were performed for comparison of each inoculation with the mock treatment or wild-type of USDA61. **P* < 0.05; ***P* < 0.01. FC means fold change in the roots of En1282 inoculated with USDA61 versus the roots of En1282 inoculated with *bel2-5* deletion mutant.

**Defense related-genes**

FC= 0.3053

*

**Symbiosis related-genes**

**

**

*

FC= 4.378

*

FC= 2.4416

**

**

Relative expression

Relative expression

Relative expression

**

FC= 0.2283

**

FC= 0.2221

Relative expression

*

Relative expression

Relative expression

**

FC= 0.3831

**Supplementary Table 1.** List of (putative) *nop* genes of *Bradyrhizobium elkanii* USDA61

| No | Gene ID^a^ | Annotation | Nop homology^b^ | Upstream tts-box^c^ | Characteristics of encoded proteins | Mutation |
| --- | --- | --- | --- | --- | --- | --- |
| 1 | BBB94673.1 | BE61_00830 | NopAZ | ni. | put. nucleoside phosphorylase | Not mutated |
| 2 | BBB96811.1 | BE61_22420 | NopAF | TB1. **GTCAG**T**T**TGTC**G**TG**AG**TTGCCCCGCATG | put. transpeptidase | Not mutated |
| 3 | BBB97652.1 | BE61_30860 | NopBI | ni. | put. kinase Y4dM - HipA-like protein | Not mutated |
| 4 | BBB98366.1 | BE61_38060 | NopAQ | ni. | put. ABC transporter periplasmic binding protein | Not mutated |
| 5 | BBB98368.1 |  | NopAE | ni. | put. qedA | Not mutated |
| 6 | BBB99017.1 | BE61_44580 | put. new Nop | TB2. T**TCAG**C**T**TGTC**G**AA**AG**TGTCGTCGTCA**A** | hypothetical protein | Not mutated |
| 7 | BBB99736.1 | BE61_51850 | NopAJ | ni. | hypothetical protein | Not mutated |
| 8 | BBB99748.1 | BE61_51970 | Bel2-5 | TB3.  A**TCAG**G**T**TCTC**G**AC**AG**CTTCCCCATTT**A** | put. peptidase C48, SUMO/Sentrin/Ubl1 | BE2-5/ Δ*bel2-5* |
| 9 | BBC00345.1 |  | NopAP | ni. | put Kup transporteur : potassium transporter | Not mutated |
| 10 | BBC00974.1 | BE61_64350 | put. new Nop | TB4. **GTCAG**C**T**TCAT**G**CT**AG**CTCGGCTGGAT**A** | put. transporter | Not mutated |
| 11 | BBC00975.1 |  | put. new Nop | TB4. | put. transporter | Not mutated |
| 12 | BBC02156.1 |  | NopM | TB5. **GTCAG**C**T**GATC**G**AC**AG**GTAGGCCTGCA**A** | E3-ubiquitin ligase - secreted effector | BEnopM1 |
| 13 | BBC02158.1 | BE61_76200 | ErnA | TB6. **GTCAG**C**T**TGTC**G**AC**AG**CTGATCCCAAT**A** | secreted effector | BEernA |
| 14 | BBC02248.1 |  | NopP | TB7.  **GTCAG**C**T**TACG**G**TC**AG**TTTCGCCTGGCT | secreted effector | BEnopP1 |
| 15 | BBC02288.1 | BE61_77510 | NopT | TB8. **GTCAG**C**T**TGCG**G**CAATATCCTTCAGAGG | cysteine protease - secreted effector | Not mutated |
| 16 | BBC02292.1 | BE61_77550 | NopAO | TB9. **GTCAG**C**T**AGCG**G**TC**AG**GGAAGGAGGCT**A** | hypothetical protein | Not mutated |
| 17 | BBC02318.1 | BE61_77810 | NopM | TB10. **GTCAG**C**T**TATC**G**AC**AG**CTGAGCTTCCT**A** | E3-ubiquitin ligase - secreted effector | Not mutated |
| 18 | BBC02321.1 | BE61_77840 | put. new Nop | TB11. **GTCAG**C**T**TCTC**G**AA**AG**CTTGCTCTGGT**A** | hypothetical protein | Not mutated |
| 19 | BBC02355.1 |  | InnB | TB12. **GTCAG**C**T**TTTC**G**AA**AG**CTAGGTCCCAT**A** | hypothetical protein | BEinnB |
| 20 | BBC02356.1 |  | NopAD | ni. | put. pectinesterase | Not mutated |
| 21 | BBC02357.1 | BE61_78200 | NopAC | ni. | put. endo-polygalacturonase | Not mutated |
| 22 | BBC02364.1 | BE61_78270 | NopAJ | TB13. **GTCAG**C**T**TCTC**G**AC**AG**CTAAATCGGCT**A** | hypothetical protein | Not mutated |
| 23 | BBC02365.1 | BE61_78280 | put. new Nop | TB14. **GTCAG**G**T**TTTC**G**TC**AG**ATCCGATGTGT**A** | hemolysin-type calcium-binding protein | Not mutated |
| 24 | BBC02368.1 | BE61_78310 | NopAA | TB15. **GTCAG**C**T**TCTC**G**AA**AG**CCAACCCTCCT**A** | put. glycoside hydrolase | Not mutated |
| 25 | BBC02379.1 | BE61_78420 | put. new Nop | TB16. | hypothetical protein | Not mutated |
| 26 | BBC02380.1 | BE61_78430 | put. new Nop | TB16. | hypothetical protein | Not mutated |
| 27 | BBC02381.1 | BE61_78440 | NopM | TB16. **GTCAG**C**T**TCTC**G**AA**AG**CCAAGCCTTGT**A** | E3-ubiquitin ligase - secreted effector | BEnopM2 |
| 29 | BBC02441.1 |  | put. new Nop | TB17.  **GTCAG**C**T**TCTG**G**AA**AG**CTGAGCGCCAC**A** | put. tyrosine aminotransferase | Not mutated |
| 30 | BBC02443.1 | BE61_79060 | put. new Nop | TB18. **GTCAG**G**T**TCTC**G**AC**AG**ATATCGCACAT**A** | put. peptidase C48, SUMO/Sentrin/Ubl1 | Not mutated |
| 31 | BBC02518.1 | BE61_79810 | NopBW | TB19. **GTCAG**C**T**GCCC**G**TC**AG**CCAAGCTGTCT**A** | hypothetical protein | Not mutated |
| 32 | BBC02524.1 | BE61_79870 | NopAR | TB20. **GTCAG**G**T**TTTG**G**AA**AG**TTAACATCAGT**A** | hypothetical protein | Not mutated |
| 33 | BBC02525.1 | BE61_79880 | NopAR | TB20. | hypothetical protein | Not mutated |
| 34 | BBC02528.1 | BE61_79910 | NopAM | TB21. **GTCAG**C**T**TCTC**G**AA**AG**CTGCACCTGCA**A** | hypothetical protein | Not mutated |
| 35 | BBC02537.1 | BE61_80000 | NopZ | TB22. | Y4yJ, T3SS stalk | Not mutated |
| 36 | BBC02539.1 | NolV | NopV | TB22. | T3SS stator | Not mutated |
| 37 | BBC02540.1 | NolU | NopU | TB22. | T3SS inner rod | Not mutated |
| 38 | BBC02542.1 |  | NopB | TB22. **GTCAG**G**T**TTTC**G**TC**AG**CTCACCGTACT**A** | T3SS pili associated Nop | Not mutated |
| 39 | BBC02544.1 | NopL | NopL | TB23. **GTCAG**C**T**TCTC**G**AA**AG**CTAACTCTCTT**A** | secreted effector | BEnopL |
| 40 | BBC02550.1 | BE61_80130 | put. new Nop | TB24. **GTCAG**C**T**TTCG**G**TC**AG**CTCGCTTCTCT**A** | hypothetical protein | BEorf48 |
| 41 | BBC02552.1 | NopX | NopX | TB25. **GTCAG**C**T**TCTC**G**AA**AG**GTTGTCCGTTTT | T3SS translocon | Not mutated |
| 42 | BBC02554.1 | NopC | NopC | TB26. **GTCAG**C**T**TGTC**G**AA**AG**CTAATGCTTCC**A** | secreted effector | Not mutated |
| 43 | BBC02555.1 | NopA | NopA | TB26. | T3SS pili component | Not mutated |
| 44 | BBC02556.1 | SctD | NopY | TB26. | Y4yQ, T3SS outer MS ring protein | Not mutated |
| 45 | BBC02557.1 | BE61_80200 | NopAL | TB26. | hypothetical protein | Not mutated |
| 46 | BBC02558.1 | BE61_80210 | put. new Nop | TB26. | hypothetical protein | Not mutated |
| 47 | BBC02569.1 | NopM | NopM | TB27. **GTCAG**CTTATC**G**AC**AG**CTACGTCTCGT**A** | E3-ubiquitin ligase - secreted effector | Not mutated |
| 48 | BBC02610.1 | NopP | NopP | TB28. **GTCAG**C**T**TGCG**G**TA**AG**CTAGGCTGTGT**A** | secreted effector | BEnopP2 |
| 49 | BBC02571.1 | BE_80340 | Mlr6331 | ni. | hypothetical protein | BEorf91 |
| 50 | BBC03520.1 |  | NopBH | ni. | put. aminopeptidase | Not mutated |
| 51 | BBC03688.1 | BE61_91540 | NopAG | TB29. **GTCAG**C**T**TTCT**G**TC**AG**CTCGTCCAAAT**A** | hypothetical protein | Not mutated |
| 52 | BBC03726.1 | BE61_91920 | put. new Nop | TB30. A**TCAG**GTTCTC**G**AC**AG**CTTCCCCATTT**A** | put. peptidase C48, SUMO/Sentrin/Ubl1 | Not mutated |
| 53 | BBC03733.1 | BE61_91990 | put. new Nop | TB31. A**TCAG**GTTCTC**G**AC**AG**CTTCCCCATTT**A** | put. peptidase C48, SUMO/Sentrin/Ubl1 | Not mutated |
| 54 | BBC03821.1 | BE61_92870 | put. new Nop | TB32. **GTCAG**CTTTTG**G**AA**AG**CTAGCCCGAAT**A** | hypothetical protein | Not mutated |

**^a^**Identity number used in National Center for Biotechnology Information (NCBI)

**^b^**Homologies determined from *nop* genes described in Kimbrel et al.^4^, Staehelin, Krishnan^5^, Okazaki et al.^6^, and Teulet et al.^7^

**^c^**ni. means *tts* box not identified in the upstream region of the gene.

**Supplementary Table 2.** The up-regulated DEGs in *Glycine max* roots during interaction with *B. elkanii* wild-type as compared with *bel2*-5 deletion mutant

| No. | GO annotation ID^a^ | Classification^b^ | ID | Similarity | Fold change^c^ | *P*-value |
| --- | --- | --- | --- | --- | --- | --- |
| 1 | N.A. | N.A. | NP_001238055.2 | Uncharacterized LOC100527571 | 5.727963 | 0.044304 |
| 2 | GO:0015297;  GO:0016020;  GO:0042910;  GO:0055085 | MF_Antiporter activity;  CC_Membrane;  MF_Xenobiotic transmembrane transporter activity;  BP_Transmembrane transport | NP_001342385.1 | MATE family protein | 5.652260 | 0.041811 |
| 3 | GO:0004222;  GO:0006508;  GO:0008270;  GO:0031012 | MF_Metalloendopeptidase activity;  BP_Proteolysis;  MF_Zinc ion binding;  CC_Extracellular matrix | XP_003519381.2 | Metalloendoproteinase 5-MMP | 5.305287 | 0.036922 |
| 4 | GO:0000287;  GO:0016831;  GO:0030976 | MF_Magnesium ion binding;  MF_Carboxy-lyase activity;  MF_Thiamine pyrophosphate binding | XP_003516954.1 | Pyruvate decarboxylase 2 | 4.185898 | 0.003793 |
| 5 | GO:0004650;  GO:0005975 | MF_Polygalacturonase activity;  BP_Carbohydrate metabolic process | XP_014634801.1 | Polygalacturonase At1g48100 | 4.010149 | 0.012689 |
| 6 | GO:0004553;  GO:0005975 | MF_Hydrolase activity, hydrolyzing O-glycosyl compounds;  BP_Carbohydrate metabolic process | NP_001238474.1 | Glucan endo-1,3-beta-glucosidase | 3.858778 | 0.004659 |
| 7 | GO:0005504;  GO:0009627 | MF_Fatty acid binding;  BP_Systemic acquired resistance | NP_001236851.1 | Uncharacterized LOC100526983 | 3.852123 | 0.029271 |
| 8 | GO:0016491;  GO:0055114 | MF_Oxidoreductase activity;  BP_Oxidation-reduction process | XP_003525310.1 | 1-aminocyclopropane-1-carboxylate oxidase 1 | 3.735428 | 0.022689 |
| 9 | N.A. | N.A. | XP_003543272.1 | Uncharacterized LOC100793429 | 3.500760 | 0.020648 |
| 10 | N.A. | N.A. | XP_003542565.1 | Putative mediator of RNA polymerase II transcription subunit 26 | 3.495379 | 0.003003 |
| 11 | N.A. | N.A. | XP_003543269.1 | Uncharacterized LOC100306608 | 3.483884 | 0.00677 |
| 12 | GO:0006631;  GO:0045300;  GO:0055114 | BP_Fatty acid metabolic process;  MF_Acyl-[acyl-carrier-protein] desaturase activity;  BP_Oxidation-reduction process | NP_001236391.1 | Stearoyl-ACP desaturase | 3.299072 | 0.016735 |
| 13 | GO:0005504;  GO:0009627 | MF_Fatty acid binding;  BP_Systemic acquired resistance | NP_001237117.1 | Uncharacterized LOC100527769 | 3.265919 | 0.020533 |
| 14 | GO:0016021 | CC_Integral component of membrane | NP_001238685.1 | Uncharacterized LOC100306683 | 3.152143 | 0.047566 |
| 15 | GO:0008271;  GO:0008272;  GO:0016021;  GO:0055085 | MF_Secondary active sulfate transmembrane transporter activity;  BP_Sulfate transport;  CC_Integral component of membrane;  BP_Transmembrane transport | XP_003532966.1 | High affinity sulfate transporter 2 | 3.079233 | 0.006652 |
| ***16 | GO:0008033  GO:0052381  GO:0009691  GO:0006400  GO:0005739 | BP_tRNA processing  MF_tRNA dimethylallytransferase activity  BP_cytokinin biosynthetic process  BP_tRNA modification  CC_mitochondrion | XP_003550509.1 | Adenylate isopentenyltransferase 5, chloroplastic | 3.057860 | 0.012072 |
| 17 | GO:0010181;  GO:0016491;  GO:0055114 | MF_FMN binding;  MF_Oxidoreductase activity;  BP_Oxidation-reduction process | XP_006596932.1 | Peroxisomal glycolate oxidase | 3.048586 | 0.004233 |
| 18 | GO:0000287;  GO:0016831;  GO:0030976 | MF_Magnesium ion binding;  MF_Carboxy-lyase activity;  MF_Thiamine pyrophosphate binding | XP_003520410.2 | Pyruvate decarboxylase 2 | 2.903622 | 0.003393 |
| 19 | GO:0055114 | BP_Oxidation-reduction process | XP_003526673.1 | Alcohol dehydrogenase 1 | 2.893131 | 0.032375 |
| 20 | GO:0004222;  GO:0006508;  GO:0008270;  GO:0031012 | MF_Metalloendopeptidase activity;  BP_Proteolysis;  MF_Zinc ion binding;  CC_Extracellular matrix | XP_003519371.1 | Metalloendoproteinase 5-MMP | 2.803750 | 0.019751 |
| 21 | N.A. | N.A. | XP_006582335.1 | Uncharacterized LOC100776758 | 2.736782 | 0.02478 |
| 22 | GO:0046856 | BP_Phosphatidylinositol dephosphorylation | XP_003549972.1 | Type I inositol polyphosphate 5-phosphatase 2 | 2.682344 | 0.033987 |
| 23 | N.A. | N.A. | NP_001235642.2 | Alpha-amylase inhibitor/lipid transfer/seed storage family protein | 2.678903 | 0.049001 |
| 24 | GO:0016491;  GO:0055114 | MF_Oxidoreductase activity;  BP_Oxidation-reduction process | NP_001241598.1 | Probable 2-oxoglutarate/Fe(II)-dependent dioxygenase-like | 2.643939 | 0.034455 |
| 25 | GO:0016021 | CC_Integral component of membrane | NP_001236984.2 | GASA domain-containing protein | 2.556790 | 0.04043 |
| 26 | N.A. | N.A. | XP_003531265.1 | Uncharacterized LOC100814945 | 2.523680 | 0.02483 |
| 27 | GO:0005506;  GO:0016705;  GO:0020037;  GO:0055114 | MF_Iron ion binding;  MF_Oxidoreductase activity, acting on paired donors, with incorporation or reduction of molecular oxygen;  MF_Heme binding;  BP_Oxidation-reduction process | XP_003548215.1 | Cytochrome P450 78A3-like | 2.490123 | 0.043449 |
| 28 | GO:0031305;  GO:0033617 | CC_Integral component of mitochondrial inner membrane;  BP_Mitochondrial respiratory chain complex IV assembly | NP_001236635.1 | Uncharacterized LOC100500345 | 2.476597 | 0.006811 |
| 29 | GO:0004672;  GO:0005524;  GO:0006468;  GO:0016021 | MF_Protein kinase activity;  MF_ATP binding;  BP_Protein phosphorylation;  CC_Integral component of membrane | XP_003539731.1 | Uncharacterized LOC100527596 | 2.467596 | 0.048422 |
| 30 | GO:0016021 | CC_ Integral component of membrane | NP_001237498.2 | Gibberellin-regulated protein-like | 2.467517 | 0.0014 |
| 31 | N.A. | N.A. | XP_003527455.1 | Uncharacterized LOC100818450 | 2.428902 | 0.027527 |
| 32 | N.A. | N.A. | NP_001336160.1 | SHOOT2 protein | 2.340643 | 0.018037 |
| 33 | N.A. | N.A. | XP_003540435.1 | Uncharacterized LOC100776723 | 2.337458 | 0.003467 |
| 34 | GO:0004857;  GO:0030599;  GO:0042545 | MF_Enzyme inhibitor activity;  MF_Pectinesterase activity;  BP_Cell wall modification | XP_006587077.1 | Probable pectinesterase/pectinesterase inhibitor 12 | 2.318200 | 0.047028 |
| 35 | GO:0005504;  GO:0009627 | MF_Fatty acid binding;  BP_Systemic acquired resistance | NP_001238322.1 | Alpha-amylase inhibitor/lipid transfer/seed storage family protein | 2.283184 | 0.044801 |
| 36 | GO:0004623;  GO:0005509;  GO:0006644;  GO:0016042;  GO:0050482 | MF_Phospholipase A2 activity;  MF_Calcium ion binding;  BP_Phospholipid metabolic process;  BP_Lipid catabolic process;  BP_Arachidonic acid secretion | NP_001235480.1 | Uncharacterized LOC100500260 | 2.281150 | 0.032754 |
| 37 | GO:0004601;  GO:0006979;  GO:0020037;  GO:0042744;  GO:0055114 | MF_Peroxidase activity;  BP_Response to oxidative stress;  MF_Heme binding;  BP_Hydrogen peroxide catabolic process;  BP_Oxidation-reduction process | XP_025981658.1 | Peroxidase 15-like | 2.218147 | 0.04975 |
| 38 | GO:0005576;  GO:0019953 | CC_Extracellular region;  BP_Sexual reproduction | NP_001242200.2 | Beta-expansin | 2.211779 | 0.009414 |
| 39 | GO:0003677;  GO:0003700;  GO:0006355 | MF_DNA binding;  MF_DNA-binding transcription factor activity;  BP_Regulation of transcription, DNA-templated | NP_001235127.1 | Uncharacterized LOC100527371 | 2.188078 | 0.010562 |
| 40 | N.A. | N.A. | XP_025982175.1 | Zinc finger protein 4-like | 2.179639 | 0.020045 |
| 41 | GO:0046983 | MF_ Protein dimerization activity | NP_001235904.1 | Uncharacterized LOC100500546 | 2.173903 | 0.031622 |
| 42 | GO:0016020;  GO:0016798 | CC_Membrane;  MF_Hydrolase activity, acting on glycosyl bonds | NP_001241969.1 | Heparanase-like protein 3-like | 2.140568 | 0.049548 |
| 43 | N.A. | N.A. | NP_001235729.2 | Uncharacterized LOC100306230 | 2.134595 | 0.015298 |
| 44 | GO:0008113;  GO:0055114 | MF_Peptide-methionine (S)-S-oxide reductase activity;  BP_Oxidation-reduction process | NP_001238470.1 | Uncharacterized LOC100527085 | 2.120518 | 0.0279 |
| 45 | GO:0004553;  GO:0005975 | MF_Hydrolase activity, hydrolyzing O-glycosyl compounds;  BP_Carbohydrate metabolic process | XP_006577900.1 | Anti-sigma-I factor rsgi6 | 2.100793 | 0.009927 |
| 46 | GO:0000287;  GO:0016831;  GO:0030976 | MF_Magnesium ion binding;  MF_Carboxy-lyase activity;  MF_Thiamine pyrophosphate binding | XP_003529168.1 | Pyruvate decarboxylase 2 | 2.092000 | 0.011914 |
| 47 | GO:0030599;  GO:0042545 | MF_Pectinesterase activity;  BP_Cell wall modification | XP_003516527.2 | Probable pectinesterase 53 | 2.085781 | 0.032576 |
| 48 | GO:0008171;  GO:0046983 | MF_O-methyltransferase activity;  MF_Protein dimerization activity | XP_003557042.1 | Probable O-methyltransferase 3 | 2.057006 | 0.037624 |
| 49 | GO:0016021 | CC_ Integral component of membrane | NP_001237564.1 | Uncharacterized LOC100305590 | 2.038793 | 0.017991 |
| 50 | GO:0016788 | MF_Hydrolase activity, acting on ester bonds | XP_025979902.1 | GDSL esterase/lipase At1g54790 | 2.019422 | 0.011968 |
| 51 | N.A. | N.A. | . | Small nucleolar RNA snor97 | 2.011056 | 0.036257 |
| 52 | GO:0030410;  GO:0030418 | MF_Nicotianamine synthase activity;  BP_Nicotianamine biosynthetic process | NP_001304637.2 | Nicotianamine synthase-like protein | 1.994226 | 0.044579 |
| 53 | GO:0030599;  GO:0042545 | MF_Pectinesterase activity;  BP_Cell wall modification | XP_003530551.1 | Probable pectinesterase 53 | 1.993849 | 0.034207 |
| 54 | GO:0016702;  GO:0055114 | MF_Oxidoreductase activity, acting on single donors with incorporation of molecular oxygen, incorporation of two atoms of oxygen;  BP_Oxidation-reduction process | XP_003543744.1 | Plant cysteine oxidase 2 | 1.950800 | 0.008225 |
| 55 | GO:0005509 | MF_ Calcium ion binding | XP_003531806.1 | Uncharacterized LOC100792416 | 1.942425 | 0.038528 |
| 56 | GO:0005634;  GO:0005829;  GO:0009691;  GO:0016799 | CC_Nucleus;  CC_Cytosol;  BP_Cytokinin biosynthetic process;  MF_Hydrolase activity, hydrolyzing N-glycosyl compounds | NP_001241594.1 | Cytokinin riboside 5'-monophosphate phosphoribohydrolase LOG1-like | 1.940066 | 0.044202 |
| 57 | GO:0005515 | MF_Protein binding | XP_025982728.1 | Kelch repeat-containing protein | 1.932361 | 0.022016 |
| 58 | GO:0046983 | MF_Protein dimerization activity | NP_001241949.1 | Transcription factor bhlh93-like | 1.909537 | 0.012602 |
| 59 | GO:0003677;  GO:0003700;  GO:0006355 | MF_DNA binding;  MF_DNA-binding transcription factor activity;  BP_Regulation of transcription, DNA-templated | NP_001241446.1 | Ethylene-responsive transcription factor RAP2-2-like | 1.906566 | 0.002088 |
| ***60 | GO:0005506;  GO:0016705;  GO:0020037;  GO:0055114 | MF_Iron ion binding;  MF_Oxidoreductase activity, acting on paired donors, with incorporation or reduction of molecular oxygen;  MF_Heme binding;  BP_Oxidation-reduction process | XP_003552729.2 | Cytokinin hydroxylase | 1.903193 | 0.00778 |
| 61 | GO:0004553;  GO:0005618;  GO:0010411;  GO:0016762;  GO:0042546;  GO:0048046 | MF_Hydrolase activity, hydrolyzing O-glycosyl compounds;  CC_Cell wall;  BP_Xyloglucan metabolic process;  MF_Xyloglucan:xyloglucosyl transferase activity;  BP_Cell wall biogenesis;  CC_Apoplast | NP_001241974.1 | Probable xyloglucan endotransglucosylase/hydrolase protein 32-like | 1.872985 | 0.008809 |
| 62 | GO:0004459;  GO:0005737;  GO:0005975;  GO:0019752;  GO:0055114 | MF_L-lactate dehydrogenase activity;  CC_Cytoplasm;  BP_Carbohydrate metabolic process;  BP_Carboxylic acid metabolic process;  BP_Oxidation-reduction process | XP_003525821.2 | L-lactate dehydrogenase B | 1.858467 | 0.008001 |
| 63 | GO:0005506;  GO:0016705;  GO:0020037;  GO:0055114 | MF_Iron ion binding;  MF_Oxidoreductase activity, acting on paired donors, with incorporation or reduction of molecular oxygen;  MF_Heme binding;  BP_Oxidation-reduction process | NP_001240107.1 | Cytochrome P450 71D11-like | 1.852190 | 0.033317 |
| 64 | GO:0000272;  GO:0016161 | BP_Polysaccharide catabolic process;  MF_Beta-amylase activity | XP_003539882.1 | Beta-amylase | 1.850917 | 0.044513 |
| 65 | N.A. | N.A. | XP_003527454.1 | Uncharacterized LOC100817911 | 1.846557 | 0.024442 |
| 66 | GO:0003950 | MF_NAD+ ADP-ribosyltransferase activity | XP_003523079.1 | Probable inactive poly [ADP-ribose] polymerase SRO5 | 1.843342 | 0.04385 |
| 67 | GO:0005507;  GO:0016491;  GO:0055114 | MF_Copper ion binding;  MF_Oxidoreductase activity;  BP_Oxidation-reduction process | XP_006592035.1 | L-ascorbate oxidase homolog | 1.830352 | 0.030677 |
| 68 | GO:0006006;  GO:0016620;  GO:0050661;  GO:0051287;  GO:0055114 | BP_Glucose metabolic process;  MF_Oxidoreductase activity, acting on the aldehyde or oxo group of donors, NAD or NADP as acceptor;  MF_NADP binding;  MF_NAD binding;  BP_Oxidation-reduction process | NP_001240046.1 | Glyceraldehyde-3-phosphate dehydrogenase, cytosolic-like | 1.828235 | 0.038325 |
| 69 | N.A. | N.A. | XP_006598835.1 | Uncharacterized LOC102670094 | 1.826444 | 0.030543 |
| 70 | GO:0004190;  GO:0006508 | MF_Aspartic-type endopeptidase activity;  BP_Proteolysis | XP_003522749.1 | Aspartyl protease AED3 | 1.822801 | 0.037213 |
| 71 | GO:0004857;  GO:0030599;  GO:0042545 | MF_Enzyme inhibitor activity;  MF_Pectinesterase activity;  BP_Cell wall modification | XP_006578909.1 | Probable pectinesterase/pectinesterase inhibitor 61 | 1.816705 | 0.046416 |
| 72 | GO:0004462 | MF_Lactoylglutathione lyase activity | NP_001237480.1 | Glyoxalase GLYI-13 | 1.813645 | 0.028369 |
| 73 | N.A. | N.A. | . | Small nucleolar RNA u31b | 1.811301 | 0.019759 |
| 74 | N.A. | N.A. | XP_003523448.1 | Uncharacterized LOC100810043 | 1.807688 | 0.010751 |
| 75 | GO:0004185;  GO:0006508 | MF_Serine-type carboxypeptidase activity;  BP_Proteolysis | XP_003527966.2 | Serine carboxypeptidase-like 42 | 1.801755 | 0.043868 |
| 76 | GO:0004190;  GO:0006508 | MF_Aspartic-type endopeptidase activity;  BP_Proteolysis | XP_003546907.1 | Aspartyl protease family protein At5g10770 | 1.800607 | 0.025327 |
| 77 | N.A. | N.A. | XP_003530903.1 | Actin cytoskeleton-regulatory complex protein PAN1 | 1.798472 | 0.021491 |
| 78 | GO:0003677;  GO:0003700;  GO:0006355 | MF_DNA binding;  MF_DNA-binding transcription factor activity;  BP_Regulation of transcription, DNA-templated | XP_014624311.1 | Ethylene-responsive transcription factor LEP | 1.786967 | 0.01865 |
| 79 | GO:0003677 | MF_DNA binding | XP_003520640.1 | Protein REVEILLE 8 | 1.775460 | 0.041306 |
| 80 | GO:0003677;  GO:0003700;  GO:0006355 | MF_DNA binding;  MF_DNA-binding transcription factor activity;  BP_Regulation of transcription, DNA-templated | XP_003555421.2 | Ethylene-responsive transcription factor LEP | 1.767987 | 0.023897 |
| 81 | GO:0009733 | BP_Response to auxin | XP_003547854.1 | Auxin-responsive protein SAUR78 | 1.764686 | 0.049372 |
| 82 | GO:0005515 | MF_Protein binding | XP_003532194.1 | BTB/POZ domain-containing protein At1g55760 | 1.763031 | 0.003128 |
| 83 | GO:0006520;  GO:0008483;  GO:0009058;  GO:0030170 | BP_Cellular amino acid metabolic process;  MF_Transaminase activity;  BP_Biosynthetic process;  MF_Pyridoxal phosphate binding | XP_003517004.1 | Aspartate aminotransferase, mitochondrial | 1.756921 | 0.009636 |
| 84 | GO:0004857 | MF_Enzyme inhibitor activity | XP_003541070.3 | 21 kda protein | 1.745708 | 0.00259 |
| 85 | GO:0004672;  GO:0005515;  GO:0005524;  GO:0006468 | MF_Protein kinase activity;  MF_Protein binding;  MF_ATP binding;  BP_Protein phosphorylation | XP_006598006.1 | Probable inactive leucine-rich repeat receptor-like protein kinase  At3g03770 | 1.735650 | 0.010418 |
| 86 | GO:0016021 | CC_ Integral component of membrane | XP_003551861.1 | Leucine-rich repeat extensin-like protein 6 | 1.730637 | 0.021121 |
| 87 | GO:0030247 | MF_Polysaccharide binding | XP_003550483.1 | Wall-associated receptor kinase 2 | 1.720704 | 0.002083 |
| 88 | N.A. | N.A. | . | Uncharacterized LOC100305610 | 1.719138 | 0.030878 |
| 89 | GO:0016021 | CC_Integral component of membrane | XP_014634191.1 | Uncharacterized GPI-anchored protein At4g28100 | 1.718001 | 0.01959 |
| 90 | GO:0008033  GO:0052381  GO:0009691  GO:0006400  GO:0005739 | BP_tRNA processing  MF_tRNA dimethylallytransferase activity  BP_cytokinin biosynthetic process  BP_tRNA modification  CC_mitochondrion | XP_003528670.1 | Adenylate isopentenyltransferase 5, chloroplastic | 1.716814 | 0.045672 |
| 91 | GO:0004252;  GO:0006508 | MF_Serine-type endopeptidase activity;  BP_Proteolysis | XP_003541310.1 | Subtilisin-like protease SBT1.8 | 1.709421 | 0.013124 |
| 92 | N.A. | N.A. | XP_014617328.1 | Uncharacterized LOC100779669 | 1.683306 | 0.01063 |
| 93 | GO:0046856 | BP_Phosphatidylinositol dephosphorylation | XP_003541391.1 | Type I inositol polyphosphate 5-phosphatase 8 | 1.669646 | 0.026034 |
| 94 | GO:0004672;  GO:0005509;  GO:0005524;  GO:0006468 | MF_Protein kinase activity;  MF_Calcium ion binding;  MF_ATP binding;  BP_Protein phosphorylation | XP_003523190.1 | Calcium-dependent protein kinase SK5-like | 1.669380 | 0.01684 |
| 95 | GO:0004672;  GO:0005515;  GO:0005524;  GO:0006468 | MF_Protein kinase activity;  MF_Protein binding;  MF_ATP binding;  BP_Protein phosphorylation | XP_003552656.1 | Probably inactive leucine-rich repeat receptor-like protein kinase At3g28040 | 1.663182 | 0.047268 |
| 96 | N.A. | N.A. | XP_003543359.1 | Uncharacterized LOC100305771 | 1.659252 | 0.045991 |
| 97 | N.A. | N.A. | XP_003534839.1 | Uncharacterized LOC100786266 | 1.657233 | 0.017855 |
| 98 | N.A. | N.A. | XP_003537793.1 | BTB/POZ domain-containing protein At1g50280 | 1.657024 | 0.032808 |
| 99 | GO:0005094;  GO:0005737 | MF_Rho GDP-dissociation inhibitor activity;  CC_Cytoplasm | NP_001241061.1 | Rho GDP-dissociation inhibitor 1-like | 1.641849 | 0.006862 |
| 100 | GO:0004857;  GO:0030599;  GO:0042545 | MF_Enzyme inhibitor activity;  MF_Pectinesterase activity;  BP_Cell wall modification | XP_003519757.1 | Pectinesterase | 1.637839 | 0.032664 |
| 101 | N.A. | N.A. | XP_025984683.1 | Uncharacterized LOC100813529 | 1.637346 | 0.048562 |
| 102 | GO:0000287;  GO:0016831;  GO:0030976 | MF_Magnesium ion binding;  MF_Carboxy-lyase activity;  MF_Thiamine pyrophosphate binding | XP_014621278.1 | Pyruvate decarboxylase 1 | 1.636941 | 0.045063 |
| 103 | GO:0003677;  GO:0005634;  GO:0045893 | MF_DNA binding;  CC_Nucleus;  BP_Positive regulation of transcription, DNA-templated | XP_006578434.1 | Protein LATERAL ROOT PRIMORDIUM 1 | 1.632886 | 0.010434 |
| 104 | GO:0004252;  GO:0006508 | MF_Serine-type endopeptidase activity;  BP_Proteolysis | XP_006606084.1 | Subtilisin-like protease SBT1.3 | 1.632289 | 0.003703 |
| 105 | N.A. | N.A. | XP_003536843.1 | Uncharacterized LOC100813226 | 1.632207 | 0.022004 |
| 106 | GO:0016021 | CC­_ Integral component of membrane | XP_006596619.1 | Uncharacterized LOC102665063 | 1.630543 | 0.027964 |
| 107 | GO:0016021 | CC­_ Integral component of membrane | NP_001242265.2 | Uncharacterized LOC100798817 | 1.624838 | 0.026182 |
| 108 | N.A. | N.A. | NP_001341001.1 | LBD domain-containing transcription factor | 1.624746 | 0.048369 |
| 109 | GO:0003677;  GO:0003700;  GO:0006355 | MF_DNA binding;  MF_DNA-binding transcription factor activity;  BP_Regulation of transcription, DNA-templated | NP_001240238.1 | Dehydration-responsive element-binding protein 2C-like | 1.624043 | 0.017832 |
| 110 | N.A. | N.A. | . | . | 1.621719 | 0.014535 |
| 111 | GO:0003676 | MF_Nucleic acid binding | XP_003541979.1 | Protein SENSITIVE TO PROTON RHIZOTOXICITY 2 | 1.613492 | 0.046823 |
| 112 | GO:0016614;  GO:0050660;  GO:0055114 | MF_Oxidoreductase activity, acting on CH-OH group of donors;  MF_Flavin adenine dinucleotide binding;  BP_Oxidation-reduction process | XP_003517995.2 | (R)-mandelonitrile lyase-like | 1.612646 | 0.039156 |
| 113 | GO:0016021 | CC_Integral component of membrane | XP_014634156.1 | Probable protein ABIL5 | 1.611875 | 0.021559 |
| 114 | GO:0003680 | MF_AT DNA binding | XP_006579618.1 | AT-hook motif nuclear-localized protein 24 | 1.602557 | 0.04097 |
| 115 | GO:0005634;  GO:0009416 | CC_Nucleus;  BP_Response to light stimulus | XP_025980520.1 | Protein LIGHT-DEPENDENT SHORT HYPOCOTYLS 10 | 1.592846 | 0.033667 |
| 116 | GO:0046983 | MF_Protein dimerization activity | XP_003544814.1 | Transcription factor bhlh96 | 1.592295 | 0.015255 |
| 117 | GO:0003700;  GO:0005634;  GO:0006355;  GO:0043565 | MF_DNA-binding transcription factor activity;  CC_Nucleus;  BP_Regulation of transcription, DNA-templated;  MF_Sequence-specific DNA binding | XP_003523945.1 | Heat stress transcription factor B-4 | 1.592258 | 0.025858 |
| 118 | GO:0006355;  GO:0008270;  GO:0043565 | BP_Regulation of transcription, DNA-templated;  MF_zinc ion binding;  MF_sequence-specific DNA binding | XP_003521089.1 | GATA transcription factor 2 | 1.590258 | 0.045873 |
| 119 | N.A. | N.A. | XP_003529661.1 | Major latex allergen Hev b 5 | 1.588887 | 0.043294 |
| 120 | N.A. | N.A. | XP_006602703.1 | Uncharacterized LOC100788775 | 1.586699 | 0.013315 |
| 121 | GO:0008270  GO:0016020  GO:0046872  GO:0016021 | MF_Zinc ion binding  CC_Membrane  MF_Metal ion binding  CC_Integral component of membrane | XP_014621857.1 | E3 ubiquitin-protein ligase MARCH7 | 1.583935 | 0.014432 |
| 122 | GO:0004497;  GO:0009536;  GO:0009853;  GO:0015977;  GO:0015979;  GO:0016984;  GO:0055114 | MF_Monooxygenase activity;  CC_Plastid;  BP_Photorespiration;  BP_Carbon fixation;  BP_Photosynthesis;  MF_Ribulose-bisphosphate carboxylase activity;  BP_Oxidation-reduction process | NP_001235868.2 | Putative ribulose bisphosphate carboxylase small chain | 1.581513 | 0.034476 |
| 123 | N.A. | N.A. | XP_003533742.1 | Protein At-4/1 | 1.580356 | 0.020186 |
| 124 | GO:0004857;  GO:0030599;  GO:0042545 | MF_Enzyme inhibitor activity;  MF_Pectinesterase activity;  BP_Cell wall modification | XP_006604725.1 | Probable pectinesterase/pectinesterase inhibitor 20 | 1.575315 | 0.044468 |
| 125 | GO:0004650;  GO:0005975 | MF_Polygalacturonase activity;  BP_Carbohydrate metabolic process | XP_003545367.1 | Probable polygalacturonase | 1.574124 | 0.037363 |
| 126 | GO:0005524 | MF_ATP binding | XP_003549010.1 | Atpase family AAA domain-containing protein 1-A | 1.571387 | 0.036113 |
| 127 | GO:0003677 | MF_DNA binding | XP_006587217.1 | MYB-CC domain-containing transcription factor PHR15 | 1.570347 | 0.047718 |
| 128 | N.A. | N.A. | XP_003551651.1 | Uncharacterized LOC100818003 | 1.569092 | 0.046238 |
| 129 | GO:0006486;  GO:0016757 | BP_Protein glycosylation;  MF_Transferase activity, transferring glycosyl groups | XP_003535253.1 | Probable glycosyltransferase At5g03795 | 1.560854 | 0.038459 |
| 130 | GO:0004190;  GO:0006508 | MF_Aspartic-type endopeptidase activity;  BP_Proteolysis | XP_003531753.2 | Aspartyl protease family protein At5g10770 | 1.555780 | 0.038081 |
| 131 | N.A. | N.A. | XP_003555129.1 | LOB domain-containing protein 41 | 1.554678 | 0.012958 |
| 132 | GO:0003924;  GO:0005525;  GO:0007264 | MF_GTPase activity;  MF_GTP binding;  BP_Small GTPase mediated signal transduction | XP_003543315.1 | Rac-like GTP-binding protein RAC2 | 1.549587 | 0.035115 |
| 133 | GO:0005515 | MF_Protein binding | XP_003530183.1 | Leucine-rich repeat extensin-like protein 6 | 1.546808 | 0.009567 |
| 134 | GO:0015297;  GO:0016020;  GO:0042910;  GO:0055085 | MF_Antiporter activity;  CC_Membrane;  MF_xenobiotic transmembrane transporter activity;  BP_Transmembrane transport | XP_003526583.2 | Protein DETOXIFICATION 12 | 1.546806 | 0.008217 |
| 135 | N.A. | N.A. | XP_006604871.1 | Uncharacterized LOC100777580 | 1.542259 | 0.013621 |
| 136 | GO:0016021 | CC_Integral component of membrane | XP_006589133.1 | Formin-like protein 11 | 1.538807 | 0.020743 |
| 137 | GO:0005634;  GO:0009416 | CC_Nucleus;  BP_Response to light stimulus | XP_003553762.1 | Protein LIGHT-DEPENDENT SHORT HYPOCOTYLS 4 | 1.534536 | 0.010215 |
| 138 | GO:0004672;  GO:0005524;  GO:0006468 | MF_Protein kinase activity;  MF_ATP binding;  BP_Protein phosphorylation | XP_014624950.1 | Casein kinase 1-like protein 3 | 1.533564 | 0.030718 |
| 139 | GO:0005452;  GO:0006820;  GO:0016021 | MF_Inorganic anion exchanger activity;  BP_Anion transport;  CC_Integral component of membrane | XP_003554567.1 | Boron transporter 1 | 1.527527 | 0.023059 |
| 140 | N.A. | N.A. | XP_014631701.1 | Uncharacterized LOC100812576 | 1.526788 | 0.024508 |
| 141 | GO:0005506;  GO:0016705;  GO:0020037;  GO:0055114 | MF_Iron ion binding;  MF_Oxidoreductase activity, acting on paired donors, with incorporation or reduction of molecular oxygen;  MF_Heme binding;  BP_Oxidation-reduction process | XP_014625807.1 | Cytochrome P450 90A1 | 1.526557 | 0.028077 |
| 142 | GO:0003887;  GO:0016021;  GO:0016567;  GO:0071897 | MF_DNA-directed DNA polymerase activity;  CC_Integral component of membrane;  BP_Protein ubiquitination;  BP_DNA biosynthetic process | XP_003552944.1 | RING-H2 finger protein ATL63 | 1.525996 | 0.033572 |
| 143 | GO:0016758 | MF_Transferase activity, transferring hexosyl groups | XP_003516842.1 | UDP-glycosyltransferase 83A1 | 1.523369 | 0.03448 |
| 144 | XP_003542798.1 | MF_ Sulfotransferase activity | XP_003542798.1 | Cytosolic sulfotransferase 15 | 1.522296 | 0.030801 |
| 145 | GO:0030001;  GO:0046872 | BP_Metal ion transport;  MF_Metal ion binding | XP_003541886.1 | Heavy metal-associated isoprenylated plant protein 28 | 1.521221 | 0.046531 |
| 146 | GO:0005515;  GO:0005634;  GO:0006355 | MF_Protein binding;  CC_Nucleus;  BP_Regulation of transcription, DNA-templated | NP_001237849.1 | Auxin-regulated protein (Aux22) | 1.519987 | 0.038129 |
| 147 | GO:0000287;  GO:0004743;  GO:0006096;  GO:0030955 | MF_Magnesium ion binding;  MF_Pyruvate kinase activity;  BP_Glycolytic process;  MF_Potassium ion binding | XP_006606294.1 | Pyruvate kinase 1, cytosolic | 1.519623 | 0.024349 |
| 148 | GO:0046983 | MF_ Protein dimerization activity | XP_006572984.1 | Transcription factor bhlh93 | 1.518246 | 0.027397 |
| 149 | GO:0003676 | MF_ Nucleic acid binding | XP_006583685.1 | Protein indeterminate-domain 5, chloroplastic | 1.517266 | 0.035632 |
| 150 | GO:0003924;  GO:0005200;  GO:0005525;  GO:0005874;  GO:0007017 | MF_GTPase activity;  MF_Structural constituent of cytoskeleton;  MF_GTP binding;  CC_Microtubule;  BP_Microtubule-based process | XP_003517369.1 | Tubulin alpha-2 chain | 1.516850 | 0.041191 |
| 151 | GO:0005515 | MF­_protein binding | XP_003544257.1 | Uncharacterized LOC100792391 | 1.513678 | 0.007555 |
| 152 | GO:0016620;  GO:0055114 | MF_Oxidoreductase activity, acting on the aldehyde or oxo group of donors, NAD or NADP as acceptor;  BP_Oxidation-reduction process | XP_003534191.1 | Aldehyde dehydrogenase family 2 member C4 | 1.505845 | 0.038187 |
| 153 | N.A. | N.A. | NP_001236822.1 | Uncharacterized LOC100527749 | 1.501723 | 0.044401 |

^a-b^N.A. means Not Annotated by Blast2GO software^8^ or QuickGO (https://www.ebi.ac.uk/QuickGO/).

^b^BP means Biological Process; MF means Molecular Function; and CC means Component Cell.

^c^RNAseq fold changes in the roots of En1282 inoculated with USDA61 versus the roots of En1282 inoculated with *bel2-5* deletion mutant.

***Representative DEGs were validated by qRT-PCR (Supplementary Figure 2).

**Supplementary Table 3.** The down-regulated DEGs in *Glycine max* during interaction with *B. elkanii* wild-type as compared with *bel2*-5 deletion mutant

| No | GO ID annotation^a^ | Classification^b^ | ID | Similarity | Fold change^c^ | *P*-value |
| --- | --- | --- | --- | --- | --- | --- |
| 1 | GO:0005506;  GO:0016021;  GO:0016709;  GO:0020037;  GO:0055114 | MF_Iron ion binding;  CC_Integral component of membrane;  MF_Oxidoreductase activity, acting on paired donors, with incorporation or reduction of molecular oxygen, NAD(P)H as one donor, and incorporation of one atom of oxygen;  MF_Heme binding;  BP_Oxidation-reduction process | NP_001304412.2 | Cytochrome P450 71D8 | 0.139903 | 0.014660148 |
| 2 | GO:0009695;  GO:0016021;  GO:0046423;  GO:0048046 | BP_Jasmonic acid biosynthetic process;  CC_Integral component of membrane;  MF_Allene-oxide cyclase activity;  CC_Apoplast | XP_003554228.1 | Dirigent protein 1 | 0.158461 | 0.016089169 |
| 3 | GO:0009507;  GO:0016021;  GO:0016765 | CC_Chloroplast;  CC_Integral component of membrane;  MF_Transferase activity, transferring alkyl or aryl (other than methyl) groups | NP_001335591.1 | Homogentisate phytyltransferase 1, chloroplastic-like | 0.168294 | 0.029404276 |
| 4 | GO:0005506;  GO:0016021;  GO:0016709;  GO:0020037;  GO:0055114 | MF_Iron ion binding;  CC_Integral component of membrane;  MF_Oxidoreductase activity, acting on paired donors, with incorporation or reduction of molecular oxygen, NAD(P)H as one donor, and incorporation of one atom of oxygen;  MF_Heme binding;  BP_Oxidation-reduction process | NP_001240077.1 | Cytochrome p450 82A2-like | 0.173142 | 0.047099226 |
| 5 | GO:0003677;  GO:0005634;  GO:0006355 | MF_DNA binding;  CC_Nucleus;  BP_Regulation of transcription, DNA-templated | NP_001238234.2 | NAC domain protein nac3 | 0.177284 | 0.048584508 |
| 6 | GO:0005506;  GO:0016021;  GO:0016709;  GO:0020037;  GO:0055114 | MF_Iron ion binding;  CC_Integral component of membrane;  MF_Oxidoreductase activity, acting on paired donors, with incorporation or reduction of molecular oxygen, NAD(P)H as one donor, and incorporation of one atom of oxygen;  MF_Heme binding;  BP_Oxidation-reduction process | NP_001241120.1 | Isoflavone 2'-hydroxylase-like | 0.181093 | 0.033383487 |
| 7 | GO:0005506;  GO:0016021;  GO:0016709;  GO:0020037;  GO:0055114 | MF_Iron ion binding;  CC_Integral component of membrane;  MF_Oxidoreductase activity, acting on paired donors, with incorporation or reduction of molecular oxygen, NAD(P)H as one donor, and incorporation of one atom of oxygen;  MF_Heme binding;  BP_Oxidation-reduction process | XP_003537457.1 | Cytochrome p450 71d8-like | 0.194202 | 0.010549975 |
| 8 | GO:0005351;  GO:0005887;  GO:0034219;  GO:1902600 | MF_Carbohydrate:proton symporter activity;  CC_Integral component of plasma membrane;  BP_Carbohydrate transmembrane transport;  BP_Proton transmembrane transport | XP_003517581.1 | Sugar transport protein 13 | 0.194448 | 0.0479218 |
| 9 | GO:0009695;  GO:0016021;  GO:0046423;  GO:0048046 | BP_Jasmonic acid biosynthetic process;  CC_Integral component of membrane;  MF_Allene-oxide cyclase activity;  CC_Apoplast | NP_001236934.2 | Dirigent-like protein | 0.207210 | 0.005388034 |
| ***10 | GO:0009815;  GO:0045486;  GO:0046872;  GO:0055114 | MF_1-aminocyclopropane-1-carboxylate oxidase activity;  MF_Naringenin 3-dioxygenase activity;  MF_Metal ion binding;  BP_Oxidation-reduction process | NP_001341820.1 | Putative 1-aminocyclopropane-1-carboxylate oxidase | 0.214690 | 0.024842474 |
| 11 | GO:0009116;  GO:0016021;  GO:0016798 | BP_Nucleoside metabolic process;  CC_Integral component of membrane;  MF_Hydrolase activity, acting on glycosyl bonds | XP_003528930.1 | Bark storage protein A | 0.219269 | 0.043190033 |
| 12 | GO:0004864;  GO:0005634;  GO:0005737;  GO:0006952;  GO:0009607;  GO:0009738;  GO:0010427;  GO:0030001;  GO:0032515;  GO:0038023;  GO:0046872;  GO:0080163 | MF_Protein phosphatase inhibitor activity;  CC_Nucleus;  CC_Cytoplasm;  BP_Defense response;  BP_Response to biotic stimulus;  BP_Abscisic acid-activated signaling pathway;  MF_Abscisic acid binding;  BP_Metal ion transport;  BP_Negative regulation of phosphoprotein phosphatase activity;  MF_Signaling receptor activity;  MF_Metal ion binding;  BP_Regulation of protein serine/threonine phosphatase activity | NP_001237916.2 | SRPBCC domain-containing protein | 0.220490 | 0.036943418 |
| 13 | GO:0004197;  GO:0005773;  GO:0006624;  GO:0051603 | MF_Cysteine-type endopeptidase activity;  CC_Vacuole;  BP_Vacuolar protein processing;  BP_Proteolysis involved in cellular protein catabolic process | NP_001236564.1 | Vacuolar processing enzyme 2 | 0.236206 | 0.038627102 |
| 14 | GO:0005887;  GO:0008271;  GO:0015301;  GO:1902358 | CC_Integral component of plasma membrane;  MF_Secondary active sulfate transmembrane  MF_Transporter activity;  anion:anion antiporter activity;  BP_Sulfate transmembrane transport | XP_003538517.1 | Sulfate transporter 2.1 | 0.272832 | 0.045037134 |
| 15 | Unknown | Unknown | NP_001304579.2 | Uncharacterized LOC100788426 | 0.284254 | 0.011879181 |
| 16 | Unknown | Unknown | NP_001235359.1 | Uncharacterized LOC100500507 | 0.287866 | 0.017629225 |
| 17 | GO:0004014;  GO:0005829;  GO:0006557;  GO:0006597;  GO:0008295;  GO:0016021 | MF_Adenosylmethionine decarboxylase activity;  CC_Cytosol;  BP_S-adenosylmethioninamine biosynthetic process;  BP_Spermine biosynthetic process;  BP_Spermidine biosynthetic process;  CC_Integral component of membrane | XP_006585827.1 | S-adenosylmethionine decarboxylase proenzyme | 0.290418 | 0.039383212 |
| 18 | GO:0009815;  GO:0045486;  GO:0046872;  GO:0055114 | MF_1-aminocyclopropane-1-carboxylate oxidase activity;  MF_Naringenin 3-dioxygenase activity;  MF_Metal ion binding;  BP_oxidation-reduction process | XP_003519448.1 | 1-aminocyclopropane-1-carboxylate oxidase | 0.309084 | 0.016727065 |
| 19 | GO:0005506;  GO:0006952;  GO:0016021;  GO:0020037;  GO:0047082;  GO:0055114 | MF_Iron ion binding;  BP_Defense response;  CC_Integral component of membrane;  MF_Heme binding;  MF_3,9-dihydroxypterocarpan 6a-monooxygenase activity;  BP_Oxidation-reduction process | NP_001241186.1 | Cytochrome P450 93A1-like | 0.309933 | 0.026159092 |
| 20 | GO:0004601;  GO:0005576;  GO:0006979;  GO:0020037;  GO:0042744;  GO:0046872;  GO:0055114;  GO:0098869 | MF_Peroxidase activity;  CC_Extracellular region;  BP_Response to oxidative stress;  MF_Heme binding;  BP_Hydrogen peroxide catabolic process;  MF_Metal ion binding;  BP_Oxidation-reduction process;  BP_Cellular oxidant detoxification | NP_001241914.1 | Peroxidase 52-like | 0.312496 | 0.033466258 |
| 21 | GO:0003712;  GO:0005634;  GO:0005737;  GO:0009910;  GO:0010228;  GO:0016021;  GO:1903506 | MF_Transcription coregulator activity;  CC_Nucleus;  CC_Cytoplasm;  BP_Negative regulation of flower development;  BP_Vegetative to reproductive phase transition of meristem;  CC_Integral component of membrane;  BP_Regulation of nucleic acid-templated transcription | NP_001241437.1 | Putative phosphatidylethanolamine-binding protein TFL1a | 0.317200 | 0.0059459 |
| 22 | GO:0016829;  GO:0030246 | MF_Lyase activity;  MF_Carbohydrate binding | NP_001235692.1 | 24 kDa seed coat protein | 0.318022 | 0.040594331 |
| 23 | GO:0005506;  GO:0006952;  GO:0016021;  GO:0020037;  GO:0047082;  GO:0055114 | MF_Iron ion binding;  BP_Defense response;  CC_Integral component of membrane;  MF_Heme binding;  MF_3,9-dihydroxypterocarpan 6a-monooxygenase activity;  BP_Oxidation-reduction process | XP_006604384.1 | 3,9-dihydroxypterocarpan 6A-monooxygenase | 0.322981 | 0.036223288 |
| 24 | GO:0030001;  GO:0046872 | BP_Metal ion transport;  MF_Metal ion binding | NP_001235140.2 | Heavy-metal-associated domain-containing protein | 0.334715 | 0.011715198 |
| 25 | GO:0048046 | CC_ Apoplast | NP_001235010.1 | Dirigent-like protein | 0.338178 | 0.04819886 |
| 26 | Unknown | Unknown | XP_003526429.1 | Uncharacterized LOC100805091 | 0.343508 | 0.044809216 |
| ***27 | GO:0003677;  GO:0003700;  GO:0005634;  GO:0006355 | MF_DNA binding;  MF_DNA-binding transcription factor activity;  CC_Nucleus;  BP_Regulation of transcription, DNA-templated | NP_001241134.1 | Ethylene-responsive transcription factor ERF098-like protein | 0.344057 | 0.037975441 |
| 28 | GO:0004364;  GO:0005315;  GO:0009651;  GO:0031305;  GO:0035435 | MF_Glutathione transferase activity;  MF_Inorganic phosphate transmembrane transporter activity;  BP_Response to salt stress;  CC_Integral component of mitochondrial inner membrane;  BP_Phosphate ion transmembrane transport | XP_003548029.1 | Mitochondrial phosphate carrier protein 3, mitochondrial | 0.346698 | 0.038839513 |
| 29 | GO:0009813;  GO:0016210 | BP_Flavonoid biosynthetic process;  MF_Naringenin-chalcone synthase activity | NP_001337038.1 | Chalcone synthase 1 | 0.352054 | 0.038818782 |
| 30 | Unknown | Unknown | NP_001235581.1 | Uncharacterized LOC100305963 | 0.353588 | 0.021898407 |
| 31 | GO:0098869 | BP_ Cellular oxidant detoxification | XP_003544272.1 | Protein RESPONSE TO LOW SULFUR 3 | 0.354513 | 0.042991791 |
| 32 | GO:0003700;  GO:0005634;  GO:0006355;  GO:0043565 | MF_DNA-binding transcription factor activity;  CC_Nucleus;  BP_Regulation of transcription, DNA-templated;  MF_Sequence-specific DNA binding | XP_003549123.1 | Probable WRKY transcription factor 75 | 0.358900 | 0.042736427 |
| 33 | Unknown | Unknown | XP_006591566.1 | Extensin-2-like | 0.359517 | 0.033430075 |
| 34 | GO:0005506;  GO:0016021;  GO:0016709;  GO:0020037;  GO:0055114 | MF_Iron ion binding;  CC_Integral component of membrane;  MF_Oxidoreductase activity, acting on paired donors, with incorporation or reduction of molecular oxygen, NAD(P)H as one donor, and incorporation of one atom of oxygen;  MF_Heme binding;  BP_Oxidation-reduction process | XP_003548205.1 | Cytochrome P450 71A26 | 0.362300 | 0.021880263 |
| 35 | GO:0008483;  GO:0009058;  GO:0030170 | MF_Transaminase activity;  BP_Biosynthetic process;  MF_Pyridoxal phosphate binding | NP_001242772.2 | Aminotransferase superfamily protein | 0.372318 | 0.02527577 |
| 36 | GO:0016021 | CC_integral component of membrane | XP_003536091.1 | E3 ubiquitin-protein ligase ATL6 | 0.383506 | 0.032443696 |
| 37 | GO:0005506;  GO:0016021;  GO:0016709;  GO:0020037;  GO:0055114 | MF_Iron ion binding;  CC_Integral component of membrane;  MF_Oxidoreductase activity, acting on paired donors, with incorporation or reduction of molecular oxygen, NAD(P)H as one donor, and incorporation of one atom of oxygen;  MF_Heme binding;  BP_Oxidation-reduction process | NP_001240972.1 | Cytochrome P450 82A3-like | 0.388253 | 0.00951257 |
| 38 | N.A. | N.A. | NP_001276127.1 | Isoflavone reductase-like | 0.392236 | 0.033815249 |
| 39 | GO:0016020 | CC_ Membrane | XP_003541868.1 | Uncharacterized LOC100779759 | 0.398431 | 0.041306493 |
| 40 | N.A. | N.A. | NP_001341294.1 | Uncharacterized LOC100306338 | 0.399710 | 0.043262143 |
| 41 | GO:0005623;  GO:0009055;  GO:0015035;  GO:0022900;  GO:0045454 | CC_Cell;  MF_Electron transfer activity;  MF_Protein disulfide oxidoreductase activity;  BP_Electron transport chain;  BP_Cell redox homeostasis | XP_003543019.1 | Glutaredoxin-C9 | 0.402536 | 0.048376068 |
| 42 | GO:0009813;  GO:0016210 | BP_Flavonoid biosynthetic process;  MF_Naringenin-chalcone synthase activity | XP_003531223.1 | Chalcone synthase 1 | 0.403386 | 0.030300188 |
| 43 | GO:0003677;  GO:0003700;  GO:0005634;  GO:0006355 | MF_DNA binding;  MF_DNA-binding transcription factor activity;  CC_Nucleus;  BP_Regulation of transcription, DNA-templated | XP_003535804.1 | Ethylene-responsive transcription factor 1B | 0.404122 | 0.045659229 |
| 44 | GO:0006970;  GO:0006995 | BP_Response to osmotic stress;  BP_Cellular response to nitrogen starvation | XP_006580177.1 | Precursor of CEP14-like | 0.419093 | 0.021020424 |
| 45 | GO:0004601;  GO:0005576;  GO:0006979;  GO:0020037;  GO:0042744;  GO:0046872;  GO:0055114;  GO:0098869 | MF_Peroxidase activity;  CC_Extracellular region;  BP_Response to oxidative stress;  MF_Heme binding;  BP_Hydrogen peroxide catabolic process;  MF_Metal ion binding;  BP_Oxidation-reduction process;  BP_Cellular oxidant detoxification | XP_003540000.1 | Peroxidase P7 | 0.420668 | 0.032503935 |
| 46 | GO:0004864;  GO:0005634;  GO:0005737;  GO:0006952;  GO:0009607;  GO:0009738;  GO:0010427;  GO:0030001;  GO:0032515;  GO:0038023;  GO:0046872;  GO:0080163 | MF_Protein phosphatase inhibitor activity;  CC_Nucleus;  CC_Cytoplasm;  BP_Defense response;  BP_Response to biotic stimulus;  BP_Abscisic acid-activated signaling pathway;  MF_Abscisic acid binding;  BP_Metal ion transport;  BP_Negative regulation of phosphoprotein phosphatase activity;  MF_Signaling receptor activity;  MF_Metal ion binding;  BP_Regulation of protein serine/threonine phosphatase activity | NP_001235344.2 | SRPBCC ligand-binding domain-containing protein | 0.421349 | 0.011042991 |
| 47 | N.A. | N.A. | NP_001235487.1 | Uncharacterized LOC100527140 | 0.422617 | 0.03449019 |
| 48 | GO:0016021 | CC­_Integral component of membrane | NP_001239877.1 | Lea domain-containing protein | 0.426067 | 0.024562035 |
| 49 | GO:0008270;  GO:0009809;  GO:0045551;  GO:0052747;  GO:0055114 | MF_Zinc ion binding;  BP_Lignin biosynthetic process;  MF_Cinnamyl-alcohol dehydrogenase activity;  MF_Sinapyl alcohol dehydrogenase activity;  BP_Oxidation-reduction process | XP_003552158.1 | Probable mannitol dehydrogenase | 0.426202 | 0.04879641 |
| 50 | GO:0005506;  GO:0005783;  GO:0005789;  GO:0009717;  GO:0016021;  GO:0020037;  GO:0033770;  GO:0055114;  GO:0102604;  GO:0102668 | MF_Iron ion binding;  CC_Endoplasmic reticulum;  CC_Endoplasmic reticulum membrane;  BP_Isoflavonoid biosynthetic process;  CC_Integral component of membrane;  MF_Heme binding;  MF_2-hydroxyisoflavanone synthase activity;  BP_oxidation-reduction process;  MF_naringenin,NADPH:oxygen oxidoreductase activity;  MF_liquiritigenin,NADPH:oxygen oxidoreductase activity | NP_001238515.2 | 2-hydroxyisoflavanone synthase | 0.426681 | 0.002268362 |
| 51 | GO:0009813;  GO:0016210 | BP_Flavonoid biosynthetic process;  MF_Naringenin-chalcone synthase activity | NP_001347352.1 | Chalcone synthase CHS4 | 0.427388 | 0.008392124 |
| 52 | N.A. | N.A. | XP_003546461.1 | IQ domain-containing protein IQM1 | 0.429031 | 0.003362309 |
| 53 | GO:0009813;  GO:0016210 | BP_Flavonoid biosynthetic process;  MF_Naringenin-chalcone synthase activity | NP_001347288.1 | Chalcone synthase 5 | 0.430235 | 0.010359182 |
| 54 | GO:0032259;  GO:0042409 | BP_Methylation;  MF_Caffeoyl-CoA O-methyltransferase activity | XP_014619330.1 | Caffeoyl-CoA O-methyltransferase 5 | 0.431202 | 0.048489292 |
| 55 | GO:0004674;  GO:0004715;  GO:0005524;  GO:0016021;  GO:0018108;  GO:0030247 | MF_Protein serine/threonine kinase activity;  MF_Non-membrane spanning protein tyrosine kinase activity;  MF_ATP binding;  CC_Integral component of membrane;  BP_Peptidyl-tyrosine phosphorylation;  MF_Polysaccharide binding | XP_014621437.1 | Leaf rust 10 disease-resistance locus receptor-like protein kinase-like 1.4 | 0.431823 | 0.002866489 |
| 56 | GO:0005516;  GO:0006952;  GO:0009607;  GO:0016021 | MF_Calmodulin binding;  BP_Defense response;  BP_Response to biotic stimulus;  CC_Integral component of membrane | XP_025981389.1 | MLO-like protein 2 | 0.437835 | 0.028438313 |
| 57 | GO:0016301;  GO:0016310 | MF_Kinase activity;  BP_Phosphorylation | XP_003554195.3 | Polygalacturonase inhibitor | 0.442931 | 0.041725331 |
| 58 | GO:0030001;  GO:0046872 | BP_Metal ion transport;  MF_Metal ion binding | XP_003555948.1 | Heavy metal-associated isoprenylated plant protein 20 | 0.445869 | 0.014540791 |
| 59 | GO:0003852;  GO:0005829;  GO:0009098;  GO:0009507 | MF_2-isopropylmalate synthase activity;  CC_Cytosol;  BP_Leucine biosynthetic process;  CC_Chloroplast | XP_003521867.1 | Probable 2-isopropylmalate synthase | 0.447816 | 0.009742692 |
| 60 | GO:0016021 | CC_ Integral component of membrane | NP_001342481.1 | Uncharacterized LOC111138524 | 0.448818 | 0.025433775 |
| 61 | GO:0009813;  GO:0016210 | BP_Flavonoid biosynthetic process;  MF_Naringenin-chalcone synthase activity | XP_003531220.1 | Chalcone synthase 3 | 0.448891 | 0.027297021 |
| 62 | GO:0004675;  GO:0004715;  GO:0005524;  GO:0005886;  GO:0007178;  GO:0018108 | MF_Transmembrane receptor protein serine/threonine kinase activity;  MF_Non-membrane spanning protein tyrosine kinase activity;  MF_ATP binding;  CC_Plasma membrane;  BP_Transmembrane receptor protein serine/threonine kinase signaling pathway  BP_Peptidyl-tyrosine phosphorylation | XP_003536371.1 | Receptor-like cytosolic serine/threonine-protein kinase RBK2 | 0.449524 | 0.014954346 |
| 63 | N.A. | N.A. | XP_006597761.1 | Uncharacterized LOC102660944 | 0.453017 | 0.018156738 |
| 64 | GO:0004791;  GO:0005623;  GO:0009055;  GO:0015035;  GO:0022900;  GO:0045454;  GO:0098869 | MF_Thioredoxin-disulfide reductase activity;  CC_Cell;  MF_Electron transfer activity;  MF_Protein disulfide oxidoreductase activity;  BP_Electron transport chain;  BP_Cell redox homeostasis;  BP_Cellular oxidant detoxification | NP_001238068.1 | Thioredoxin superfamily protein | 0.462889 | 0.014954346 |
| 65 | GO:0004864;  GO:0005634;  GO:0005737;  GO:0006952;  GO:0009607;  GO:0009738;  GO:0010427;  GO:0032515;  GO:0038023;  GO:0080163 | MF_Protein phosphatase inhibitor activity;  CC_Nucleus;  CC_Cytoplasm;  BP_Defense response;  BP_Response to biotic stimulus;  BP_Abscisic acid-activated signaling pathway;  MF_Abscisic acid binding;  BP_Negative regulation of phosphoprotein phosphatase activity;  MF_Signaling receptor activity;  BP_Regulation of protein serine/threonine phosphatase activity | NP_001236562.1 | Uncharacterized LOC100527731 | 0.468840 | 0.013533073 |
| 66 | GO:0005509;  GO:0016021;  GO:0016491;  GO:0055114 | MF_Calcium ion binding;  CC_Integral component of membrane;  MF_Oxidoreductase activity;  BP_Oxidation-reduction process | XP_014620282.1 | External alternative NAD(P)H-ubiquinone oxidoreductase B3, mitochondrial | 0.480426 | 0.039709129 |
| 67 | GO:0098869 | BP_Cellular oxidant detoxification | XP_003523300.1 | Protein RESPONSE TO LOW SULFUR 3 | 0.481930 | 0.03213515 |
| 68 | GO:0009815;  GO:0045486;  GO:0046872;  GO:0055114 | MF_1-aminocyclopropane-1-carboxylate oxidase activity;  MF_Naringenin 3-dioxygenase activity;  MF_Metal ion binding;  BP_Oxidation-reduction process | XP_003545591.1 | 1-aminocyclopropane-1-carboxylate oxidase | 0.483497 | 0.036246555 |
| 69 | GO:0003677;  GO:0005634;  GO:0006355 | MF_DNA binding;  CC_Nucleus;  BP_Regulation of transcription, DNA-templated | NP_001236626.1 | Dof11 | 0.484401 | 0.014770522 |
| 70 | GO:0005524;  GO:0016021;  GO:0016887 | MF_ATP binding;  CC_Integral component of membrane;  MF_ATPase activity | XP_006602893.1 | AAA-ATPase ASD, mitochondrial | 0.484559 | 0.036465005 |
| 71 | GO:0004338;  GO:0005975;  GO:0042973;  GO:0046658 | MF_Glucan exo-1,3-beta-glucosidase activity;  BP_Carbohydrate metabolic process;  MF_Glucan endo-1,3-beta-D-glucosidase activity;  CC_Anchored component of plasma membrane | XP_003540349.1 | Glucan endo-1,3-beta-glucosidase-like | 0.485297 | 0.008209034 |
| 72 | N.A. | N.A. | NP_001242705.2 | Putative PAR1 protein | 0.488322 | 0.013078251 |
| 73 | GO:0005576;  GO:0005618;  GO:0009664;  GO:0016021 | CC_Extracellular region;  CC_Cell wall;  BP_Plant-type cell wall organization;  CC_Integral component of membrane | XP_003552173.1 | Expansin-A4 | 0.489172 | 0.040097884 |
| ***74 | GO:0003700;  GO:0005634;  GO:0009877;  GO:0043565;  GO:0045892 | MF_DNA-binding transcription factor activity;  CC_Nucleus;  BP_Nodulation;  MF_Sequence-specific DNA binding;  BP_Negative regulation of transcription, DNA-templated | XP_003524198.1 | Ethylene-responsive transcription factor ERN3 | 0.491031 | 0.04369066 |
| 75 | GO:0009813;  GO:0016210 | BP_Flavonoid biosynthetic process;  MF_Naringenin-chalcone synthase activity | XP_003531226.2 | Chalcone synthase 5-like | 0.495746 | 0.021118299 |
| ***76 | GO:0003700;  GO:0005634;  GO:0006355;  GO:0043565 | MF_DNA-binding transcription factor activity;  CC_Nucleus;  BP_Regulation of transcription, DNA-templated;  MF_Sequence-specific DNA binding | XP_006576465.1 | Probable WRKY transcription factor 33 | 0.496733 | 0.041697835 |

^a-b^N.A. means Not Annotated by Blast2GO software^8^ or QuickGO (https://www.ebi.ac.uk/QuickGO/).

^b^BP means Biological Process; MF means Molecular Function; and CC means Component Cell.

^c^RNAseq fold changes in the roots of En1282 inoculated with USDA61 versus the roots of En1282 inoculated with *bel2-5* deletion mutant.

***Representative DEGs were validated by qRT-PCR (Supplementary Figure 2).

**Supplementary Table 4.** Soybean seeds, bacterial strains, oligonucleotides and plasmids used in this study

| **Strain** | **Characteristics^a^ or sequences** | **Construction** | **Reference or source** |
| --- | --- | --- | --- |
| Soybean seeds |  |  |  |
| *Glycine max* |  |  |  |
| Enrei | Wild-type |  | ^9,10^ |
| En1282 | Non-nodulating mutant of Enrei derived by EMS treatment, carrying mutant alleles of *GmNFR1* |  | ^9,10^ |
| Bacterial strains |  |  |  |
| *Bradyrhizobium elkanii* |  |  |  |
| USDA61 | Wild-type stain, Pol^r^ |  | Keyser^b^ |
| BErhcJ | USDA61 derivative harbouring insertion in *rhcJ* encoding a membrane protein which consists type III secretion apparatus, defective in type III protein secretion, Pol^r^ , Km^r^, Tc^r^ |  | ^11^ |
| BEttsI | USDA61 derivative harbouring deletion/insertion in *ttsI*, a positive regulator for *tts* operon, region, Pol^r^, Sm^r^, Tc^r^ |  | ^12^ |
| BE2-5 | Tn5 mutant of USDA61, Pol^r^ , Km^r^ |  | ^13^ |
| BEnopL | USDA61 derivate harboring an insertion in the *nopL* region, Pol^r^ , Km^r^ , Tc^r^ |  | ^14^ |
| BEnopM1 | USDA61 derivate harboring an insertion in the *nopM1* region, Pol^r^ , Km^r^ , Tc^r^ |  | ^14^ |
| BEnopP1 | USDA61 derivate harboring an insertion in the *nopP1* region, Pol^r^ , Km^r^ , Tc^r^ |  | ^14^ |
| BEnopP2 | USDA61 derivate harboring an insertion in the *nopP2* region, Pol^r^ , Km^r^ , Tc^r^ |  | ^14^ |
| BEernA (USDA61ΩernA61) | USDA61 derivate harboring an insertion in the *ernA* region, Pol^r^ , Km^r^ |  | ^7^ |
| BEinnB | USDA61 derivate with a deletion of *innB* gene, Pol^r^ |  | ^15^ |
|  |  |  |  |
| BEorf48 | USDA61 derivate harboring an insertion in the *orf48* region, Pol^r^ , Km^r^ , Tc^r^ | The internal region of *orf48* was amplified by PCR using the primers BEorf48_F and BEorf48_R. The PCR product was cloned into the vector pSUPSCAKm^11^. The carrying plasmid was transformed into *E. coli* S17 and was mobilized into *B. elkanii* USDA61 via triparental conjugation pRK2013^16^ as a helper. Mutant was selected on PSY medium containing polymyxin and kanamycin and confirmed by PCR using primer set using the primer set Psuppol-F and Psuppol-R. Pol^r^, Km^r^ , Tc^r^ | This study |
| BEorf91 | USDA61 derivate harboring an insertion in the *orf91* region, Pol^r^ , Km^r^ , Tc^r^ | The internal region of *orf91* was amplified by PCR using the primers BEorf91_F and BEorf91_R. The PCR product was cloned into the vector pSUPSCAKm^11^. The carrying plasmid was transformed into *E. coli* S17 and was mobilized into *B. elkanii* USDA61 via triparental conjugation pRK2013^16^ as a helper. Mutant was selected on PSY medium containing polymyxin and kanamycin and confirmed by PCR using primer set using the primer set Psuppol-F and Psuppol-R. Pol^r^, Km^r^ , Tc^r^ | This study |
| BEnopM2 | USDA61 derivate harboring an insertion in the *nopM2* region, Pol^r^ , Km^r^ , Tc^r^ | The internal region of *nopM2* was amplified by PCR using the primers BEnopM2_F and BEnopM2_R. The PCR product was cloned into the vector pSUPSCAKm^11^. The carrying plasmid was transformed into *E. coli* S17 and was mobilized into *B. elkanii* USDA61 via triparental conjugation pRK2013^16^ as a helper. Mutant was selected on PSY medium containing polymyxin and kanamycin and confirmed by PCR using primer set using the primer set Psuppol-F and Psuppol-R. Pol^r^, Km^r^ , Tc^r^ |  |
| *∆bel2-5* | USDA61 derivate with a deletion of *bel2-5* gene, Pol^r^ | The upstream and downstream regions of *bel2-5* gene were amplified by overlap-extension PCR using primer sets BEorf5208_F1/R1 and BEorf5208_F2/R2, respectively. The PCR product was cloned into plasmid pK18mobsacB^17^ at *EcoR*I/*Hind*III sites and transformed into *E. coli* DH5α. The carrying plasmid was mobilized into *B. elkanii* USDA61 via triparental conjugation using pRK2013^16^ as a helper. The deletion mutant was screened on AG medium containing sucrose 10% and polymyxin and confirmed by PCR. Pol^r^ | This study |
| BeCya | USDA61 derivate with a *bel2-5::cya* fusion, Pol^r^ , Km^r^ , Tc^r^ | The C-terminus of *bel2-5* (_~_0.5kb) was amplified by PCR using primers bel2-5Cya-F and bel2-5Cya-R. The PCR product was cloned into plasmid pSLC5Sm^18^ at *EcoR*I/*Xba*I sites and transformed into *E. coli* DH5α**.** The carrying plasmid was mobilized into *B. elkanii* USDA61 via triparental conjugation using pRK2013 (Figurski and Helinski, 1979) as a helper. The mutant was confirmed on AG medium containing polymyxin, streptomycin, and tetracycline and confirmed by PCR. Pol^r^, Sm^r^ , Tc^r^ | This study |
| BErhcJCya | BErhcJ derivate with a *bel2-5::cya* fusion, Pol^r^ , Km^r^ , Tc^r^ | The C-terminus of *bel2-5* (_~_0.5kb) was amplified by PCR using primers bel2-5Cya-F and bel2-5Cya-R. The PCR product was cloned into plasmid pSLC5Sm^18^ at *EcoR*I/*Xba*I sites and transformed into *E. coli* DH5α**.** The carrying plasmid was mobilized into BErhcJ via triparental conjugation using pRK2013^16^ as a helper. The mutant was confirmed on AG medium containing polymyxin, streptomycin, and tetracycline and confirmed by PCR. Pol^r^, Km^r^, Sm^r^ , Tc^r^ | This study |
| *∆bel2-5*::*bel2-5* | *∆bel2-5* derivate complemented with *bel2-5* gene and its promoter sequence, Pol^r^ , Sm^r^, Sp^r^ | The *bel2-5* gene and its promoter sequence (_~_4.5kb) were amplified by PCR using primers pBjGroEL4:: bel2-5_F and pBjGroEL4:: bel2-5_R. The PCR product was cloned into plasmid pBjGroEL4::DsRed2^19^ at *Sac*I/*Kpn*I and transformed into *E. coli* S17. The carrying plasmid was mobilized into *∆bel2-5* via triparental conjugation using pRK2013^16^ as a helper. The complementation strain confirmed on AG medium containing polymyxin, streptomycin, and spectinomycin and confirmed by PCR. Pol^r^ , Sm^r^, Sp^r^ | This study |
| BEC1286A | USDA61 derivate carrying an amino acid substitution of Cys (C1286) with Ala in the ULP1-like domain of Bel2-5, Pol^r^ , Sm^r^, Sp^r^ | The *bel2-5* C-terminus and downstream region sequences (_~_1.4kb) were amplified by PCR using set primer bel2-5-ULP1-F/R. The PCR product was cloned into plasmid pS18mob at *Bam*HI/*Sph*I and transformed into *E. coli* DH5α and used as a template for site-directed mutagenesis. The substitution of cysteine (C1286) into alanine residue was generated by QuickChange Lightning Site-Directed Mutagenesis Kit (Agilent) using primer set bel2-5C1286A_F/R and transformed into *E. coli* XL-Gold and confirmed by sequencing. The carrying plasmid was mobilized into *B. elkanii* USDA61 via triparental conjugation using using pRK2013^16^ as a helper. The mutant was confirmed on AG medium containing polymyxin, streptomycin, and spectinomycin and confirmed by sequencing. Pol^r^ , Sm^r^, Sp^r^ | This study |
| BEH1231A | USDA61 derivate carrying an amino acid substitution of His (H1231) with Ala in the ULP1-like domain of Bel2-5, Pol^r^ , Sm^r^, Sp^r^ | The *bel2-5* C-terminus and downstream region sequences (_~_1.4kb) were amplified by PCR using set primer bel2-5-ULP1-F/R. The PCR product was cloned into plasmid pS18mob at *Bam*HI/*Sph*I and transformed into *E. coli* DH5α and used as a template for site-directed mutagenesis. The substitution of histidine (H1231) into alanine residue was generated by QuickChange Lightning Site-Directed Mutagenesis Kit (Agilent) using primer set bel2-5H1231A_F/R and transformed into *E. coli* XL-Gold and confirmed by sequencing. The carrying plasmid was mobilized into *B. elkanii* USDA61 via triparental conjugation using using pRK2013^16^ as a helper. The mutant was confirmed on AG medium containing polymyxin, streptomycin, and spectinomycin and confirmed by sequencing. Pol^r^ , Sm^r^, Sp^r^ | This study |
| BED1251A | USDA61 derivate carrying an amino acid substitution of Asp (D1251) with Ala in the ULP1-like domain of Bel2-5, Pol^r^ , Sm^r^, Sp^r^ | The *bel2-5* C-terminus and downstream region sequences (_~_1.4kb) were amplified by PCR using set primer bel2-5-ULP1-F/R. The PCR product was cloned into plasmid pS18mob (Okazaki et al. unpublished) at *Bam*HI/*Sph*I and transformed into *E. coli* DH5α and used as a template for site-directed mutagenesis. The substitution of aspartic acid (D1251) into alanine residue was generated by QuickChange Lightning Site-Directed Mutagenesis Kit (Agilent) using primer set bel2-5D1251A_F/R and transformed into *E. coli* XL-Gold and confirmed by sequencing. The carrying plasmid was mobilized into *B. elkanii* USDA61 via triparental conjugation using using pRK2013^16^ as a helper. The mutant was confirmed on AG medium containing polymyxin, streptomycin, and spectinomycin and confirmed by sequencing. Pol^r^ , Sm^r^, Sp^r^ | This study |
| *Bradyrhizobium* sp. |  |  |  |
| ORS3275 | Wild-type stain, Pol^r^ |  | ^20^ |
| ORS3257::*bel2-5* |  | The *bel2-5* gene and promoter sequences (_~_4.5kb) were amplified by PCR using a set of primer. The PCR product was cloned into plasmid pBjGroEL4::DsRed2^19^ at *Sac*I/*Kpn*I and transformed into *E. coli* S17. The resultant plasmid (pBjGroEL4::*bel2-5*) was mobilized into ORS3257 via triparental conjugation using pRK2013^16^ as a helper. The complementation strain confirmed on AG medium containing polymyxin, streptomycin, and spectinomycin and confirmed by PCR. Pol^r^ , Sm^r^, Sp^r^ | This study |
| *Agrobacterium tumefaciens* |  |  |  |
| GV3101::p35S-*bel2-5*-egfp | *A. tumefaciens* strain GV310 carrying the plasmid p35S-bel2-5-egfp, Sp^r^ | The *bel2-5* gene sequence from the start to the codon before the stop was amplified by PCR using primers GV3101p35S-Bel2-5-gfp_F/R and cloning into the Gateway Entry Vector pCR8/GW/TOPO vector (Thermo Fisher). The PCR product was recombined into the destination vector pB7FWG2,0 (https://gateway.psb.ugent.be) with a C-terminal GFP tag and downstream of a 35S promoter. The recombinant vector (p35S-bel2-5-egfp) was transformed into competent cells of *A. tumefaciens* strain GV3101. The spectinomycin resistant bacteria gene was used to determine the sub-cellular localization of Bel2-5 into *N. benthamiana* leaf cells. Sp^r^ | This study |
| Plasmid |  |  |  |
| pRK2013 | ColE1 replicon carrying RK2 transfer genes; Km^r^, tra |  | ^16^ |
| pSUPSCAKm | Derivative of pSUPPOL2SCA^21^ with a kanamycin resistance gene in the *Dra*I site, oriT of RP4, Tc^r^, Km^r^ |  | ^12^ |
| pBjGroEL4::DsRed2 | DsRed transposon delivery vector, Sm^r^, Sp^r^ |  | ^19^ |
| pCR8/GW/TOPO | Gateway entry vector, Sp^r^ |  | ThermoFisher |
| pK18mobsacB | Mobilizable vector for gene disruption and replacement, Mob+ *sacB*, Km^r^ |  | ^17^ |
| pS18mob | Derivative of pK18mob with *aadA* gene in the position of kanamycin resistant gene, Mob+, Sm^r^, Sp^r^ |  | Okazaki et al. unpublished |
| pSLC5Sm | Derivative of the *cya* fusion plasmid pSLC^22^ with a spectinomycin/streptomycin resistance gene (*aadA*) in the *Dra*I site, Sm^r^, Sp^r^ |  | ^18^ |
| pB7FWG2,0 | Gateway destination vector, Sp^r^ |  | https://gateway.psb.ugent.be |
| Oligonucleotides for mutant constructions | | | |
| BEnopM2_F | 5’- ACCGCGGTGGCGGCCAGGAAAGGGCACAAAACG -3’ |  | This study |
| BEnopM2_R | 5’- CGGGGGATCCACTAGTCCAGGTCAGGGTGATGC -3’ |  | This study |
| BEorf48_F | 5’-ACCGCGGTGGCGGCCCGTTGGATGTTCATACGGC-3’ |  | This study |
| BEorf48_R | 5’-CGGGGGATCCACTAGCGTTCTGTCACAAGACCAC-3’ |  | This study |
| BEorf91_F | 5’-ACCGCGGTGGCGGCCCTACTCAAGCCAATCTGACG-3’ |  | This study |
| BEorf91_R | 5’-CGGGGGATCCACTAGGCTGTGTAAAGTCGGAGAG-3’ |  | This study |
| BEorf5208_F1 | 5’-ACATGATTACGAATTCGCCTATGGTGTCGTTTGCTT-3’ |  | This study |
| BEorf5208_R1 | 5’-GGCGACATCTAGACCGTTCTCACCTTCAAAATA-3’ |  | This study |
| BEorf5208_F2 | 5’-AGAACGGTCTAGATGTCGCCGTGGGCGGATAGC-3’ |  | This study |
| BEorf5208_R2 | 5’-GGCCAGTGCCAAGCTTAGCCGTTTTGGAAGAAACCT-3’ |  | This study |
| bel2-5Cya-F | 5’- GATGCCCGGGGAATTCCCAGCATATCGACAGGGA -3’ |  | This study |
| bel2-5Cya-R | 5’- TTGCTGCATATCTAGACAACCCCTGAGTCGGTTCTG -3’ |  | This study |
| bel2-5Cya-F | 5’- GATGCCCGGGGAATTCCCAGCATATCGACAGGGA -3’ |  | This study |
| bel2-5Cya-R | 5’- TTGCTGCATATCTAGACAACCCCTGAGTCGGTTCTG -3’ |  | This study |
| pBjGroEL4:: *bel2-5*_F | 5’- GGGAACAAAAGCTGGAGCTCGGGGTGACGTGGGTGTCATTCTGCA -3’ |  | This study |
| pBjGroEL4:: *bel2-5* _R | 5’- GCTAGGGCGAATTGGGTACCAGTGCCAAGCTTGCATGCCTA -3’ |  | This study |
| bel2-5-ULP1-F | 5’- CGGTACCCGGGGATCCTCTGCGTTTGATGGGGAAA -3’ |  | This study |
| bel2-5-ULP1-R | 5’- CCAGTGCCAAGCTTGCATGCGCCTGATTCCGGTCCTTC -3’ |  | This study |
| bel2-5C1286A_F | 5’- ACAAAGACGCCGGCATACATAAGTGTTCTGCTGCTGGG -3’ |  | This study |
| bel2-5C1286A_R | 5’- CCCAGCAGCAGAACACTTATGATGCCGGCGTCTTTGT -3’ |  | This study |
| bel2-5-ULP1-F | 5’- CGGTACCCGGGGATCCTCTGCGTTTGATGGGGAAA -3’ |  | This study |
| bel2-5-ULP1-R | 5’- CCAGTGCCAAGCTTGCATGCGCCTGATTCCGGTCCTTC -3’ |  | This study |
| bel2-5H1231A_F | 5’- GAACAGCAGCGACCAAGCGTTGCCGCGGCTATTA-3’ |  | This study |
| bel2-5H1231A _R | 5’- TAATAGCCGCGGCAACGCTTGGTCGCTGCTGTTC-3’ |  | This study |
| bel2-5-ULP1-F | 5’- CGGTACCCGGGGATCCTCTGCGTTTGATGGGGAAA -3’ |  | This study |
| bel2-5-ULP1-R | 5’- CCAGTGCCAAGCTTGCATGCGCCTGATTCCGGTCCTTC -3’ |  | This study |
| bel2-5D1251A_F | 5’- GTCCGCCGTAGGAAGCGTAGTGATAGGCG-3’ |  | This study |
| bel2-5 D1251A _R | 5’- CGCCTATCACTACGCTTCCTACGGCGGAC-3’ |  | This study |
| pBjGroEL4:: *bel2-5*_F | 5’- GGGAACAAAAGCTGGAGCTCGGGGTGACGTGGGTGTCATTCTGCA -3’ |  | This study |
| pBjGroEL4:: *bel2-5* _R | 5’- GCTAGGGCGAATTGGGTACCAGTGCCAAGCTTGCATGCCTA -3’ |  | This study |
| GV3101p35S-Bel2-5-gfp_F | 5’- ATGGATTTCCCCTCGACCAAGTC -3’ |  | This study |
| GV3101p35S-Bel2-5-gfp_R | 5’- ACCCCTGAGTCGGTTCTGCAG-3’ |  | This study |
| pSUPPOL_F | 5’- ATAAACCAGCCAGCCGGAA -3’ |  | ^23^ |
| pSUPPOL_R | 5’- TTCTGACAACGATCGGAGGA -3’ |  | ^23^ |
| Oligonucleotides for qRT-PCR | | | |
| Bel2-5_F | 5’- CGTGATTAATGGCAATCCTG -3’ |  | This study |
| Bel2-5_R | 5’- TGCTGCATCTCTGTTGTTGA -3’ |  | This study |
| NopA_F | 5’- CGCAGCTGGTACTGCTACTG -3’ |  | ^18^ |
| NopA_R | 5’- GAGACGACGCGAAGTTCTACA -3’ |  | ^18^ |
| atpD_F | 5’- GGTCGTCGATCTTCTTGCTC -3’ |  | ^18^ |
| atpD_R | 5’- CGGCGAACACGGAGTAAC -3’ |  | ^18^ |
| SUBI-2.2_F | 5’- AGCTATTCGCAGTTCCCAAAT-3’ |  | ^24^ |
| SUBI-2.2_R | 5’- CAGAGACGAACCTTGAGGAGA -3’ |  | ^24^ |
| GmIPT5_F | 5’- AACATCTCCCCGCGTGTTTG -3’ | XM_003550461.4 PREDICTED: Glycine max adenylate isopentenyltransferase 5, chloroplastic (LOC100778430), mRNA | This study |
| GmIPT5_R | 5’- CTCCTTGTTGTTGCGGTGGTG -3’ |  | This study |
| GmCkHydroxlase_F | 5’- CGTTGTGCTCAAGCAAGTCGT -3’ | XM_003552681.4 PREDICTED: Glycine max cytokinin hydroxylase (LOC100808392), mRNA | This study |
| GmCkHydroxlase_F | 5’- CATTCAGGAGCATCCCCAACA -3’ |  | This study |
| GmACO1_F | 5’- ACTTGGAGAAGCTCAGTGGTGA -3’ | XM_003519400.3 PREDICTED: Glycine max 1-aminocyclopropane-1-carboxylate oxidase (LOC100814810), mRNA | This study |
| GmACO1_R | 5’- CACAGTGTCCAATATGTCATGAGGA -3’ |  | This study |
| GmERN3_F | 5’- CCAATGAGTGCCATGAGGAGG -3’ | XM_003524150.3 PREDICTED: Glycine max ethylene-responsive transcription factor ERN3 (LOC100803832), mRNA | This study |
| GmERN3_R | 5’- TCTCAGGGAAAATGACACGGAA -3’ |  | This study |
| GmWRKY33_F | 5’- GAAACACTTCAACTGAGGGTCAACA -3’ | XM_006576402.3 PREDICTED: Glycine max probable WRKY transcription factor 33 (LOC102666898), mRNA | This study |
| GmWRKY33_R | 5’- GTTTGCTTTGCAGCTTCACTGG -3’ |  | This study |
| GmERF98-F | 5’- ACCACCTTGTGAGACCAGCTT -3’ | NM_001254205.3 Glycine max ethylene-responsive transcription factor ERF098-like protein (LOC100779173), mRNA | This study |
| GmERF98-R | 5’- TCATGCCAGTGACTAACACAACA -3’ |  | This study |

^a^Pol^r^ , polymyxin resistant; Km^r^ , kanamycin resistant; Tc^r^ , tetracycline resistant; Sm^r^ , streptomycin resistant; Sp^r^ , spectinomycin resistant.

^b^United States Department of Agriculture, Beltsville, MD.

**References:**

1. Xiang, Q. W. *et al.* NopD of Bradyrhizobium sp. XS1150 Possesses SUMO Protease Activity. *Front. Microbiol.* **11**, 1–12 (2020).

2. Hotson, A., Chosed, R., Shu, H., Orth, K. & Mudgett, M. B. Xanthomonas type III effector XopD targets SUMO-conjugated proteins in planta. *Mol. Microbiol.* **50**, 377–389 (2003).

3. Kim, J. G., Taylor, K. W. & Mudgett, M. B. Comparative analysis of the XopD type III secretion (T3S) effector family in plant pathogenic bacteria. *Mol. Plant Pathol.* **12**, 715–730 (2011).

4. Kimbrel, J. A. *et al.* Mutualistic Co-evolution of Type III Effector Genes in Sinorhizobium fredii and Bradyrhizobium japonicum. *PLoS Pathog.* **9**, (2013).

5. Staehelin, C. & Krishnan, H. B. Review Article: Nodulation outer proteins: Double-edged swords of symbiotic Rhizobia. *Biochem. J.* **470**, 263–274 (2015).

6. Okazaki, S. *et al.* Identification and functional analysis of type III effector proteins in mesorhizobium Loti. *Mol. Plant-Microbe Interact.* **23**, 223–234 (2010).

7. Teulet, A. *et al.* The rhizobial type III effector ErnA confers the ability to form nodules in legumes. *Proc. Natl. Acad. Sci. U. S. A.* **116**, 21758–21768 (2019).

8. Götz, S. *et al.* High-throughput functional annotation and data mining with the Blast2GO suite. *Nucleic Acids Res.* **36**, 3420–3435 (2008).

9. Francisco, P. B. & Akao, S. Autoregulation and nitrate inhibition of nodule formation in soybean cv. enrei and its nodulation mutants. *J. Exp. Bot.* **44**, 547–553 (1993).

10. Ikeda, S. *et al.* Microbial community analysis of field-grown soybeans with different nodulation phenotypes. *Appl. Environ. Microbiol.* **74**, 5704–5709 (2008).

11. Okazaki, S., Zehner, S., Hempel, J., Lang, K. & Göttfert, M. Genetic organization and functional analysis of the type III secretion system of Bradyrhizobium elkanii. *FEMS Microbiol. Lett.* **295**, 88–95 (2009).

12. Okazaki, S., Kaneko, T., Sato, S. & Saeki, K. Hijacking of leguminous nodulation signaling by the rhizobial type III secretion system. *Proc. Natl. Acad. Sci.* **110**, 17131–17136 (2013).

13. Faruque, O. M. *et al.* Identification of Bradyrhizobium elkanii genes involved in incompatibility with soybean plants carrying the Rj4 allele. *Appl. Environ. Microbiol.* **81**, 6710–6717 (2015).

14. Kusakabe, S. *et al.* Lotus accessions possess multiple checkpoints triggered by different type iii secretion system effectors of the wide-host-range symbiont Bradyrhizobium elkanii USDA61. *Microbes Environ.* **35**, (2020).

15. Nguyen, H. P., Ratu, S. T. N., Yasuda, M., Teaumroong, N. & Okazaki, S. Identification of Bradyrhizobium elkanii USDA61 Type III Effectors Determining Symbiosis with Vigna mungo. *Genes (Basel).* **11**, 474 (2020).

16. Figurski, D. H. & Helinski, D. R. Replication of an origin-containing derivative of plasmid RK2 dependent on a plasmid function provided in trans (plasmid replication/replication origin/trans-complementation/broad host range/gene cloning). *Proc. Nati. Acad. Sc* **76**, 1648–1652 (1979).

17. Schäfer, A. *et al.* Small mobilizable multi-purpose cloning vectors derived from the Escherichia coli plasmids pK18 and pK19: selection of defined deletions in the chromosome od Corynebacterium glutamicum. *Gene* **145**, 69–73 (1994).

18. Nguyen, H. P., Ratu, S. T. N., Yasuda, M., Göttfert, M. & Okazaki, S. InnB, a Novel Type III Effector of Bradyrhizobium elkanii USDA61, Controls Symbiosis With Vigna Species. *Front. Microbiol.* **9**, 3155 (2018).

19. Hayashi, M. *et al.* A thaumatin-like protein, Rj4, controls nodule symbiotic specificity in soybean. *Plant Cell Physiol.* **55**, 1679–1689 (2014).

20. Okazaki, S. *et al.* Rhizobium-legume symbiosis in the absence of Nod factors: Two possible scenarios with or without the T3SS. *ISME J.* **10**, 64–74 (2016).

21. Krause, A., Doerfel, A. & Göttfert, M. Mutational and Transcriptional Analysis of the Type III Secretion System of *Bradyrhizobium japonicum*. *Mol. Plant-Microbe Interact.* **15**, 1228–1235 (2002).

22. Wenzel, M., Friedrich, L., Göttfert, M. & Zehner, S. The Type III-Secreted protein NOpEl affects symbiosis and exhibits a calcium-dependent autocleavage activity. *Mol. Plant-Microbe Interact.* **23**, 124–129 (2010).

23. Nguyen, H. P., Miwa, H., Kaneko, T., Sato, S. & Okazaki, S. Identification of Bradyrhizobium elkanii genes involved in incompatibility with Vigna radiate. *Genes (Basel).* **8**, 374 (2017).

24. Yasuda, M. *et al.* Effector-triggered immunity determines host genotype-specific incompatibility in legume-rhizobium symbiosis. *Plant Cell Physiol.* **57**, 1791–1800 (2016).
